# Mid-mantle discontinuities beneath subduction zones linked to depth-dependent deformation of bridgmanite

**DOI:** 10.1038/s41467-026-78212-6

**Published:** 2026-10-05

**Authors:** John Keith Magali, Sébastien Merkel, Angelo Pisconti, Jeffrey Gay, James Wookey, Christine Thomas

**Affiliations:** 1 Institute of Geophysics, University of Münster, Münster, Germany; 2 Univ. Lille, CNRS, INRAE, Centrale Lille, UMR 8207 - UMET - Unité Matériaux et Transformations, Lille, France; 3 Dept. of Geosciences, Princeton University, Princeton, NJ USA; 4 School of Earth Sciences, University of Bristol, Bristol, UK; 5 Geological Survey of Denmark and Greenland, Copenhagen, Denmark

**Keywords:** Seismology, Geodynamics, Mineralogy, Geophysics

## Abstract

Bridgmanite, the main mineralogical phase in the Earth’s lower mantle, is intrinsically anisotropic and may therefore generate seismic anisotropy in actively-deforming regions, particularly subduction zones. The bulk of the lower mantle is widely regarded as well-mixed but recent seismic observations suggest structures at mid-mantle depths whose origin remains unclear. Here, we propose a mechanism that could generate seismic discontinuities beneath subduction zones with depth distributions consistent with some of the observed mid-mantle reflectors. By coupling thermo-mechanical subduction models with pressure-dependent fabric calculations, we show that gradual slip transitions in bridgmanite with depth can manifest anisotropic discontinuities near 1000 km, which can deepen depending on the extent of accumulated deformation. This discontinuity is most pronounced in regions of strong slab-driven flow as revealed by its reflection coefficients, the strength of which varies with distance and azimuth. While our models predict  ~ 1.5% contrast in fast versus slow shear wave velocities in terms of radial anisotropy, our full waveform simulations show that the discontinuity is detectable with array seismological methods. These results suggest that mid-mantle reflectors near subduction zones may be strain-induced, providing an additional mechanism that links mantle flow, mineral physics, and seismic observations.

## Abstract (web summary)

This study shows that changes in the deformation mechanism of bridgmanite beneath subduction zones can generate seismic discontinuities near 1,000 km depth, providing an explanation for observed mid-mantle reflectors.

## Introduction

The dynamics of descending plates plays a central role in the mixing of recycled material into the mantle by imposing intense deformation on the surrounding rocks. This deformation fosters the evolution of textured fabrics and, should dissolution processes be limited, a mechanically-mixed mantle leads to the formation of laminated structures with distinct contrasts in densities and seismic properties. Such heterogeneities, spanning a wide range of length scales, are potential sites for the reflection and conversion of seismic waves. Understanding where these seismic reflections and conversions originate, therefore, provides key insight into the nature of the underlying mineralogical boundaries and their relationship to mantle dynamics.

Unlike mantle transition zone (MTZ) discontinuities^1,2^, which are governed by pressure-induced phase transformations in the olivine system^3^, the origin of mid-mantle (660–2000 km) seismic structures is poorly understood. Seismic reflectors within this depth range have been documented globally using SS precursors (i.e., S-wave underside reflections off depth *d*)^4–6^, hereafter referred to as S^*d*^S, including beneath subduction zones^7,8^ where scattering of converted waves have also been observed^9–14^. These signals, inclusively, span a broad depth interval (~750–1700 km), with some sparse detections below 1900 km^13^. It is unclear whether the signals originate from coherent discontinuities or instead produced by small-scale heterogeneities that scatter seismic energy. As such, they are oftentimes attributed to reflections off the slab interface^6^, accumulations of neutrally-buoyant subducted crust^15^, and/or phase transition of secondary phases^7,16^. However, these interpretations cannot account for observations occurring far beneath slab interfaces^6,17^, underscoring the enigmatic nature of these mid-mantle features.

In subduction zones, S^*d*^S also consistently reveal a prominent seismic discontinuity across 1000 km, and have thus often been associated with the so-called mid-mantle discontinuity^4–6,18^. Global tomographic models, in regions including beneath Indonesia and the Marianas, likewise show sharp velocity contrasts at similar depths^18^. These features are commonly interpreted as the depth at which some sinking slabs tend to flatten^19,20^, and in a different tectonic environment, buoyant upwellings tend to deflect^21^. Efforts to explain the nature of the mid-mantle discontinuity span a range of hypotheses that are all thermo-chemical in origin^22–24^. Evidence for the existence of such a layer appears robust, although it does not seem to be a global feature^6^.

Seismic anisotropy arising from crystallographic preferred orientation (CPO) of intrinsically anisotropic mantle minerals has long been recognized^25^. The robust evidence for large-scale (>100 km) anisotropy in high-stress regions^26,27^ indicates that deformation-driven fabrics persist to great depths^28^. This implies that some mid-mantle features may owe their seismic expression to anisotropic layering rather than solely to compositional contrasts. In the upper mantle (<410 km), the Lehmann discontinuity has been suggested to be due to changes in the intrinsic anisotropy of olivine because of a shift in its deformation mechanism (dislocation to diffusion) at ~ 200 km^29,30^. Subsequent studies further explored this idea^31^, showing that S^*d*^S can help characterize anisotropic discontinuities through amplitude and polarity variations. In the lowermost mantle (>2800 km), anisotropic layering has also been proposed as a possible explanation for deep reflections and shear wave splitting adjacent to large-low velocity provinces^32^. In the mid-mantle—which is the scope of this study—some seismological observations have likewise been linked to anisotropic discontinuities resulting from the entrainment of mantle material via subduction ^10^.

Seismic anisotropy in the mid-mantle is not uniformly distributed but is particularly pronounced beneath subduction zones^27,33–35^, with some even reporting its existence below 1900 km^36^. Bridgmanite ([Mg,Fe]SiO_3_), which comprises ~ 80% of the lower mantle in the pyrolite model^37^, is intrinsically anisotropic and likely the primary phase that manifests CPO during dislocation creep^38,39^. While diffusion creep is cited as the dominant mechanism in the average lower mantle^40^, numerical models suggest that dislocation creep remains an active process in regions associated with large tectonic stresses^28,36,41^. This motivated the re-evaluation of the active slip systems of bridgmanite in a pyrolitic composition by conducting deformation experiments at in situ pressures and temperatures^42,43^. Their findings reveal a deformation pattern previously predicted in atomic scale Peierls stress calculations^44,45^, characterized by a depth-dependent behavior driven by pressure-induced slip transitions under lower mantle conditions.

Here, we investigate the effect of the depth-dependent deformation behavior of bridgmanite on large-scale seismic anisotropy in the mid-mantle beneath a subduction zone. We use the recently-constrained pressure-dependent bridgmanite fabric to perform self-consistent forward modeling that combines 2D thermo-chemical modeling of intraoceanic subduction with mantle fabric modeling. We report how pressure-induced slip transitions in bridgmanite could produce a seismic discontinuity in the mid-mantle. We then evaluate seismic wave reflections at the discontinuity, and further test its detectability using full waveform modeling. Finally, we present the implications of a pressure-dependent fabric on large-scale S-wave anisotropy, and compare our results with single-fabric calculations from current literature, seismic tomography, and shear wave splitting observations. In light of our findings, we propose a mechanism that could generate seismic discontinuities in the mid-mantle beneath subduction zones whose depth distribution broadly matches mid-mantle seismic observations. These discontinuities are purely deformation-driven, providing an additional mechanism to explain mid-mantle reflectors in high-stress regions.

## Results

### Deformation-induced seismic discontinuity in the mid-mantle

**Fig. 1:**
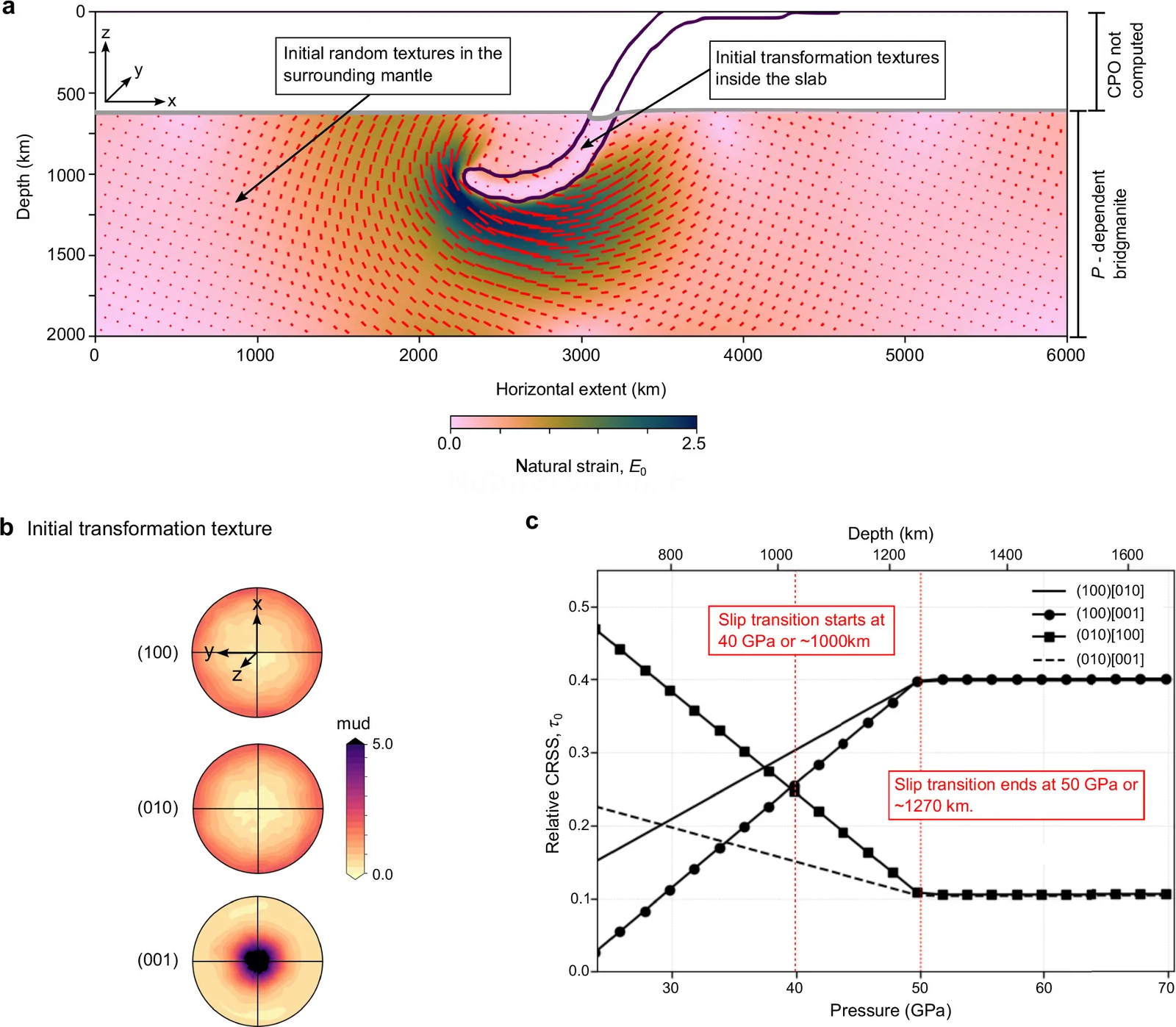
Some properties of the anisotropy model presented in this study. **a** Amount of accumulated deformation induced by the deeply flattened slab model in terms of the natural strain *E*_0_ defined as ${E}_{0}=\frac{1}{2}\ln ({\zeta }_{1}/{\zeta }_{3})$ where *ζ*_1_ and *ζ*_3_ are the long and short axes, respectively, of the finite strain ellipse (fse). Red arrow-less vectors correspond to the orientation of the fse with its length scaled by *E*_0_. When computing crystallographic preferred orientation (CPO), initial random textures are prescribed in the surrounding mantle, whereas initial transformation textures (**b**) are inside the slab. **b** Pole figures of the transformation texture used in this study expressed in multiples of uniform distribution (mud). **c** Relative critically-resolved shear stress (CRSS, *τ*_0_) in a given slip system as a function of pressure *P* inferred from ref. ^43^. Note that all active slip systems CRSS vary with *P*, only that the four most active ones are plotted to avoid clutter. The transition in slip system is progressive between 24 and 50 GPa, corresponding to depths between 670 and 1250 km, with an inversion of CRSS between easiest slip on (100)[001] to (010)[100] at 40 GPa (i.e., 1050 km depth).

Using a pressure(*P*)-dependent bridgmanite fabric^43^ (Fig. 1c), we couple a 2D thermo-mechanical free-subduction model with mantle fabric calculations to predict large-scale anisotropy generated by a slab flattened near 1000 km depth (i.e., the deeply flattened slab model; see “Methods”). In addition, we also explore a shallowly stagnating slab scenario where the slab flattens and then subsequently trapped near the 660-km discontinuity; results from this configuration are presented in the Supplementary Information. We assume a pyrolitic lower mantle where bridgmanite solely controls CPO development, as that of davemaoite (Ca-perovskite) and ferropericlase remains incoherent under lower mantle conditions^43^. The latter phases are, thus, treated as isotropic. Random textures are prescribed in the ambient mantle (Fig. 1a), while intraslab anisotropy is computed by imposing initial transformation textures (i.e., the CPO developed during the ringwoodite-bridgmanite phase transformation)^42^ (Fig. 1b). We refer the reader to the Supplementary Information for a detailed discussion of the modeled intraslab anisotropy. Because the dominant deformation mechanism is difficult to constrain^46^, we apply the total macroscopic velocity gradient to compute CPO. The computed natural strains (i.e., amount of accumulated deformation in terms of the ratio between the long and short axes of the finite strain ellipse) (Fig. 1a), nonetheless, correlates with the fraction of deformation accommodated by dislocation creep in the lower mantle^47^. Finally, we use the viscoplastic self-consistent (VPSC) model^48^ for CPO calculations. While this does not incorporate dynamic recrystallization, models based on VPSC theory capture the primary features of the observed anisotropy despite the exclusion of recrystallization textures^49–52^.

**Fig. 2:**
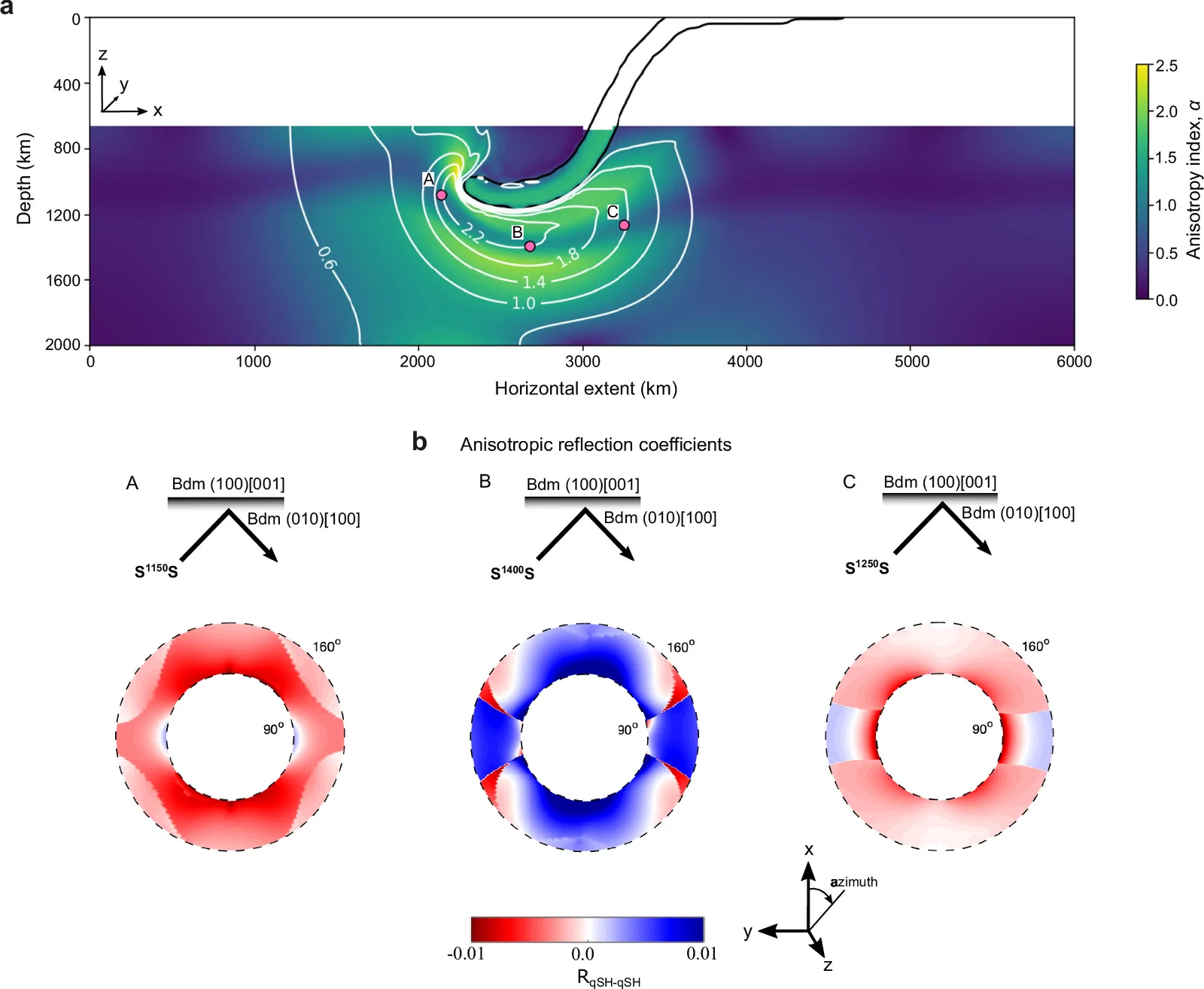
Deformation-induced seismic discontinuity in the mid-mantle. **a** Anisotropy index *α* (i.e., percentage of total anisotropy) expressed as the norm of the deviatoric part of the elastic tensor relative to the norm of its isotropic part (see “Methods”). Solid white lines mark isocontours of the natural strain *E*_0_. Solid purple circles are the selected interfaces: Location A, Location B, and Location C along the anisotropic discontinuity where reflection coefficients are to be computed. **b** Pole figure representations, in each location, of quasi horizontally polarized S-wave (qSH) reflection coefficients *R*_qSH-qSH_ as a function of azimuth and epicentral distance (90°–160°). The reflection coefficients are reminiscent of the S^1150^S, S^1400^S, and S^1250^S phases. Azimuth is measured clockwise relative to the *x* axis of the geodynamic model (see coordinate axis inset). Diagrams above are cartoon representations of underside reflections off the discontinuity separated by a layer of (100)[001] bridgmanite over (010)[100] bridgmanite. Note the apparently sharp seismic boundary despite a continuous transition in bridgmanite slip systems in Fig. 1c.

When the entrained mantle accumulates strain due to slab-driven deformation, the *P*-induced slip transitions in bridgmanite mainly produce an anisotropic discontinuity around the slab that progressively weakens with distance away from the slab into the surrounding mantle. Mapping the anisotropy index *α* (i.e., a scalar value that quantifies the percentage of total anisotropy in the elastic tensor; see “Methods”) reveals this feature as zones in the surrounding mid-mantle (>1000 km) where *α* exhibits a spatially persistent local minimum (Fig. 2a).

In the mantle wedge and subslab regions, the anisotropic discontinuity starts to emerge near ~ 1000 km (Fig. 2a). This corresponds to the depth where the critically-resolved shear stress (CRSS) values of the (100)[001] and (010)[100] slip systems intersect (dashed red line at 40 GPa in Fig. 1c), marking the onset of a transition in the dominant slip system that accommodates strain. Approaching the slab, the discontinuity deepens progressively, eventually reaching its greatest depth of ~ 1400 km directly beneath the slab. Its sharpness (i.e., half-width of the anisotropic discontinuity; see “Methods”) is well-correlated with increasing deformation in the surrounding mantle despite the smooth transition in bridgmanite slip systems with pressure. For instance, Locations A and B (purple circles in Fig. 2a) exhibit relatively sharper anisotropic discontinuities (half-widths of ~ 40 km and ~ 54 km, respectively) owing to larger natural strains at the interface (*E*_0_ > 2) than that of Location C (half-width of ~ 80 km), consistent with its lower strain (*E*_0_ ~ 1.4). Consistent with this observation, the shallowly stagnant slab model exhibits a markedly weaker discontinuity at ~ 1000 km (Supplementary Fig. 2a) under reduced lower-mantle strains (Supplementary Fig. 1), despite its persistence at that depth.

We next assess the seismic detectability of the anisotropic discontinuity by considering both reflection coefficients at selected interfaces along the discontinuity and a full-waveform modeling approach.

### Reflection coefficients at selected interfaces along the discontinuity

At an anisotropic interface, the elastic properties of a medium vary with propagation direction depending on the orientation of the preferentially-aligned minerals, such that the reflection coefficients vary as a function of azimuth^53–55^. We therefore investigate the local seismic properties of the anisotropic discontinuity by calculating azimuthally-varying reflection coefficients. We base our computations on S^*d*^S phases, whose occurrence across multiple period bands^6,7,56^ enables sensitivity to a broad range of seismic structures, including weak or gradational seismic discontinuities^5,30^ and anisotropic discontinuities even without an imposed isotropic velocity or density jump^31^.

For each azimuth, we compute reflection coefficients at the underside of the anisotropic discontinuity following the formulations of refs. ^53,57–59^ (see “Methods”). Calculations are performed at selected interfaces defined by Locations A, B, and C (Fig. 2a), where (1) strong anisotropy contrasts are expected, and (2) different flow orientations are anticipated to produce distinct CPO patterns. Epicentral distances of 90°–160° are considered, corresponding to the range over which S^*d*^S phases are commonly observed. To visualize the reflection coefficients as functions of both distance and azimuth, we present the results using a pole-figure representation adapted from ref. ^55^ (Fig. 2b). Similar calculations were performed for the P^*d*^P phase (i.e., P-wave underside reflection at depth *d*)—for which we found negligible reflection coefficients (Supplementary Fig. 3a). This indicates a predominantly shear-wave sensitivity to the anisotropic discontinuity.

Our results show that quasi(q)-S wave (i.e., S-wave propagating in anisotropic media with direction-dependent velocity and particle motion^60^) reflections exhibit distance and polarity variations that are unique to each deformation pattern. At Location A, where deformation is dominated by poloidal flow, qSH-qSH reflections display negative polarities for all azimuths, that become weaker with epicentral distance. In contrast, at Location B, slab-driven flattening of entrained mantle aggregates produces predominantly positive qSH-qSH reflections over most azimuths, with negative polarities confined to narrow girdles along the NE-SW and NW-SE directions at larger distances. Finally, at Location C, smaller accumulated strain results in a weaker discontinuity, yielding the smallest reflection coefficients among the three locations (*R*_qSH-qSH_ < ~ 0.005) and may thus fall below its seismic detectability limit. Notably, the values at Location B are of similar order of magnitude to the reflection coefficients computed for a bridgmanite-bridgmanite textural interface, previously used to interpret SdS phases in the lowermost mantle^32^, and to that of S^*d*^S phases generated by MTZ discontinuities^54^. We also report negligible reflection coefficients at the anisotropic discontinuity for the shallowly stagnant slab model (Supplementary Fig. 2b) due to low deformation in the surrounding mantle beneath it (Supplementary Fig. 1). These results suggest that deformation-induced anisotropic discontinuities in the mid-mantle may be seismically observable where strain is sufficiently accumulated.

### Seismic detectability using array analysis of synthetic SS precursors

Our anisotropic discontinuity model produces azimuthally-varying reflection coefficients for S^*d*^S phases with reflection amplitudes reaching up to *R*_qSH-qSH_ ~ 0.02 where *E*_0_ > 2.0. Determining whether such reflection amplitudes are visible in seismic data requires array analysis to help identify the expected weak S^*d*^S signal generated by the anisotropic discontinuity. To test its seismic detectability, we perform 3D full waveform simulations of mid-mantle anisotropy^61^. We analyze synthetic waveforms between the Sdiff and the SS time window to search for the S^*d*^S phase using array seismological methods^62^ (see “Methods”).

**Fig. 3:**
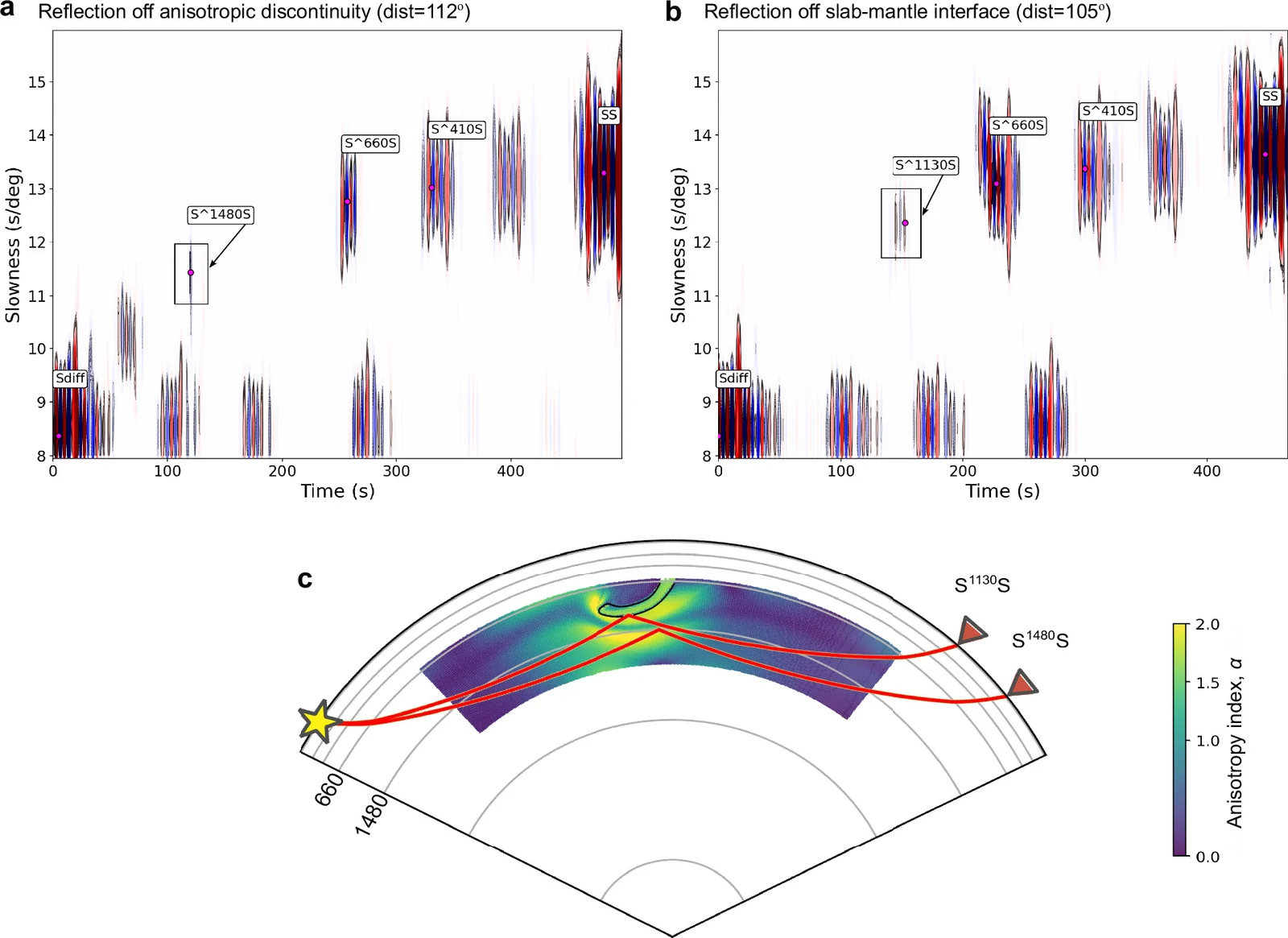
Detectability of the anisotropic discontinuity using SS precursors. **a** Slowness vespagram for the fully-anisotropic mantle model. **b** Same as (**a**) but for an isotropic model with harzburgitic slab velocity perturbations included. Insets indicate phase arrivals predicted using 1D ray tracing over the isotropic Preliminary Reference Earth Model (PREM). Purple circles in (**a**) and (**b**) refer to the maximum amplitude of the phase arrivals. The arrivals of the synthetic mid-mantle SS precursors, S^1480^S and S^1130^S, are highlighted by boxes. Dist pertains to source-to-receiver epicentral distance in degrees (°). **c** Theoretical ray paths of S^1130^S and S^1480^S, with the anisotropy index *α* superimposed.

Figure 3 shows slowness vespagrams of the processed synthetics. For a model that includes only anisotropic velocity contrasts from the induced texture and no isotropic velocity perturbations associated with the slab, the resulting anisotropic discontinuity generates a clear S^*d*^S arrival at 112° (Fig. 3a). Here, we report the S^*d*^S/SS amplitude ratio to be 0.0084. Although this phase is weak, its S^*d*^S/SS amplitude ratio exceeds the global detectability threshold of 0.0065 reported by ref. ^6^. At a slightly larger epicentral distance (117°), we also detect the discontinuity at a slightly shallower depth, but with a reversed S^*d*^S polarity relative to SS (Supplementary Fig. 4a). At this distance, the reflection coefficients predict negative polarities for all azimuths (Supplementary Fig. 4b). No signal from the anisotropic discontinuity is detected at shorter distances (<110°). For the same mantle model but purely isotropic with perturbations relative to PREM restricted to the harzburgitic slab, a much shallower S^*d*^S phase emerges at a shorter distance (~105°; Fig. 3b), arising from the impedance contrast across the slab-mantle interface. Their theoretical ray paths are shown in Fig. 3c.

For this specific geodynamical setup, our results imply that the observability and apparent depth of the S^*d*^S phase not only depend on epicentral distance, but also on slab geometry, and the geometry of the anisotropic discontinuity associated with slab-induced deformation. For example, prolonged horizontal slab flattening in the mid-mantle could reshape the discontinuity to align roughly parallel to the base of the slab. In such a case, both the slab-mantle interface and the anisotropic discontinuity could simultaneously produce a shallow and a deep reflector, respectively, offering a plausible explanation for the observed double reflectors at mid-mantle depths^6^.

### Implications for large-scale S-wave anisotropy

**Fig. 4:**
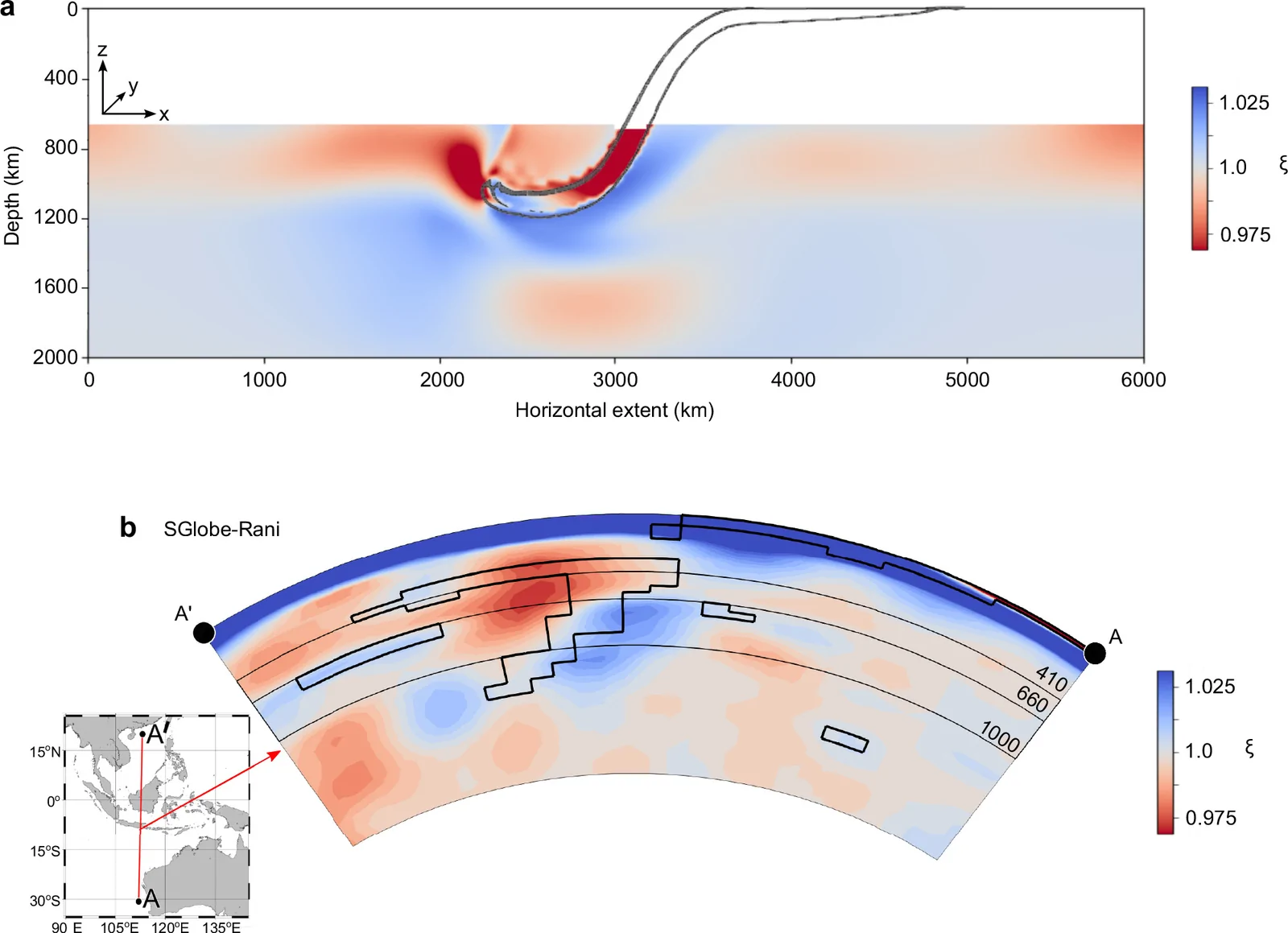
Radial anisotropy in the lower mantle beneath a subduction zone. **a** Predicted patterns of large-scale radial anisotropy—in terms of the radial anisotropy parameter $\xi={V}_{SH}^{2}/{V}_{SV}^{2}-$ in the lower mantle for the deeply flattened slab model from pressure-dependent bridgmanite fabrics. Here, *V*_*SH*_ and *V*_*SV*_ are the horizontally polarized and vertically polarized S-waves, respectively, where both propagate horizontally. **b** Cross section of *ξ* in the Western Java subduction zone from the anisotropic tomography model SGLOBE-Rani^63^. Solid black contours delineate the slab, which is defined by S-wave velocity anomalies in the range +0.75–5.0%, also obtained from SGLOBE-Rani. The thinner solid black lines mark the depths 410, 660, and 660 km relative to the Earth’s surface. Inset maps the $A-{A}^{{\prime} }$ cross section.

We first compare our results with predictions from single-fabric models and radial anisotropy tomography. In the uppermost lower mantle (660–1200 km), our predictions of S-wave radial anisotropy in terms of the parameter $\xi={V}_{SH}^{2}/{V}_{SV}^{2}$ (Fig. 4a) based on a *P*-dependent fabric generally match tomographic images of *ξ* around slabs that impinge the lower MTZ such as the Western Java subduction zone^63^ which exhibits a similar subduction style (Fig. 4b). Likewise, our predictions for the shallowly stagnant slab model also correlate well with that of subduction zones that exhibit similar styles such as beneath Honshu (Supplementary Fig. 5). In general, faster *V*_*SH*_ anomalies are localized beneath the slab that extend towards the subslab region while faster *V*_*SV*_, whose largest values concentrate near the slab tip, can mostly be found in the mantle wedge.

Previous studies applying kinematic approaches to model mantle fabric evolution explored various bridgmanite slip systems to explain observed radial anisotropy patterns in the lower mantle^28,47,64^. First, by imposing (100)[001] as the softest slip system in bridgmanite, they reproduced many large-scale features but did not capture the pronounced *V*_*SH*_ > *V*_*SV*_ between 660 and 1000 km observed in the subslab mantle. Secondly, shifting the softest slip system to (100)[010] improved their subslab radial anisotropy predictions, but also produced strong *V*_*SH*_ > *V*_*SV*_ everywhere, including the mantle wedge, where observations indicate *V*_*SV*_ > *V*_*SH*_. Finally, applying (010)[001] or (001)[010] yielded *V*_*SV*_ > *V*_*SH*_ everywhere, eliminating the strong *V*_*SH*_ > *V*_*SV*_ subslab signature altogether. By incorporating *P*-dependent bridgmanite fabrics, our models allow smooth transitions between deformation modes, resulting in a closer match to S-wave radial anisotropy tomography. This suggests that the *P*-dependent behavior of bridgmanite deformation plays a key role in the formation of radially-anisotropic structures in the lower mantle.

**Fig. 5:**
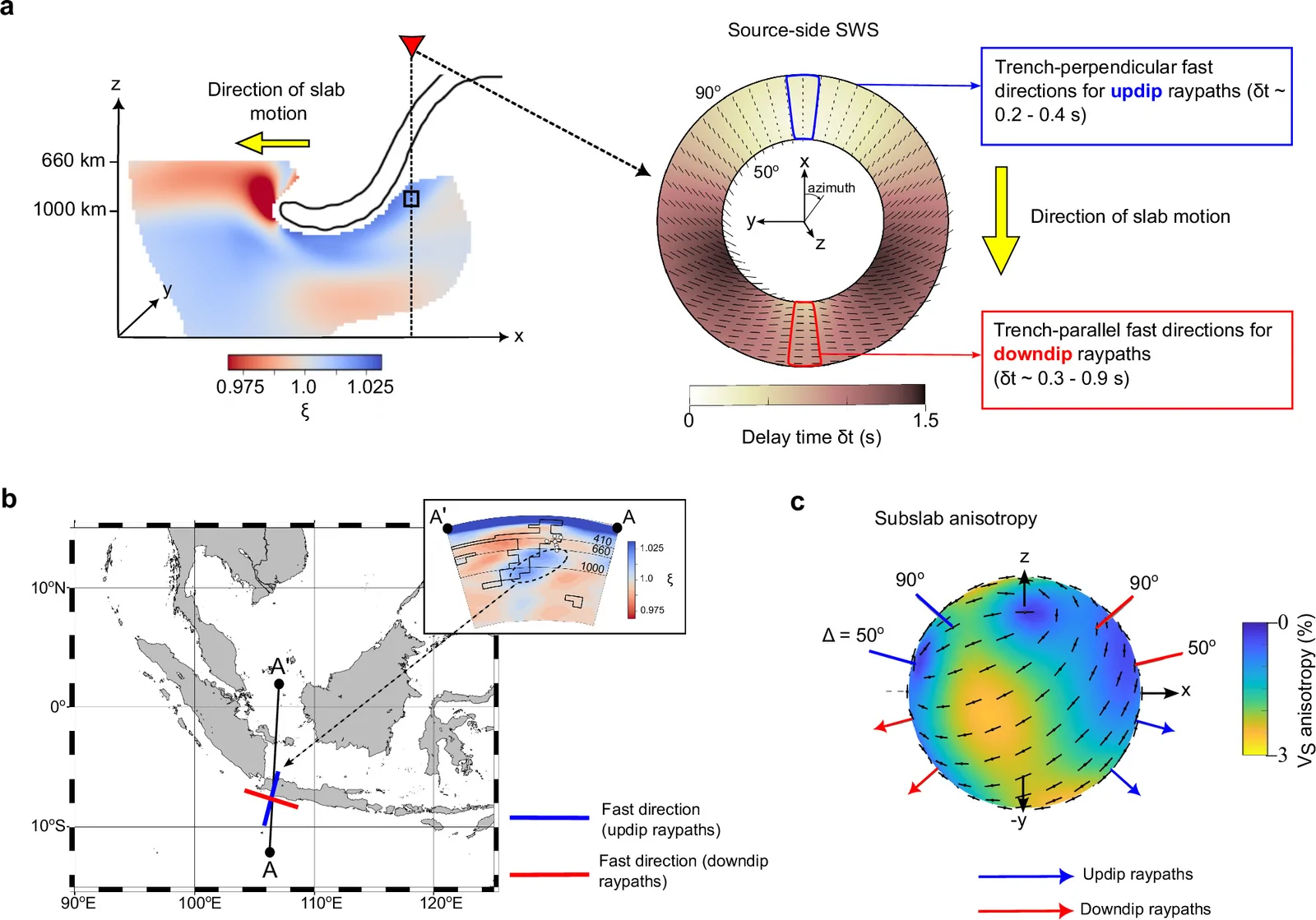
Shear wave splitting in the subslab mantle. **a** Left panel: Masked radial anisotropy parameter *ξ* in regions where natural strains *E*_0_ exceed 0.5. Vertical dashed line refers to the depth distribution of anisotropy in the subslab mantle used to compute effective shear wave splitting (SWS). Right panel: Effective SWS as a function of epicentral distance (50°–90°) and azimuth (0–360°) projected at the surface near the trench (the red inverted triangle of the left panel). Arrow-less vectors in the polar plot refer to fast polarizations relative to zero azimuth (i.e., *x* axis), whereas color gradients pertain to delay time strength *δt* in second (s). Horizontal projection of slab dip is guided by the yellow arrow. **b** General source-side SWS patterns observed underneath Western Java, adapted from ref. ^67^. Fast polarizations vary depending on the direction of the ray traversing through the subslab mantle; blue in the case of updip (i.e., propagation direction of the direct *S* phase opposite to the horizontal projection of the slab dip), and red for downdip ray paths. Inset shows observed *ξ* from the anisotropic tomography model SGlobe-rani along the $A-{A}^{{\prime} }$ cross section. **c** Spherical plot of S-wave (*V*_*S*_) anisotropy from an elastic tensor extracted from a point along the subslab depth profile (dark hollow square in (**a**)). Anisotropy amplitude is expressed in percent; whereas orientations are indicated by the dark arrow-less vectors. Arrows piercing through the sphere pertain to the direction of rays incident to this region for epicentral distances Δ = 50° and 90° (i.e., the distance range typically used in source-side SWS studies).

We then model shear wave splitting (SWS) and compare our results with observations beneath Western Java. Source-side SWS in the subslab mantle generally reveals trench-parallel fast directions for downdip ray paths while nearing trench-perpendicular fast directions for updip ray paths (i.e., propagation direction opposite to subduction geometry) (Fig. 5b)^34,65–67^. Effective SWS predictions in the subslab region of our anisotropy model are consistent with these observations as shown by the trench-parallel and trench-perpendicular fast polarization axes in both downdip and updip directions, respectively (Fig. 5a). By inspecting an elastic tensor at one location in the subslab mantle, we further find that the fast axes of S-wave anisotropy exhibit trench-parallel in the downdip and trench-perpendicular in the updip directions for piercing S-waves originating at epicentral distances between 50° and 90° (Fig. 5c). Because the effective fast polarization reflects the path-integrated CPO across multiple layers making up the subslab anisotropy region^68^, a *P*-dependent bridgmanite fabric is thus sufficient to explain the observed SWS patterns.

The delay times between the fast and slow quasi-S waves computed for our anisotropy model, however, appear underestimated (line plots in Supplementary Fig. 6). This discrepancy likely reflects the inherently large uncertainties surrounding delay time estimates in our modeling and in the observations: (1) In the subslab mantle, other minerals may coexist within the modeled depth range (see “Methods”) such as akimotoite^69^ and phase D^70^. While their depth extent and stability remain uncertain, these phases are largely anisotropic under mid-mantle conditions^71,72^. Assuming these minerals develop spatially uniform fast-axis orientations, they may also contribute to the path-integrated splitting signal. Constructive interference among anisotropic layers with different fabrics could then increase the observed delay times. (2) Grain-scale shape preferred orientation (SPO), such as preferentially-aligned conduits with highly-contrasting seismic velocities may also generate extrinsic anisotropy due to finite frequency effects in seismic data. In the mid-mantle, effective medium theory predicts that SPO-induced fabrics generate radial anisotropy patterns similar to that of a (100)[001] bridgmanite beneath subduction zones^28,47^. This implies that combined CPO and SPO sources can increase the effective anisotropy^52^, thus also leading to increased delay times. (3) Complex receiver-side anisotropy (i.e., those that deviate from an isotropic or a horizontally-transverse isotropic medium) biases delay-time estimates, even after corrections for upper mantle anisotropy^73^. (4) Finally, source-side SWS observations assume similar receiver-side ray paths for S and SK(K)S phases to correct for receiver-side anisotropy. At shorter epicentral distances, the incidence angle of S increases, and hence its receiver-side path may deviate further from the SK(K)S path, which introduces additional uncertainties when correcting for non-null receiver-side anisotropy^66^.

## Discussion

We model frequencies matching the observed dominant frequency of S^*d*^S. Whether the discontinuity persists at higher frequencies is beyond this work. Nevertheless, a previous study demonstrated that lower frequencies spatially-average over larger regions and may integrate incoherent CPO directions, whereas higher frequencies tend to sample more locally coherent CPO, thereby increasing the signature of anisotropy in the seismic signals^74^. Mid-mantle scattering around slabs, for instance, is typically investigated in the 0.2–2 Hz band^10,13^. Although our anisotropic discontinuity mechanism can reasonably explain the depth range of such observations, reproducing scattering at such frequencies would likely require a sharper discontinuity.

One end-member scenario that can promote the development of a sharper anisotropic discontinuity is to impose an abrupt change in CRSS at 50 GPa (~1270 km depth), rather than the gradual *P*-induced slip transition model (Fig. 1c). For bridgmanite aggregates crossing the 50 GPa boundary, this rapidly overprints their pre-existing textures with that of the new (010)[100] fabric, resulting in sharper anisotropic discontinuities with an average half-width of ~ 35 km along the horizontal extent of 2150–3300 km (Supplementary Fig. 7: bottom panel). This, however, overestimates the strength of anisotropy over large areas when compared to radial anisotropy tomography^28^ where *ξ* ~ 0.985–1.02. In our end-member model, we predict strong negative radial anisotropy *ξ* < 0.96 near the mantle wedge and strong positive radial anisotropy *ξ* ~ 1.03 beneath the slab (Supplementary Fig. 7: top panel) that are not present in our gradual transition model (Fig. 4a). In natural subduction systems, several geodynamic scenarios could result in a sharper anisotropic discontinuity. Prolonged lateral gliding of a flattened slab near 1000 km, as in the case of the Cocos slab^19^, can intensify (100)[001] bridgmanite textures immediately beneath the slab due to progressive shear deformation. This potentially creates a sharper anisotropic discontinuity beneath the entrained mantle from above, and a moderately developed (010)[100] bridgmanite textures below it, as in the current model. A viscosity jump near 1000 km can also promote flattening by forcing the slab to drape across the high viscosity layer^19^. This localizes larger strains beneath the slab, which could help form stronger (100)[001] textures sharply contrasting with nearly untextured bridgmanite below the viscosity jump^47^. Additional mechanisms such as imbricated convection associated with slab-plume interactions in the mid-mantle^75^, or highly-time dependent flow during slab buckling^76^, provides another pathway for large strain accumulation assuming a constant transition stress (i.e., threshold at which dislocation and diffusion creep have identical viscosities)^77^, thereby increasing the sharpness of the discontinuity. Finally, a composite CPO and SPO-induced fabric in the mid-mantle could also likely increase the impedance contrast associated with the anisotropic discontinuity^28,47,74^.

Our models can also explain radial anisotropy in subduction zones, building on earlier single-fabric models by allowing strain accommodation to respond dynamically to mantle flow. Although topotactical relationships during phase transitions in the olivine system^78^ could, in principle, influence bridgmanite CPO in the surrounding mantle, such effects would remain insignificant upon crossing the 660 km boundary. In the upper MTZ, the olivine-wadsleyite and wadsleyite-ringwoodite transitions act as textural “filters" where randomly oriented grains may overgrow and weaken a pre-existing texture inherited from the parent phase^79,80^. And with the lower transition zone being generally isotropic^81,82^, slab-entrained aggregates should therefore acquire near-random textures by the time they enter the lower mantle, rendering their prior deformation history negligible. Within the slab, metastable olivine may still modulate anisotropy indirectly by altering slab dynamics. Its persistence enhances down-dip compression and positive buoyancy above the ~ 660 km boundary, promoting shallow slab stagnation^83,84^. Because metastable olivine is expected mainly in old, thick lithosphere^84^, such sites may be excluded in the search for mid-mantle reflectors associated with anisotropic discontinuities.

Subduction behavior is typically classified into three modes: (1) penetration, (2) stagnation near 660 km, and (3) trapping near 1000 km^19^. The existence of mode (3), however, remains debated as the flattened slab morphology in the present time might be ascribed to its previous resistance to impinge the 660-km boundary^20,85^. Prior geodynamic studies have nonetheless examined trapped-slab scenarios, showing sufficient CPO evolution despite increased resistance to deformation due to the imposed mid-mantle viscosity jump^28,47^. Although we do not model the trapped-slab case here, its exclusion does not undermine the general implications of our results. We expect that the same mechanism responsible for generating an anisotropic discontinuity would remain active under such conditions, and, as discussed earlier, a viscosity increase would likely sharpen the resulting anisotropic discontinuity.

Apart from Western Java, several subduction zones that have interacted with the mid-mantle are plausible candidates for generating large-scale anisotropy, including Cocos, Northern Peru, and Kamchatka^27^. These systems commonly exhibit slab flattening^19^, and in our simulations, flattened geometries distribute strain over broader regions, producing laterally extensive anisotropic discontinuities that could contribute to the observed variability in mid-mantle reflectors beneath subduction zones^4,6^. In contrast, settings characterized by vertically penetrating slabs, such as the Marianas^19,20^, are expected to generate narrower and slightly dipping discontinuities that border the slab tip. Although dipping chemical reservoirs have been proposed for reflectors observed in this region^11^, we suggest an anisotropic discontinuity offers an equally plausible and dynamically consistent explanation.

The 1000-km discontinuity has been hypothesized to represent a viscosity “hill” that facilitates the flattening of slabs at mid-mantle depths^86^ which would explain the ubiquity of reflectors in subduction zones^5,6^. Since the most abundant lower-mantle phases are not known to undergo phase transformation at mid-mantle depths, it has been suggested that variations in the grain size of bridgmanite-enriched ancient mantle structures account for this viscosity increase^23,24^. In practice, such structures are challenging to image since primordial bridgmanite exhibits velocities that are only marginally different to a pyrolitic mantle; not to mention the trade-offs associated with their thermo-elastic properties with temperature^87,88^. Alternatively, there is experimental evidence that the viscosity of a periclase-enriched region at lower mantle conditions is two orders of magnitude larger than the surrounding mantle, which may cause slabs to flatten and settle at ~ 1000 km^22^. Constraining such structures in high-stress regions, however, remains elusive^86^. Another explanation could be the inefficient phase transformation (i.e., slow diffusion suppresses the dissolution of a phase at equilibrium *P*-*T* conditions) of pyroxene to majoritic garnet, which causes thicker and colder slabs to be more vulnerable to stagnation at mid-mantle depths^89^.

**Fig. 6:**
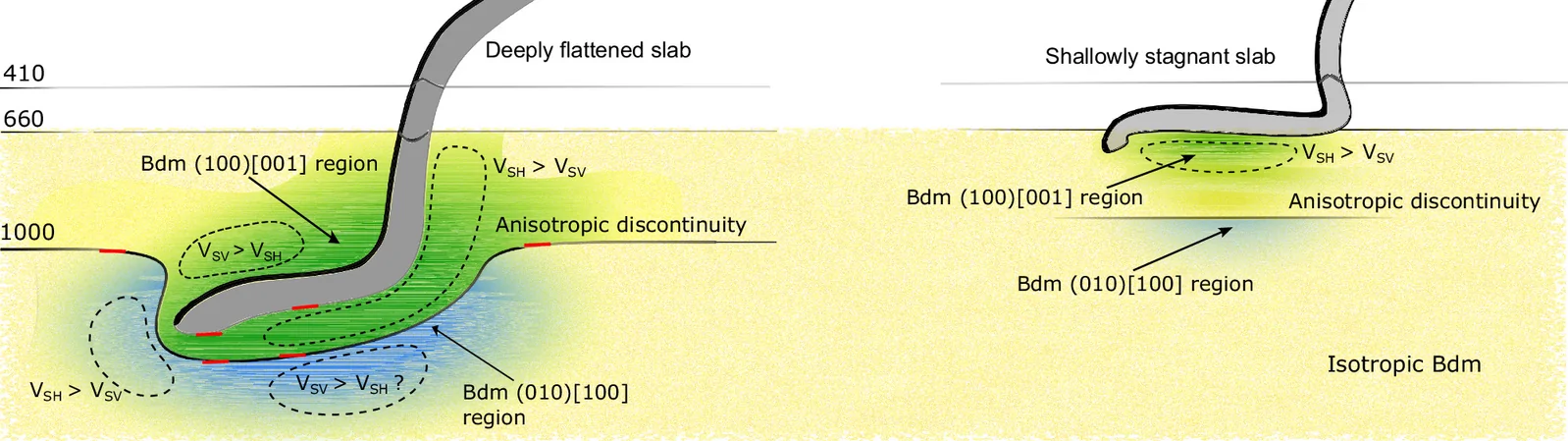
Mid-mantle discontinuities in subduction zones interpreted as a deformation-induced seismic feature. Schematic diagram illustrating the distribution of the anisotropic discontinuity and the general pattern of radial anisotropy—in terms of the relative strength between *V*_*SH*_ and *V*_*SV*_—induced by two subduction styles: deep flattening near 1000 km depth and shallow stagnation near the 660-km discontinuity. The entrained mantle surrounding slabs generally exhibits larger accumulated strains, and hence, the clustering of large-scale anisotropy around them; whilst the rest of the mantle remains isotropic (in yellow). A deeply flattened slab (e.g., Western Java subduction zone) results in a deeper distribution of anisotropy. Bdm refers to bridgmanite for brevity. Above 1000 km, crystallographic preferred orientation (CPO) evolution is primarily influenced by (100)[001] Bdm (in green). Slip transition occurs at 1000 km, and past this depth, (010)[100] Bdm now controls the depth distribution of anisotropy (in blue). This creates a deformation-induced anisotropic discontinuity that deepens in the presence of a slab. Red streaks, that perhaps could be more extensive, denote the potential location of mid-mantle reflectors in the underside of the anisotropic discontinuity. In the case of a shallowly stagnant slab (e.g., Honshu subduction zone), the anisotropic discontinuity persists at 1000 km, but appears faint due to weak deformation.

Our models based on a *P*-dependent bridgmanite fabric suggest that mid-mantle discontinuities near subduction zones may originate from deformation-induced seismic anisotropy, rather than from thermal and/or compositional heterogeneities (for our rendition, see Fig. 6). Moreover, our models also help reconcile anisotropy observations in the uppermost lower mantle with experimental data on bridgmanite deformation. Together, these results expand the range of mechanisms that may account for these seismic observations, thus, highlighting the role of texture evolution in shaping Earth’s deep structure. We stress, however, that the anisotropic discontinuity mechanism is unlikely the sole cause of these observations, but instead offers a complementary mechanism to existing thermo-chemical interpretations.

Looking ahead, coupling anisotropic viscosity feedback and depth-dependent bridgmanite deformation may further reveal how deep mantle fabrics influence slab behavior—whether they penetrate deeper or stagnate in the upper lower mantle. Similar to how olivine CPO in the asthenosphere facilitates plate motion through reduced viscosity^90^, we propose that the CPO evolution of bridgmanite could exert a comparable control on slab dynamics at depth.

## Methods

### Thermo-chemical modeling of intraoceanic subduction

The geodynamic modeling scheme is adapted from previous work^52^, with some modifications in the model parameters to also allow for the slab to penetrate the 660-km discontinuity. Here, we construct 2D subduction models using the open source geodynamic software ASPECT^91–93^, whose framework is based on a finite element model with adaptive mesh refinements and a variety of non-linear rheologies included as modules. The domain spans 6000 × 2000 km with spatial resolutions that vary adaptively from 5 × 5 km close to the slab and along the discontinuities in the MTZ up to 325 × 325 km in zones of quiescence (i.e., very far from the slab). The plates follow a half-space cooling model^94^ with a surface adiabatic temperature of 1573 K. Below the presumed lithosphere-asthenosphere boundary depth, a 0.53 K ⋅ km^−1^ linear gradient is imposed. Slab penetration or stagnation initiates depending on the chosen values of the parameters for the latent heating mechanism^95^, as well as the value of the imposed viscosity jump at 660 km that is harmonically averaged with the viscoplastic rheology^52^. Phase transition Clapeyron slopes, and viscoplastic rheology parameter values are indicated in Supplementary Tables 1 and 2.

### Depth-dependent deformation and anisotropy in bridgmanite

A previous work conducted a series of laser-heated diamond anvil cell experiments to deform polycrystalline pyrolite at in situ lower-mantle pressures and temperatures^43^. While no apparent textures were observed in davemaoite and ferropericlase under lower mantle conditions, three regimes are observed in bridgmanite deformation: (1) strong [001] orientations at 660 km depth conditions, induced by preferential growth of bridgmanite under stress, (2) weak re-orientation towards [100] at pressures below 50 GPa, corresponding to the depth range 660–1250 km, (3) followed by a smooth transition towards [010] with minor preferential alignments at [001] past 50 GPa. At increasing pressures, strong [010] textures remain. Full details about the experiment and interpretation of the experimental results have been documented in ref. ^43^.

Visco-plastic self-consistent^48^ (VPSC) modeling of the experimental data of ref. ^43^ leads to the following result: below 50 GPa, primary activity is observed in (100)[001] with secondary activities at (100)[010], (010)[001], and (001)[010] that are consistent with the literature ^39,96^. Above 50 GPa, *P*-induced slip transitions now occur with primary and secondary activities at (010)[100] and (010)[001], respectively (Fig. 1), in agreement with Peierls-Nabarro predictions of critically-resolved shear stresses (CRSS) in bridgmanite^45^ (Supplementary Fig. 8). VPSC simulation of *P*-dependent bridgmanite under simple shear is also shown in Supplementary Fig. 9. We subsequently fit a linear model to the relative CRSS of each active slip system in bridgmanite as a function of pressure up to 50 GPa, beyond which the CRSS values remain constant (Fig. 1c and Supplementary Table 3).

### Lower-mantle fabric modeling

Following the method of ref. ^52^, we model *P*-induced phase transformations to determine the depth extent of MTZ boundaries for appropriate mineral assignments when computing CPO. To do so, we employ Perple_X ^97,98^ to determine the mineral equilibrium assemblage of pyrolite at *P*-*T* conditions relevant to the mantle. Arbitrary density crossover associated with the phase transition boundary at 660 km is computed using the latest thermodynamic database of ref. ^99^. For a pyrolitic composition, the density *ρ* crossover at the 660 km boundary is *ρ*_660_ = 4170 kg ⋅ m^−3^.

As we are only interested in lower-mantle anisotropy, we only perform fabric calculations past *ρ*_660_ = 4170 kg ⋅ m^−3^. Aggregates are treated as Lagrangian tracers that are advected forward in time using the flow velocity computed from ASPECT. At each step along the pathline followed by the tracer, we calculate the CPO of 3000 grains with a modified version of the VPSC model that incorporates *P*-induced slip transitions^100^. Here, *P*-dependence is established by computing the local shear rate $\dot{\gamma }$ in a slip system *s* of a grain using the linear relationship between the relative CRSS ${\tau }_{0}^{s}$ and *P* (Supplementary Table 3):

1 $${\dot{\gamma }}^{s}=\dot{{\gamma }_{0}}{\left[\dfrac{{\tau }_{r}^{s}}{{\tau }_{0}^{s}(p)}\right]}^{{n}^{s}},$$

where $\dot{{\gamma }_{0}}$ is the reference shear rate, ${\tau }_{r}^{s}$ is the resolved shear stress, and *n* is the stress exponent in slip system *s*. For simplicity, we set a constant value *n* = 3 for all slip systems. Under our modeling conditions, variations in *n* would have a negligible impact on the resulting conclusions. We perform CPO calculations without the need for strain partitioning (i.e., full velocity gradient tensor is used to deform the aggregate), and only for the bridgmanite phase as it solely exhibits significant intrinsic visco-elastic anisotropy in a pyrolitic material at upper lower-mantle depths^43,101^. Random textures are, henceforth, imposed on davemaoite and ferropericlase. Crystallographic orientations are initially random-oriented in the background mantle. Inside the slab, we apply the orientated growth mechanism of ref. ^42^ for bridgmanite. Each time pathlines cross the 660 km boundary, textures of aggregates are re-randomized.

Polycrystal elastic tensors are computed based on a two-phase aggregate composed of 80% bridgmanite and 20% ferropericlase using a Voigt-Reuss-Hill averaging technique^102^. Bridgmanite single crystal elastic constants are computed for a given *P* and *T* using a lookup table produced from first-principles calculations of thermoelasticity based on the Wu-Wentzcovitch semi-analytical method^103^. Meanwhile, that of ferropericlase are computed using the *P*-*T* derivatives provided by ref. ^104^. Finally, given the full elastic tensors, radial anisotropy is computed at each point in space using the method of ref. ^105^. We also express our results in terms of the anisotropy index, a scalar quantity that measures the total amount of anisotropy present in the elastic tensor **c**. The anisotropy index *α* is defined as:

2 $$\alpha=\sqrt{\dfrac{{\sum}_{ij}{({c}_{ij}-{c}_{ij}^{0})}^{2}}{{\sum}_{ij}{({c}_{ij}^{0})}^{2}}},$$

where **c**^0^ is the isotropic part of the elastic tensor extracted using the method of ref. ^106^. We quantify the sharpness of the anisotropic discontinuity using a fixed threshold, defined as the vertical half-width over which *α* recovers from its local minimum, ${\alpha }_{\min }$ to a value corresponding to a 20% anisotropy index contrast: $2(\alpha -{\alpha }_{\min })/(\alpha+{\alpha }_{\min })$.

In the models presented here, the anisotropic discontinuity appears as a gradational change in seismic anisotropy over a depth interval, which mainly results from gradual shifts in slip system activities introduced via a linear dependence of bridgmanite CRSS on pressure. The resolution of the discontinuity, however, is also limited by the number of Lagrangian tracer aggregates sampling the discontinuity. In future work, the sharpness of the anisotropic discontinuity could be better constrained by increasing the number of tracers that make up the elastic medium, although this would require substantially greater computational resources. Because tracer density is also limited by the spatial resolution of the mantle flow model, resolving sharper discontinuities would additionally require a finer numerical mesh. Under such conditions, sharper anisotropic contrasts could emerge even with smooth pressure-induced slip transitions in bridgmanite.

### Shear wave splitting calculations

We calculate the effective shear wave splitting (SWS) parameters for a stack of horizontal layers (each defined by a full elastic tensor **c** with a 25-km spacing) that samples the subslab mantle between 660 and 1200 km (dashed vertical line in Fig. 5a: left panel) for all possible ray propagation directions. We do not include olivine anisotropy above 660 km depth in these calculations. Our modeling is thus analogous to source-side SWS analyses where receiver-side upper-mantle anisotropy is corrected or characterized to be effectively isotropic using *KS (where * can be P or S waves) splitting^66,67^. The chosen depth profile best captures the layer of positive radial anisotropy (*V*_*SH*_ > *V*_*SV*_) in the subslab mantle. Furthermore, it ensures that there is no interference with the intraslab anisotropy resulting from the transformation textures since the slab remains above the 660-km discontinuity at this location. To compare our predictions with published source-side SWS measurements underneath Western Java, we use the epicentral distance range over which source-side SWS using direct S waves is commonly measured (50°–90°). For each distance, we calculate the theoretical slowness of the direct S phase using the TauP Toolkit^107^ and PREM^108^, and then convert these values into incidence angles for the horizontal layers. The incidence angles and the azimuths relative to the model *x*-axis define the propagation directions through the representative subslab anisotropy profile. Using the Matlab Seismic Anisotropy Toolbox^109^ (MSAT), we first compute the splitting parameters of each layer and then evaluate the effective splitting operator following the method of ref. ^110^ for each azimuth and incidence angle. Finally, we adapt the work of ref. ^55^ to visualize the effective splitting as a function of distance and azimuth using a pole-figure representation.

### Anisotropic reflection calculations

We select three points along the anisotropic discontinuity where the degree of natural strain exceeds *E*_0_ > 0.5 ^28^, namely Locations A, B, and C in Fig. 2a, to ensure strong contrasts in anisotropy. To ensure that an anisotropic discontinuity is solely responsible for the generation of reflection coefficients, we remove the effect of variations in the isotropic velocities with pressure *P* and temperature *T* when we choose a top and bottom layer for calculating reflections. This is accomplished by selecting a node in the elastic medium **c**(*x*, *z*) where the anisotropic discontinuity interface is presumed to be located. The criteria for selecting such node is based on a grid search method with depth, where the gradient of the anisotropic discontinuity with depth is zero. Since the spatial resolution of **c**(*x*, *z*) is chosen ad-hoc, and hence finite, the node closest to the depth at which the local minima in the anisotropy index is found (i.e., $\frac{d}{dz}\alpha=0$) is chosen as the anisotropic interface. The elastic tensor corresponding to this interface is called **c**_mid_, whereby it is treated as a local first-order discontinuity between the top and bottom layers. Then, using the elastic decomposition method of ref. ^106^, the isotropic tensors of the top and bottom layers, which are chosen to be the nodes that are vertically adjacent to **c**_mid_ are removed, and in turn replaced with that of the middle node (i.e., the interface). The densities of the top and bottom layers are also replaced with that of the middle node (Supplementary Fig. 10).

We compute reflection coefficients at these interfaces using the REFVEC subroutine of the anisotropic ray tracer package ATRAK^111^ for a range of azimuths and incidence angles relative to the model *x* direction. Assuming a horizontal interface, we treat the problem as S^*d*^S reflections in the underside of the anisotropic discontinuity, where *d* pertains to the depth at which reflection occurs. With this, the bottom layer effectively becomes the incident layer within REFVEC. Locations A, B, and C are at depths *d* = 1150, 1400, 1250 km, respectively. Mid-mantle reflections typically occur at epicentral distances ~ 90°–160°^6,112^. To account for this, we use the TauP Toolkit^107^ to convert distances to incidence angles, with each conversion performed using a modified PREM^108^ that includes a discontinuity imposed at *d*. The resulting reflection coefficients thus represent the behavior of S^1150^S, S^1400^S, and S^1250^S phases in the vicinity of the anisotropic discontinuity. We also show qSV-qSH reflection coefficients owing to the cross coupling of SV and SH motions in the incident anisotropic layer^113^ (Supplementary Fig. 3b).

### Full waveform modeling and array analysis

We construct the 3D anisotropy model by extending the 2D anisotropy model in the along-strike direction such that the 2D structure is replicated uniformly along strike. The background model is isotropic PREM^108^. We consider two test cases: (1) fully anisotropic mantle model. Note that because the numerical resolution in fabric modeling is constrained by the limited number of Lagrangian tracers, the modeled discontinuity appears smoother. For the waveform simulations, we represent the transition as a sharper interface by assigning untextured bridgmanite at the discontinuity depth. To ensure that the seismic waves are perturbed solely by the anisotropic structure, we apply the elastic decomposition technique of Browaeys and Chevrot (2004)^106^, and replace the isotropic part of our 3D mantle model with that of PREM. (2) Isotropic mantle model. In this test case, we consider isotropic perturbations associated with the slab by modeling its velocity structure using a harzburgite composition^114^. *P*-*T* dependent seismic velocities and density of the slab are modeled using Perple_X (Supplementary Fig. 11)^97–99^. The resulting model is then transformed from Cartesian coordinates [*x*, *y*, *z*] to geographic coordinates [lat, lon, *z*] using a linear mapping before meshing.

We compute synthetic seismograms at the global scale using the SEM code AxiSEM3D^61^. The 3D subduction model is centered at [0, 0] (in [lat, lon] coordinates) with the slab dipping East-West (E-W), and the trench axis oriented North-South. We design a square seismic array centered at [0, 52°] spanning 20° laterally. The array is composed of 3000 stations with variable interstation spacing capped at a minimum of 11 km^62^. The source is a pure left-lateral strike-slip mechanism whose nodal plane orients E-W, located at [0, −58°] and a depth of 10 km. We use a Gaussian source-time function with a half-duration of 3 s. Simulations are performed at a minimum period of 4 s with a record length of one hour.

Because most ray paths lie close to the equator, errors from the axis-symmetric approximation are contained^61^. In addition, wavefield scanning shows that fewer than 400 Fourier coefficients are sufficient in both the in-plane and off-plane directions (Supplementary Fig. 12). We therefore use an expansion order of 400 to further suppress spurious signals.

For the array analysis, we define a circular sub-array within the main array with an aperture radius of 2.5° (~140 stations), measured from a reference receiver at the sub-array center. Processing of the noise-free synthetic waveforms is performed on the transverse component using ObsPy^115^. As a pre-processing step, we apply a second-order zero-phase Butterworth bandpass filter between 4 and 11 s. To test the detectability of the anisotropic discontinuity using SS precursors, we first apply 4th-root stacking^62^ to enhance their amplitudes, and subsequently compute slowness vespagrams^62^ between the Sdiff and the main SS phase time window.

## Supplementary information

Supplementary Information

Transparent Peer Review file

## Data Availability

The anisotropic tomography model shown Figs. 4b and 5b and Supplementary Fig. 5b are publicly available via the IRIS Earth Model Collaboration project (https://doi.org/10.17611/DP/EMC.1 and https://ds.iris.edu/ds/products/emc-sglobe-rani/). The model output and the synthetic data that support the findings of this work are available in Zenodo with the identifier https://doi.org/10.5281/zenodo.20514233^116^.

## Code availability

Subduction modeling was carried out using the open software ASPECT^91–93^ and can be freely accessed: https://zenodo.org/records/8200213. Mantle fabric calculations were performed using VPSC^48^, available at https://github.com/lanl/vpsc_code. Full waveform modeling was implemented using the open software AxiSEM3D^61^ (https://github.com/AxiSEMunity/AxiSEM3D), and processing of synthetic waveforms was completed in Obspy^115^. Decomposition, processing of elastic tensors, and shear wave splitting calculations were performed using MSAT^109^ (https://www1.gly.bris.ac.uk/MSAT/). The anisotropic reflection calculation code REFVEC—which is a subroutine of the commercial ray-tracer package ATRAK^111^—is subject to licensing restrictions and is not publicly available. For this, the reader may contact JW through J.Wookey@bristol.ac.uk.

## References

[1] Shearer, P. M. & Flanagan, M. P. Seismic velocity and density jumps across the 410-and 660-kilometer discontinuities. *Science* **285**, 1545–1548 (1999). 10.1126/science.285.5433.1545 10477514

[2] Deuss, A. & Woodhouse, J. Seismic observations of splitting of the mid-transition zone discontinuity in Earth’s mantle. *Science* **294**, 354–357 (2001). 10.1126/science.1063524 11598296

[3] Jeanloz, R. & Thompson, A. B. Phase transitions and mantle discontinuities. *Rev. Geophys.* **21**, 51–74 (1983).

[4] Deuss, A. & Woodhouse, J. H. A systematic search for mantle discontinuities using ss-precursors. *Geophys. Res. Lett.* **29**, 90–1 (2002).

[5] Deuss, A. Global observations of mantle discontinuities using SS and PP precursors. *Surv. Geophys.* **30**, 301–326 (2009).

[6] Waszek, L., Schmerr, N. C. & Ballmer, M. D. Global observations of reflectors in the mid-mantle with implications for mantle structure and dynamics. *Nat. Commun.* **9**, 385 (2018). 10.1038/s41467-017-02709-4 PMC5786065 29374158

[7] Yuan, Y., Magali, J., Deng, X., Sun, D. & Thomas, C. Mantle discontinuities and reflectors beneath the Arctic Ocean and Aleutian-Alaska subduction zone: evidence for MORB crust at the top of the lower mantle. *Earth Planet. Sci. Lett.* **652**, 119199 (2025).

[8] Yuan, Y. et al. Revealing the nature of mid-mantle discontinuities through global interrogation of SS precursors versus P-to-S conversions. *Earth Planet. Sci. Lett.* **689**, 120074 (2026).

[9] Vinnik, L., Kato, M. & Kawakatsu, H. Search for seismic discontinuities in the lower mantle. *Geophys. J. Int.* **147**, 41–56 (2001).

[10] Kaneshima, S. Seismic scatterers at the shallowest lower mantle beneath subducted slabs. *Earth Planet. Sci. Lett.* **286**, 304–315 (2009).

[11] Niu, F., Kawakatsu, H. & Fukao, Y. Seismic evidence for a chemical heterogeneity in the midmantle: a strong and slightly dipping seismic reflector beneath the Mariana subduction zone. *J. Geophys. Res.: Solid Earth* **108**, 2419 (2003).

[12] Haugland, S. M., Ritsema, J., Kaneshima, S. & Thorne, M. S. Estimate of the rigidity of eclogite in the lower mantle from waveform modeling of broadband S-to-P wave conversions. *Geophys. Res. Lett.* **44**, 11–778 (2017).

[13] Kaneshima, S. Seismic scatterers in the lower mantle near subduction zones. *Geophys. J. Int.* **219**, S2–S20 (2019).

[14] Yuan, Y., Sun, D., Leng, W. & Wu, Z. Southeastward dipping mid-mantle heterogeneities beneath the Sea of Okhotsk. *Earth Planet. Sci. Lett.* **573**, 117151 (2021).

[15] Wang, W. et al. Velocity and density characteristics of subducted oceanic crust and the origin of lower-mantle heterogeneities. *Nat. Commun.* **11**, 64 (2020). 10.1038/s41467-019-13720-2 PMC6946644 31911578

[16] Yu, Y. et al. Unraveling the complex features of the seismic scatterers in the mid-lower mantle through phase transition of (al, h)-bearing stishovite. *Geophys. Res. Lett.* **52**, e2024GL114146 (2025).

[17] Courtier, A. M. & Revenaugh, J. Slabs and shear wave reflectors in the midmantle. *J. Geophys. Res.: Solid Earth* **113**, B08312 (2008).

[18] Durand, S., Debayle, E., Ricard, Y., Zaroli, C. & Lambotte, S. Confirmation of a change in the global shear velocity pattern at around 1000 km depth. *Geophys. J. Int.* **211**, 1628–1639 (2017).

[19] Fukao, Y. & Obayashi, M. Subducted slabs stagnant above, penetrating through, and trapped below the 660 km discontinuity. *J. Geophys. Res.: Solid Earth* **118**, 5920–5938 (2013).

[20] Goes, S., Agrusta, R., van Hunen, J. & Garel, F. Subduction-transition zone interaction: a review. *Geosphere* **13**, 644–664 (2017).

[21] French, S. W. & Romanowicz, B. Broad plumes rooted at the base of the Earth’s mantle beneath major hotspots. *Nature* **525**, 95–99 (2015). 10.1038/nature14876 26333468

[22] Marquardt, H. & Miyagi, L. Slab stagnation in the shallow lower mantle linked to an increase in mantle viscosity. *Nat. Geosci.* **8**, 311–314 (2015).

[23] Ballmer, M. D., Houser, C., Hernlund, J. W., Wentzcovitch, R. M. & Hirose, K. Persistence of strong silica-enriched domains in the Earth’s lower mantle. *Nat. Geosci.* **10**, 236–240 (2017).

[24] Fei, H. et al. Variation in bridgmanite grain size accounts for the mid-mantle viscosity jump. *Nature* **620**, 794–799 (2023). 10.1038/s41586-023-06215-0 PMC10447242 37407826

[25] Long, M. D. & Becker, T. W. Mantle dynamics and seismic anisotropy. *Earth Planet. Sci. Lett.* **297**, 341–354 (2010).

[26] French, S. & Romanowicz, B. A. Whole-mantle radially anisotropic shear velocity structure from spectral-element waveform tomography. *Geophys. J. Int.* **199**, 1303–1327 (2014).

[27] Chang, S.-J., Ferreira, A. M., Ritsema, J., van Heijst, H. J. & Woodhouse, J. H. Global radially anisotropic mantle structure from multiple datasets: a review, current challenges, and outlook. *Tectonophysics* **617**, 1–19 (2014).

[28] Ferreira, A. M., Faccenda, M., Sturgeon, W., Chang, S.-J. & Schardong, L. Ubiquitous lower-mantle anisotropy beneath subduction zones. *Nat. Geosci.* **12**, 301–306 (2019).

[29] Karato, S. -i On the Lehmann discontinuity. *Geophys. Res. Lett.* **19**, 2255–2258 (1992).

[30] Deuss, A. & Woodhouse, J. H. The nature of the Lehmann discontinuity from its seismological Clapeyron slopes. *Earth Planet. Sci. Lett.* **225**, 295–304 (2004).

[31] Rychert, C. A., Harmon, N. & Schmerr, N. Synthetic waveform modelling of ss precursors from anisotropic upper-mantle discontinuities. *Geophys. J. Int.* **196**, 1694–1705 (2014).

[32] Pisconti, A., Creasy, N., Wookey, J., Long, M. D. & Thomas, C. Mineralogy, fabric and deformation domains in D” across the southwestern border of the African LLSVP. *Geophys. J. Int.* **232**, 705–724 (2023).

[33] Long, M. D. Constraints on subduction geodynamics from seismic anisotropy. *Rev. Geophys.* **51**, 76–112 (2013).

[34] Nowacki, A., Kendall, J.-M., Wookey, J. & Pemberton, A. Mid-mantle anisotropy in subduction zones and deep water transport. *Geochem. Geophys. Geosyst.* **16**, 764–784 (2015).

[35] Walpole, J., Wookey, J., Kendall, J.-M. & Masters, T.-G. Seismic anisotropy and mantle flow below subducting slabs. *Earth Planet. Sci. Lett.* **465**, 155–167 (2017).

[36] De Wit, R. & Trampert, J. Robust constraints on average radial lower mantle anisotropy and consequences for composition and texture. *Earth Planet. Sci. Lett.* **429**, 101–109 (2015).

[37] Ringwood, A. A model for the upper mantle: 2. *J. Geophys. Res.* **67**, 4473–4478 (1962).

[38] Miyagi, L. & Wenk, H.-R. Texture development and slip systems in bridgmanite and bridgmanite+ ferropericlase aggregates. *Phys. Chem. Miner.* **43**, 597–613 (2016).

[39] Tsujino, N. et al. Mantle dynamics inferred from the crystallographic preferred orientation of bridgmanite. *Nature* **539**, 81–84 (2016). 10.1038/nature19777 27750277

[40] Karato, S.-I. & Li, P. Diffusion creep in perovskite: implications for the rheology of the lower mantle. *Science* **255**, 1238–1240 (1992). 10.1126/science.255.5049.1238 17816832

[41] McNamara, A. K., Karato, S.-I. & Van Keken, P. E. Localization of dislocation creep in the lower mantle: implications for the origin of seismic anisotropy. *Earth Planet. Sci. Lett.* **191**, 85–99 (2001).

[42] Gay, J. P. et al. Transformation microstructures in pyrolite under stress: implications for anisotropy in subducting slabs below the 660 km discontinuity. *Earth Planet. Sci. Lett.* **604**, 118015 (2023).

[43] Gay, J. P. et al. Depth dependent deformation and anisotropy of pyrolite in the Earth’s lower mantle. *Geophys. Res. Lett.* **51**, e2024GL109433 (2024).

[44] Hirel, P., Kraych, A., Carrez, P. & Cordier, P. Atomic core structure and mobility of [1 0 0](0 1 0) and [0 1 0](1 0 0) dislocations in MgSiO_3_ perovskite. *Acta Mater.* **79**, 117–125 (2014).

[45] Kraych, A., Carrez, P. & Cordier, P. On dislocation glide in MgSiO_3_ bridgmanite at high-pressure and high-temperature. *Earth Planet. Sci. Lett.* **452**, 60–68 (2016).

[46] Reali, R. et al. The role of diffusion-driven pure climb creep on the rheology of bridgmanite under lower mantle conditions. *Sci. Rep.* **9**, 2053 (2019). 10.1038/s41598-018-38449-8 PMC6376055 30765772

[47] Sturgeon, W., Ferreira, A. M., Faccenda, M., Chang, S.-J. & Schardong, L. On the origin of radial anisotropy near subducted slabs in the midmantle. *Geochem. Geophys. Geosyst.* **20**, 5105–5125 (2019).

[48] Lebensohn, R. A. & Tomé, C. A self-consistent anisotropic approach for the simulation of plastic deformation and texture development of polycrystals: application to zirconium alloys. *Acta Metall. Mater.* **41**, 2611–2624 (1993).

[49] Wenk, H.-R. & Tomé, C. N. Modeling dynamic recrystallization of olivine aggregates deformed in simple shear. *J. Geophys. Res.: Solid Earth* **104**, 25513–25527 (1999).

[50] Tommasi, A. Forward modeling of the development of seismic anisotropy in the upper mantle. *Earth Planet. Sci. Lett.* **160**, 1–13 (1998).

[51] Li, Z.-H., Di Leo, J. F. & Ribe, N. M. Subduction-induced mantle flow, finite strain, and seismic anisotropy: numerical modeling. *J. Geophys. Res.: Solid Earth* **119**, 5052–5076 (2014).

[52] Magali, J. K., Thomas, C., Ledoux, E. E., Capdeville, Y. & Merkel, S. On the influence of pressure, phase transitions, and water on large-scale seismic anisotropy underneath a subduction zone. *Geochem. Geophys. Geosyst.* **26**, e2024GC011827 (2025).

[53] Fryer, G. J. & Frazer, L. N. Seismic waves in stratified anisotropic media. *Geophys. J. Int.* **78**, 691–710 (1984).

[54] Saki, M., Thomas, C., Merkel, S. & Wookey, J. Detecting seismic anisotropy above the 410 km discontinuity using reflection coefficients of underside reflections. *Phys. Earth Planet. Inter.* **274**, 170–183 (2018).

[55] Pisconti, A., Thomas, C. & Wookey, J. Discriminating between causes of D” anisotropy using reflections and splitting measurements for a single path. *J. Geophys. Res.: Solid Earth* **124**, 4811–4830 (2019).

[56] Frazer, W. D. & Park, J. High-resolution mid-mantle imaging with multiple-taper ss-precursor estimates. *Geophys. J. Int.* **233**, 1356–1371 (2023).

[57] Fryer, G. J. & Frazer, L. N. Seismic waves in stratified anisotropic media-II. elastodynamic eigensolutions for some anisotropic systems. *Geophys. J. Int.* **91**, 73–101 (1987).

[58] Guest, W., Thomson, C. & Spencer, C. Anisotropic reflection and transmission calculations with application to a crustal seismic survey from the east Greenland shelf. *J. Geophys. Res.: Solid Earth* **98**, 14161–14184 (1993).

[59] Chapman, C. *Fundamentals of Seismic Wave Propagation* (Cambridge University Press, 2004).

[60] Booth, D. C. & Crampin, S. The anisotropic reflectivity technique: theory. *Geophys. J. Int.* **72**, 755–766 (1983).

[61] Leng, K., Nissen-Meyer, T., Van Driel, M., Hosseini, K. & Al-Attar, D. AxiSEM3D: broad-band seismic wavefields in 3-D global earth models with undulating discontinuities. *Geophys. J. Int.* **217**, 2125–2146 (2019).

[62] Rost, S. & Thomas, C. Array seismology: methods and applications. *Rev. Geophys.* **40**, 2–1 (2002).

[63] Chang, S.-J., Ferreira, A. M., Ritsema, J., van Heijst, H. J. & Woodhouse, J. H. Joint inversion for global isotropic and radially anisotropic mantle structure including crustal thickness perturbations. *J. Geophys. Res.: Solid Earth* **120**, 4278–4300 (2015).

[64] Faccenda, M. Mid mantle seismic anisotropy around subduction zones. *Phys. Earth Planet. Inter.* **227**, 1–19 (2014).

[65] Di Leo, J. et al. Mantle flow in regions of complex tectonics: insights from Indonesia. *Geochem. Geophys. Geosyst.* **13**, Q12008 (2012).

[66] Lynner, C. & Long, M. D. Sub-slab anisotropy beneath the Sumatra and circum-pacific subduction zones from source-side shear wave splitting observations. *Geochem. Geophys. Geosyst.* **15**, 2262–2281 (2014).

[67] Mohiuddin, A., Long, M. D. & Lynner, C. Mid-mantle seismic anisotropy beneath southwestern Pacific subduction systems and implications for mid-mantle deformation. *Phys. Earth Planet. Inter.* **245**, 1–14 (2015).

[68] Savage, M. Seismic anisotropy and mantle deformation: what have we learned from shear wave splitting?. *Rev. Geophys.* **37**, 65–106 (1999).

[69] Akaogi, M., Tanaka, A. & Ito, E. Garnet–ilmenite–perovskite transitions in the system Mg_4_Si_4_O_12_–Mg_3_Al_2_Si_3_O_12_ at high pressures and high temperatures: phase equilibria, calorimetry and implications for mantle structure. *Phys. Earth Planet. Inter.* **132**, 303–324 (2002).

[70] Ohtani, E. Water in the mantle. *Elements* **1**, 25–30 (2005).

[71] Shiraishi, R., Ohtani, E., Kanagawa, K., Shimojuku, A. & Zhao, D. Crystallographic preferred orientation of akimotoite and seismic anisotropy of Tonga slab. *Nature* **455**, 657–660 (2008). 10.1038/nature07301 18833278

[72] Rosa, A. D. et al. Shear wave anisotropy in textured phase D and constraints on deep water recycling in subduction zones. *Earth Planet. Sci. Lett.* **377**, 13–22 (2013).

[73] Wolf, J., Long, M. D., Leng, K. & Nissen-Meyer, T. Constraining deep mantle anisotropy with shear wave splitting measurements: challenges and new measurement strategies. *Geophys. J. Int.* **230**, 507–527 10.1093/gji/ggac055 (2022).

[74] Magali, J. et al. Quantifying intrinsic and extrinsic contributions to radial anisotropy in tomographic models. *J. Geophys. Res.: Solid Earth* **126**, e2021JB022322 (2021).

[75] Fletcher, M. & Wyman, D. A. Mantle plume–subduction zone interactions over the past 60 Ma. *Lithos* **233**, 162–173 (2015).

[76] McNamara, A. K., Van Keken, P. E. & Karato, S.-I. Development of anisotropic structure in the Earth’s lower mantle by solid-state convection. *Nature* **416**, 310–314 (2002). 10.1038/416310a 11907574

[77] McNamara, A. K., van Keken, P. E. & Karato, S.-I. Development of finite strain in the convecting lower mantle and its implications for seismic anisotropy. *J. Geophys. Res.: Solid Earth* **108**, 2230 (2003).

[78] Mainprice, D., Humbert, M. & Wagner, F. Phase transformations and inherited lattice preferred orientations: implications for seismic properties. *Tectonophysics* **180**, 213–228 (1990).

[79] Kerschhofer, L., Rubie, D., Sharp, T., McConnell, J. & Dupas-Bruzek, C. Kinetics of intracrystalline olivine–ringwoodite transformation. *Phys. Earth Planet. Inter.* **121**, 59–76 (2000).

[80] Smyth, J. R. et al. Olivine–wadsleyite–pyroxene topotaxy: evidence for coherent nucleation and diffusion-controlled growth at the 410-km discontinuity. *Phys. Earth Planet. Inter.* **200**, 85–91 (2012).

[81] Fouch, M. J. & Fischer, K. M. Mantle anisotropy beneath northwest Pacific subduction zones. *J. Geophys. Res.: Solid Earth* **101**, 15987–16002 (1996).

[82] Ohuchi, T., Fujino, K., Kawazoe, T. & Irifune, T. Crystallographic preferred orientation of wadsleyite and ringwoodite: effects of phase transformation and water on seismic anisotropy in the mantle transition zone. *Earth Planet. Sci. Lett.* **397**, 133–144 (2014).

[83] Pokornỳ, J., Čížková, H., Bina, C. R. & van den Berg, A. 2d stress rotation in the Tonga subduction region. *Earth Planet. Sci. Lett.* **621**, 118379 (2023).

[84] Schmeling, H., Monz, R. & Rubie, D. C. The influence of olivine metastability on the dynamics of subduction. *Earth Planet. Sci. Lett.* **165**, 55–66 (1999).

[85] Replumaz, A., Karason, H., van der Hilst, R. D., Besse, J. & Tapponnier, P. 4-d evolution of SE Asia’s mantle from geological reconstructions and seismic tomography. *Earth Planet. Sci. Lett.* **221**, 103–115 (2004).

[86] Rudolph, M. L., Lekić, V. & Lithgow-Bertelloni, C. Viscosity jump in Earth’s mid-mantle. *Science* **350**, 1349–1352 (2015). 10.1126/science.aad1929 26659053

[87] Gülcher, A. J. P., Ballmer, M. D. & Tackley, P. J. Coupled dynamics and evolution of primordial and recycled heterogeneity in Earth’s lower mantle. *Solid Earth* **12**, 2087–2107 (2021).

[88] Murakami, M., Khan, A., Sossi, P. A., Ballmer, M. D. & Saha, P. The composition of Earth’s lower mantle. *Annu. Rev. Earth Planet. Sci.* **52**, 605–638 (2024).

[89] King, S. D., Frost, D. J. & Rubie, D. C. Why cold slabs stagnate in the transition zone. *Geology* **43**, 231–234 (2015).

[90] Király, Á, Conrad, C. P. & Hansen, L. N. Evolving viscous anisotropy in the upper mantle and its geodynamic implications. *Geochem. Geophys. Geosyst.* **21**, e2020GC009159 (2020).

[91] Kronbichler, M., Heister, T. & Bangerth, W. High accuracy mantle convection simulation through modern numerical methods. *Geophys. J. Int.* **191**, 12–29 (2012).

[92] Heister, T., Dannberg, J., Gassmöller, R. & Bangerth, W. High accuracy mantle convection simulation through modern numerical methods–ii: realistic models and problems. *Geophys. J. Int.* **210**, 833–851 (2017).

[93] Bangerth, W., Dannberg, J., Gassmoeller, R. & Heister, T. Aspect v2. 2.0. *Zenodo.* 10.5281/zenodo.3924604 (2020).

[94] Turcotte, D. L. & Schubert, G. *Geodynamics* (Cambridge University Press, 2002).

[95] Agrusta, R., Goes, S. & Van Hunen, J. Subducting-slab transition-zone interaction: stagnation, penetration and mode switches. *Earth Planet. Sci. Lett.* **464**, 10–23 (2017).

[96] Couper, S., Speziale, S., Marquardt, H., Liermann, H.-P. & Miyagi, L. Does heterogeneous strain act as a control on seismic anisotropy in Earth’s lower mantle? *Front. Earth Sci.* **8**, 540449 (2020).

[97] Connolly, J. A. Computation of phase equilibria by linear programming: a tool for geodynamic modeling and its application to subduction zone decarbonation. *Earth Planet. Sci. Lett.* **236**, 524–541 (2005).

[98] Connolly, J. The geodynamic equation of state: what and how. *Geochem. Geophys. Geosyst.* **10**, Q10014 (2009).

[99] Stixrude, L. & Lithgow-Bertelloni, C. Thermodynamics of mantle minerals–iii: the role of iron. *Geophys. J. Int.* **237**, 1699–1733 (2024).

[100] Magali, J.-K., Ledoux, E., Thomas, C., Capdeville, Y. & Merkel, S. Pressure-dependent large-scale seismic anisotropy induced by non-Newtonian mantle flow. *Geophys. J. Int.* **238**, 400–419 (2024).

[101] Tommasi, A., Mainprice, D., Cordier, P., Thoraval, C. & Couvy, H. Strain-induced seismic anisotropy of wadsleyite polycrystals and flow patterns in the mantle transition zone. *J. Geophys. Res.: Solid Earth* **109**, B12405 (2004).

[102] Mainprice, D. A fortran program to calculate seismic anisotropy from the lattice preferred orientation of minerals. *Comput. Geosci.* **16**, 385–393 (1990).

[103] Luo, C. et al. cij: a Python code for quasiharmonic thermoelasticity. *Comput. Phys. Commun.* **267**, 108067 (2021).

[104] Karki, B., Wentzcovitch, R. M., De Gironcoli, S. & Baroni, S. High-pressure lattice dynamics and thermoelasticity of MgO. *Phys. Rev. B* **61**, 8793 (2000).

[105] Montagner, J.-P. & Nataf, H.-C. A simple method for inverting the azimuthal anisotropy of surface waves. *J. Geophys. Res.: Solid Earth* **91**, 511–520 (1986).

[106] Browaeys, J. T. & Chevrot, S. Decomposition of the elastic tensor and geophysical applications. *Geophys. J. Int.* **159**, 667–678 (2004).

[107] Crotwell, H. P. et al. The taup toolkit: flexible seismic travel-time and ray-path utilities. *Seismol. Res. Lett.* **70**, 154–160 (1999).

[108] Dziewonski, A. M. & Anderson, D. L. Preliminary reference earth model. *Phys. Earth Planet. Inter.* **25**, 297–356 (1981).

[109] Walker, A. M. & Wookey, J. Msat-a new toolkit for the analysis of elastic and seismic anisotropy. *Comput. Geosci.* **49**, 81–90 (2012).

[110] Silver, P. G. & Savage, M. K. The interpretation of shear-wave splitting parameters in the presence of two anisotropic layers. *Geophys. J. Int.* **119**, 949–963 (1994).

[111] Guest, W. & Kendall, J. Modelling waveforms in anisotropic inhomogeneous media using ray and Maslov asymptotic theory: applications to exploration seismology. *Can. J. Explor. Geophys.* **29**, 78–92 (1993).

[112] Saki, M., Thomas, C. & Abreu, R. Detection and modelling of strong topography of mid-mantle structures beneath the North Atlantic. *Geophys. J. Int.* **229**, 219–234 (2022).

[113] Keith, C. M. & Crampin, S. Seismic body waves in anisotropic media: synthetic seismograms. *Geophys. J. Int.* **49**, 225–243 (1977).

[114] Irifune, T. & Ringwood, A. Phase transformations in a harzburgite composition to 26 gpa: implications for dynamical behaviour of the subducting slab. *Earth Planet. Sci. Lett.* **86**, 365–376 (1987).

[115] Beyreuther, M. et al. Obspy: a Python toolbox for seismology. *Seismol. Res. Lett.* **81**, 530–533 (2010).

[116] Magali, J. K. et al. Data supporting “mid-mantle discontinuities beneath subduction zones linked to depth-dependent deformation of bridgmanite” 10.5281/zenodo.20514233 (2026).42834069

