# Supplementary material for: Mid-mantle discontinuities beneath subduction zones linked to depth-dependent deformation of bridgmanite
Source: Nat Commun. 2026 Oct 5;17:10494. doi: 10.1038/s41467-026-78212-6 (PMC13639073; doi:10.1038/s41467-026-78212-6)
Supplement: Supplementary file 1 [file 41467_2026_78212_MOESM1_ESM.pdf]

Supplementary information

Mid-mantle discontinuities beneath subduction zones linked to depth-dependent deformation of bridgmanite

John Keith Magali[1,*], Sébastien Merkel[2], Angelo Pisconti[1], Jeffrey Gay[3], James Wookey[4], Christine Thomas[1,5]

[1]Institute of Geophysics, University of Münster, Correnssstr. 24, 48149 Münster, Germany

[2]Univ. Lille, CNRS, INRAE, Centrale Lille, UMR 8207 - UMET - Unité Matériaux et Transformations, F-59000 Lille, France

[3]Dept. of Geosciences, Princeton University, Princeton, NJ 08544, USA.

[4]School of Earth Sciences, University of Bristol, Wills Memorial Building, Queens Road, Bristol BS8 1RJ, UK

[5]Geological Survey of Denmark and Greenland, Copenhagen, Denmark

[*]Corresponding author: jmagali@uni-muenster.de

Contents of this file and brief descriptions

Supplementary Texts 1 to 2.

Supplementary Tables 1 to 3.

Supplementary Figures 1 to 13.

All figures shown here, except Supplementary Fig. 13, are referenced in the Main Text only. Below, we provide their brief descriptions.

Supplementary Figures 1 and 2: Calculated natural strain and anisotropic discontinuity for the shallowly stagnant slab model.

Supplementary Figures 3 and 4: Additional reflection coefficient calculations and waveform modeling results for the deeply flattened slab model.

Supplementary Figure 5: Predicted radial anisotropy for the shallowly stagnant slab model.

Supplementary Figure 6: Predicted and observed shear wave splitting delay times beneath Western Java.

Supplementary Figure 7: Predicted anisotropy assuming abrupt change in bridgmanite CRSS at 50 GPa (∼1270 km depth) for the deeply flattened slab model.

Supplementary Figures 8 and 9: VPSC modeling results for compression and simple shear.

Supplementary Figure 10: Visual aid for implementing first-order anisotropic reflection calculations.

Supplementary Figure 11: Seismic velocity model of a harzburgitic slab.

Supplementary Figure 12: Wavefield scanning results from AxiSEM3D.

Supplementary figure 13: Intraslab anisotropy modeling.

1 Rheological parameters in the free subduction model

The effective mantle viscosity η_{eff} is defined as[1]:

$$\eta_{\text{eff}}(z, P, T, ..) = \eta_0(z)\frac{\eta_{\text{vp}}(P, T, ..)}{\eta_{\min}}, \tag{1}$$

where $\eta_{\min}$ is the minimum viscosity capped at $\eta_{\min} = 1.0 \times 10^{20}$ Pa · s, and $\eta_0(z)$ and $\eta_{vp}(P, T, ...)$ are the depth-dependent[2] and viscosplastic terms[3,4], respectively. The specific formulations of η_0 and η_{vp} governing the rheology of intra-oceanic subduction in our model are detailed in ref.[5]. The values of the parameters are specified in Supplementary Tables 1 and 2.

2 Transformation textures as cause for intraslab anisotropy

Modeling intraslab anisotropy is still at its infancy, and so we assume that any hint of fossil fabric is completely overprinted by transformation textures during the ringwoodite-bridgmanite phase transition[6]. Due to negligible intraslab deformation, transformation textures only undergo rigid body rotation which would explain the 90° rotation of S-wave anisotropy around the $y-$axis when the slab orientation changes from vertical to horizontal and vice versa (Supplementary Fig. 13). There is no first-order comparison between our intraslab anisotropy model and tomography, mainly due to limited tomographic resolution; meanwhile, we anticipate that it is detectable by source-side shear wave splitting (SWS) when the direct S wave is oriented nearly parallel to the slab dip[7,8]. This is demonstrated in the bottom panels of Supplementary Fig. 13 showing V_S anisotropy pole figures. Here, source-side shear wave splitting measurements are expected to be: (1) both detectable in both updip and downdip directions at longer epicentral distances (i.e., steeper incidence angles) when the slab is oriented horizontally ($V_{SH} > V_{SV}$), (2) undetectable in both directions when the slab is vertical regardless of distance and azimuth ($V_{SV} > V_{SH}$), and (3) detectable only in the updip direction at the zone where the slab contorts in the NW-SE direction ($V_{SH} \sim V_{SV}$).

Due to the lack of SWS observations on deep intraslab anisotropy beneath Western Java, we

instead compare our intraslab anisotropy model with that of the Nazca slab, characterized by a comparable slab geometry[9]. Yet, our model cannot explain the along-strike intraslab anisotropy observed within the Nazca slab[7,8]. Although anisotropy resulting from the preferential alignment of serpentenized faults could lead to such observations[10], the fact that their stability remains inconclusive with increasing pressure[11], and that the length scale of these hydrous laminations remain thin makes them difficult to constrain by SWS measurements[7]. To reconcile the preservation of transformation textures within the slab as cause for the observed splitting[6 12], the most plausible scenario to exhibit trench-parallel anisotropy warrants along-strike buckling of the slab resulting from 3D flow.

Supplementary Table 1: Viscoplastic rheology parameters for both shallowly stagnant and deeply flattened slab models

Parameter	Symbol*	Subducting crust	Subducting lithopshere	Overriding plate	Background mantle	Unit
Density	ρ	3150	3350	3350	3350	$kg \cdot m^{-3}$
Thermal diffusivity	k	1.0×10^{-6}	1.0×10^{-6}	1.0×10^{-6}	1.0×10^{-6}	$m^2 \cdot s$
Heat capacity	C_p	1250	1250	1250	1250	$J \cdot K^{-1} \cdot kg^{-1}$
Dislocation creep						
Activation energy	E	0	5.4×10^5	4.3×10^5	4.3×10^5	$J \cdot mol^{-1}$
Activation volume	V	0	2.0×10^{-5}	1.85×10^{-5}	1.5×10^{-5}	$mol^3 \cdot mol^{-1}$
Prefactor	V	1.0×10^{-19}	2.4×10^{-16}	2.42×10^{-16}	3.91×10^{-15}	$Pa^{-n} \cdot s^{-1}$
Stress exponent	n	1.0	3.5	3.5	3.0	–
Diffusion creep						
Activation energy	E	0	3.0×10^5	2.4×10^5	2.4×10^5	$J \cdot mol^{-1}$
Activation volume	V	0	5.0×10^{-6}	5.0×10^{-6}	2.5×10^{-6}	$mol^3 \cdot mol^{-1}$
Prefactor	V	1.0×10^{-19}	6.08×10^{-14}	6.08×10^{-14}	3.74×10^{-14}	$Pa^{-n} \cdot s^{-1}$
Plastic yielding						
Cohesion	C	1.0×10^{15}	1.0×10^6	1.0×10^{15}	1.0×10^6	MPa
Internal friction angle	θ	0	20	0	20	°

*Chosen values are loosely constrained from[13]. To facilitate slab stagnation, we impose a viscosity jump by a factor of ~ 5 at 660 km in the depth-dependent model. Unless stated otherwise, all remaining parameters in ASPECT's viscoplastic material model are retained at their default settings.

Supplementary Table 2: Phase transition parameters in the mantle transition zone.

Parameter, deeply flattened/stagnant	410 km	520 km	660 km	Unit
Clapeyron slope	$2.5 \times 10^6/2.5 \times 10^6$	$0.5 \times 10^6/0.5 \times 10^6$	$-0.5 \times 10^6/-2.1 \times 10^6$	$Pa \cdot K^{-1}$
Density contrast*	370/370	130/130	320/320	$kg \cdot m^{-3}$
Phase transition width	10/10	5/5	5/10	km
Phase transition temperature	1750/1750	1850/1850	1900/1900	K

*Density contrasts are reminiscent of the pyrolite model.

Supplementary Table 3: First-order polynomial coefficients that fit the critically-resolved shear stresses (CRSS) τ_0 of bridgmanite as a function of pressure P according to the VPSC calculation of ref.[14]. The linear expression follows the form $\tau_0(P)_{\langle uvw\rangle\{hkl\}} = A_1 P + A_2$ where $\langle uvw\rangle\{hkl\}$ is a slip system. Note that τ_0 only refers to relative CRSS values in the VPSC calculations.

	(100)[010]	(100)[001]	(100)⟨011⟩	(010)[100]	(010)[001]	(010)⟨101⟩	(001)[100]	(001)[010]	(001)⟨110⟩	{111}⟨110⟩
A_1	0.0095	0.0143	0.0095	-0.01360	-0.0047	0.0045	-0.0095	0.0047	0.0	0.0
A_2	-0.0762	-0.3142	0.0238	0.7818	0.3381	0.1727	0.6762	0.5381	0.5	3.0

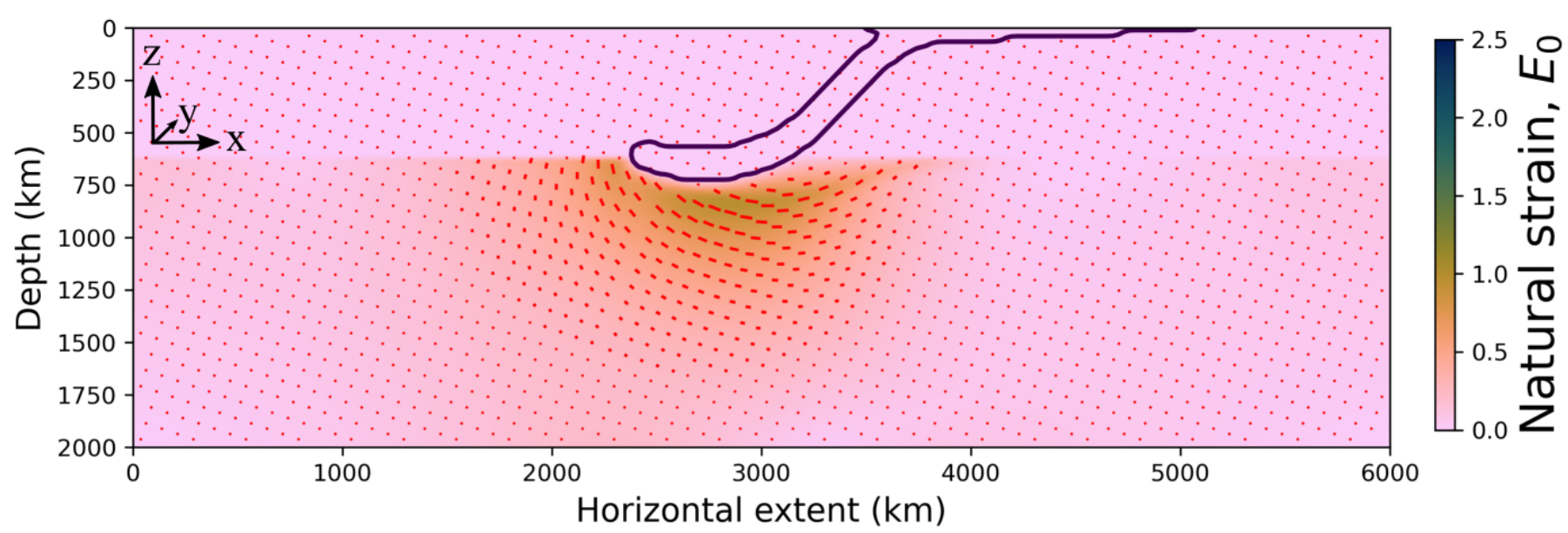

Supplementary Figure 1: **Natural strains in the lower mantle for the shallowly stagnant slab model.** Amount of accumulated deformation in the present time in terms of the finite strain ellipse (red arrow-less vectors) for a slab that is stagnating at the 660-km ringwoodite→bridgmanite phase transition boundary. The color map pertains to the natural strain E_0 defined as $E_0 = \frac{1}{2}\ln(\zeta_1/\zeta_3)$ where ζ_1 and ζ_3 are the long and short axes, respectively, of the finite strain ellipse.

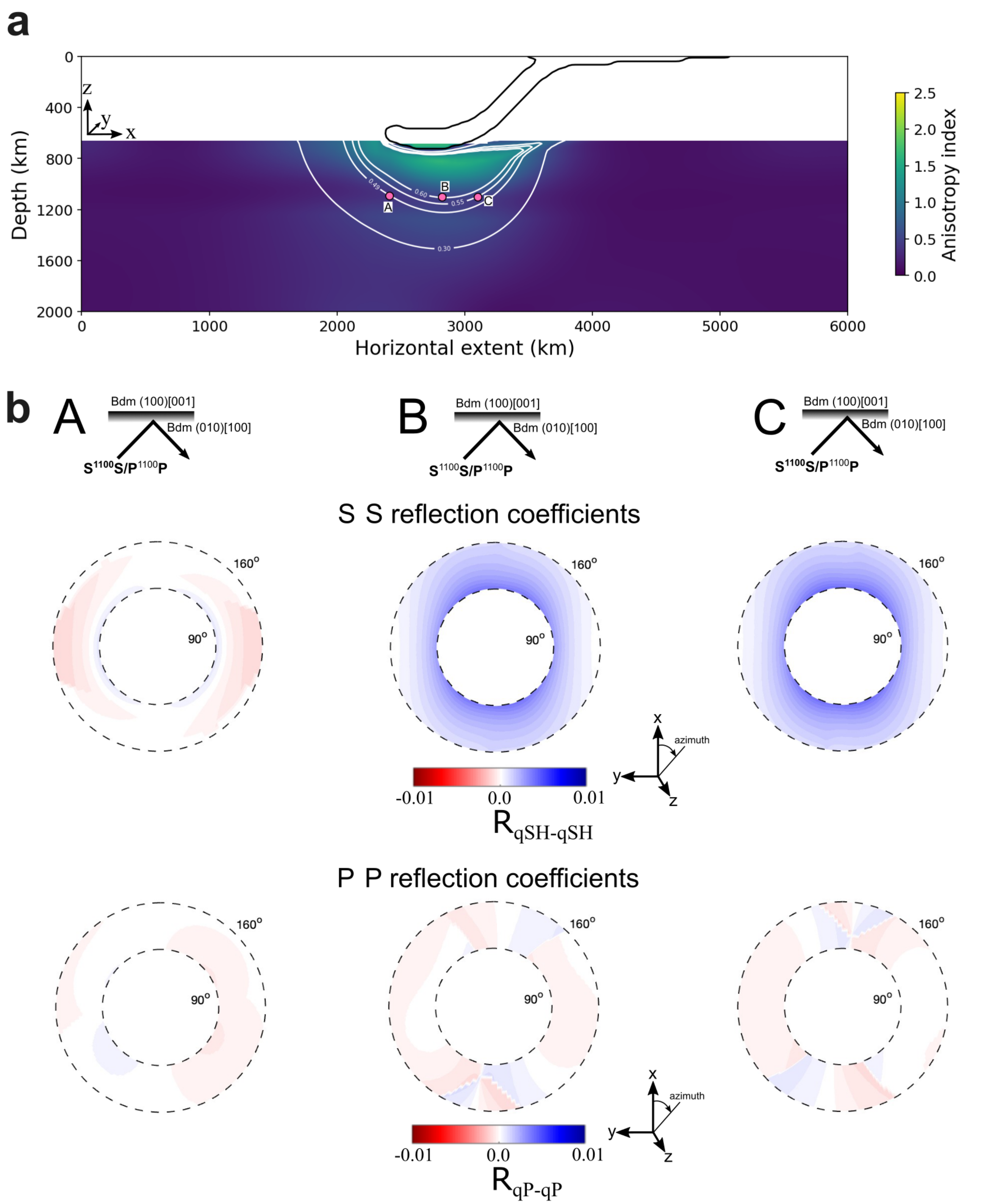

Supplementary Figure 2: **Anisotropic discontinuity for the shallowly stagnant slab model. a,** Anisotropy index for the shallowly stagnant slab model. Solid purple circles are the selected interfaces: Location A, Location B, and Location C along the anisotropic discontinuity where reflection coefficients are to be computed. **b,** Anisotropic reflection coefficients for qSH-qSH and qP-qP based on S^dS and P^dP phases, respectively. Despite persistence at 1000 km, the anisotropic discontinuity is very weak due to reduced strains.

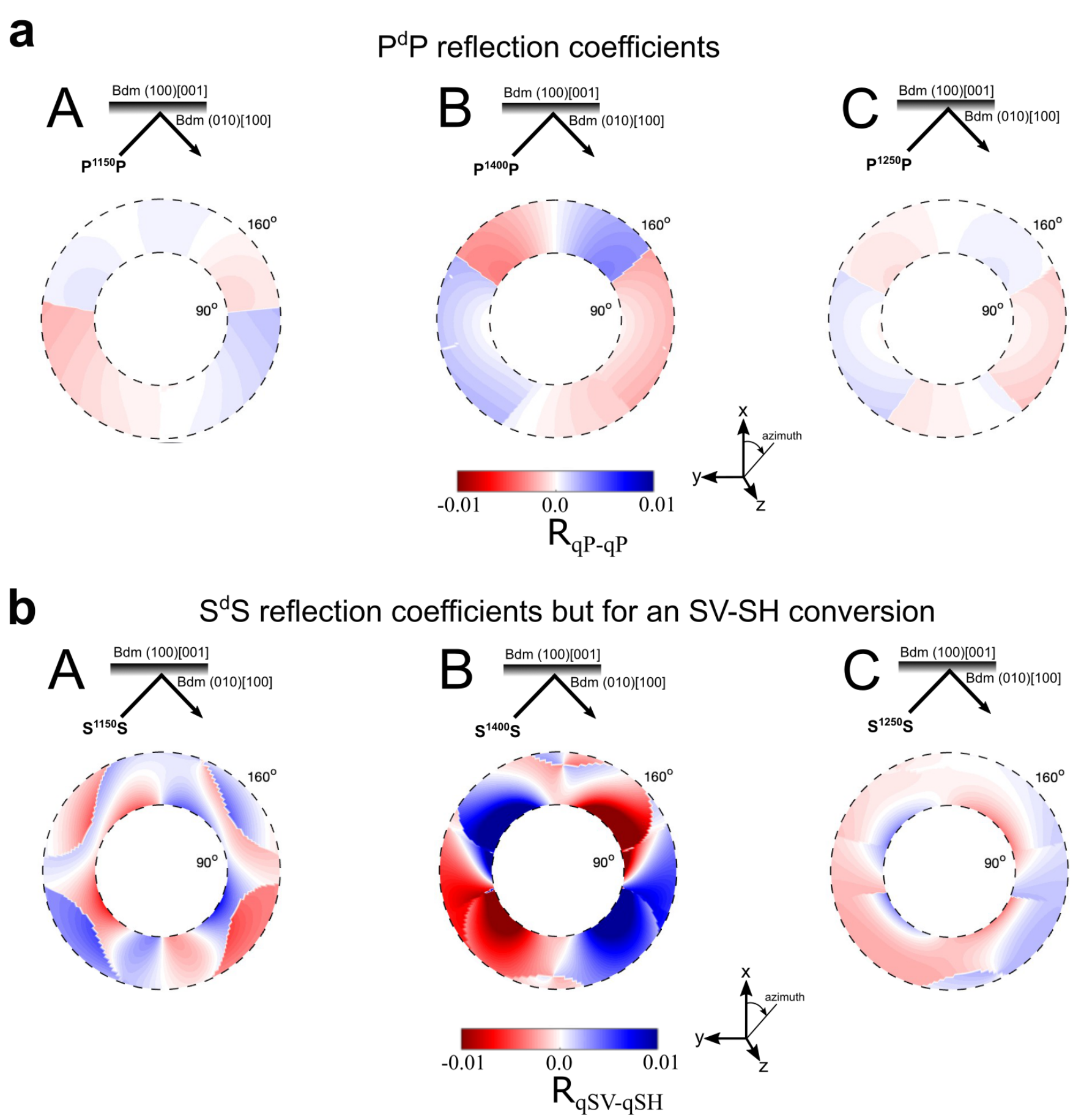

Supplementary Figure 3: **Supplementary reflection coefficients for the deeply flattened slab model. a,** Pole figure representation of qP-qP reflection coefficients with epicentral distance and azimuth at Locations A, B, and C in Fig. 2 of the Main Text suggestive of P^dP underside reflections off the anisotropic discontinuity. **b,** Same as (**a**) but for a qSV-qSH conversion. Such a conversion may arise due to the cross coupling of SH and SV motions for incident S-waves propagating in a generally anisotropic media[15] such as the deeply flattened slab model considered here.

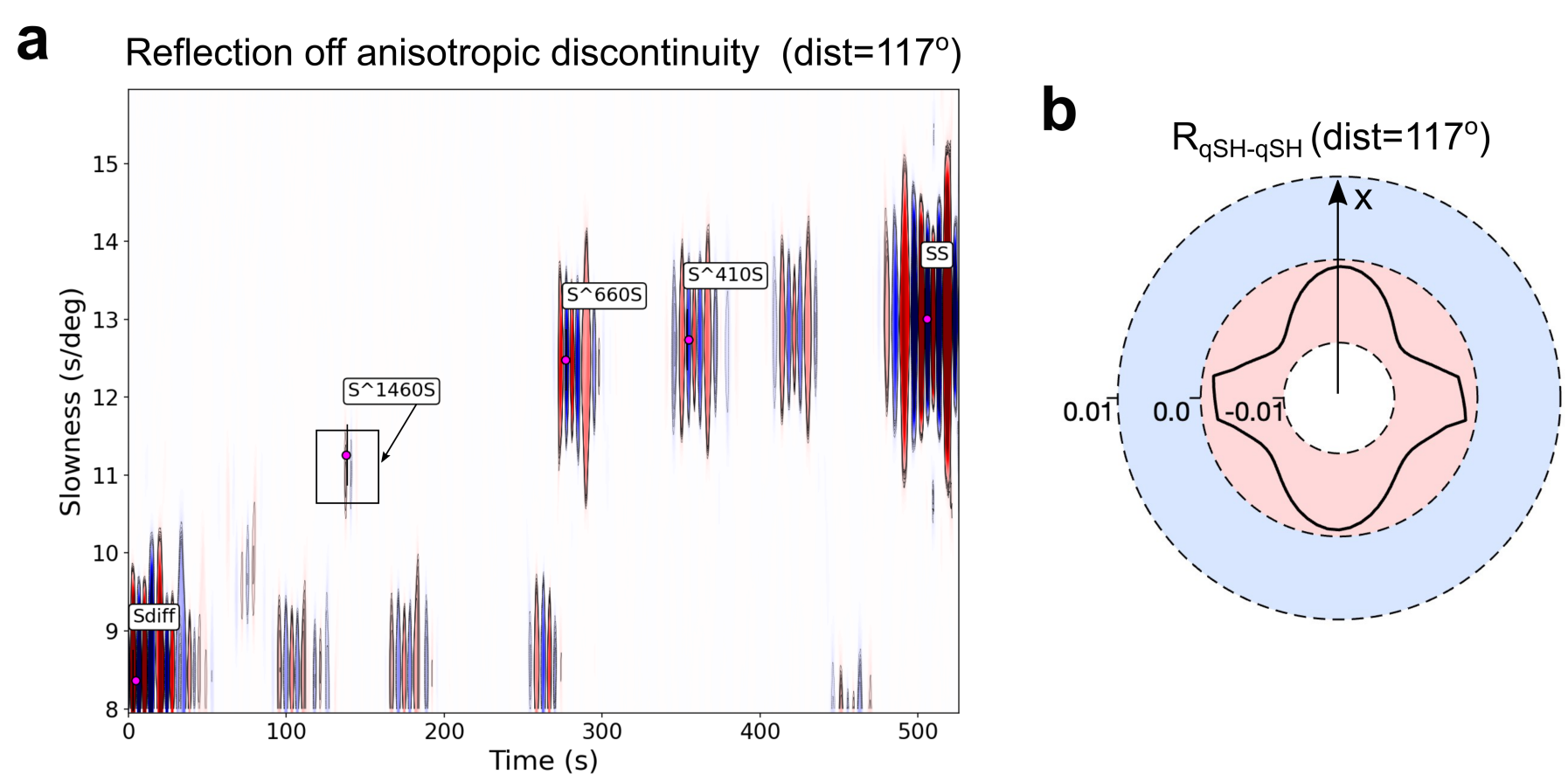

Supplementary Figure 4: **Detectability of the anisotropic discontinuity from a slightly larger distance. a,** Slowness vespagram for the fully-anisotropic mantle model. The measured distance from the source to the central receiver is 117°. **b,** Predicted reflection coefficients as a function of azimuth at the theoretical bounce point of (**a**). Instead of using a pole figure representation, we plot the reflection coefficients with azimuth only for the 117° distance for emphasis, similar to the representation of ref.[16]. Here, blue and red regions represent positive and negative values, respectively, for the reflection coefficients. Dashed concentric circles in increasing order delineate $R = -0.1, 0, 0.1$. Note the polarity reversal of $S^{1460}S$ relative to SS can be explained by its negative reflection coefficients at the interface.

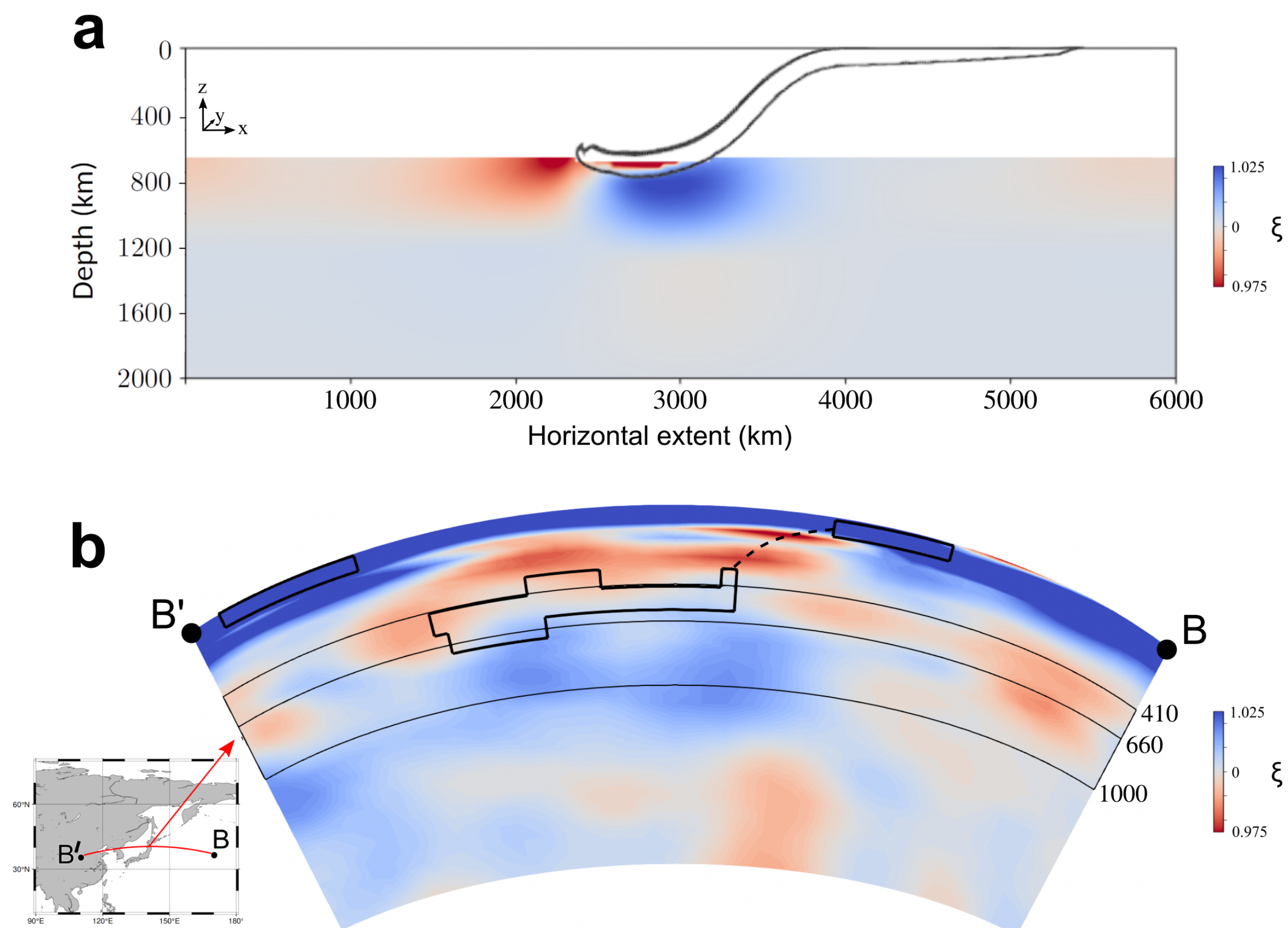

Supplementary Figure 5: **Radial anisotropy in the lower mantle for the shallowly stagnant slab model.** **a,** General patterns of large-scale radial anisotropy ξ in the lower mantle for a shallowly stagnating slab resulting from the texture evolution of pressure-dependent bridgmanite fabrics. **b,** Anisotropic tomography image of ξ in the Honshu subduction zone subduction zone retrieved from SGLOBE-Rani[17].

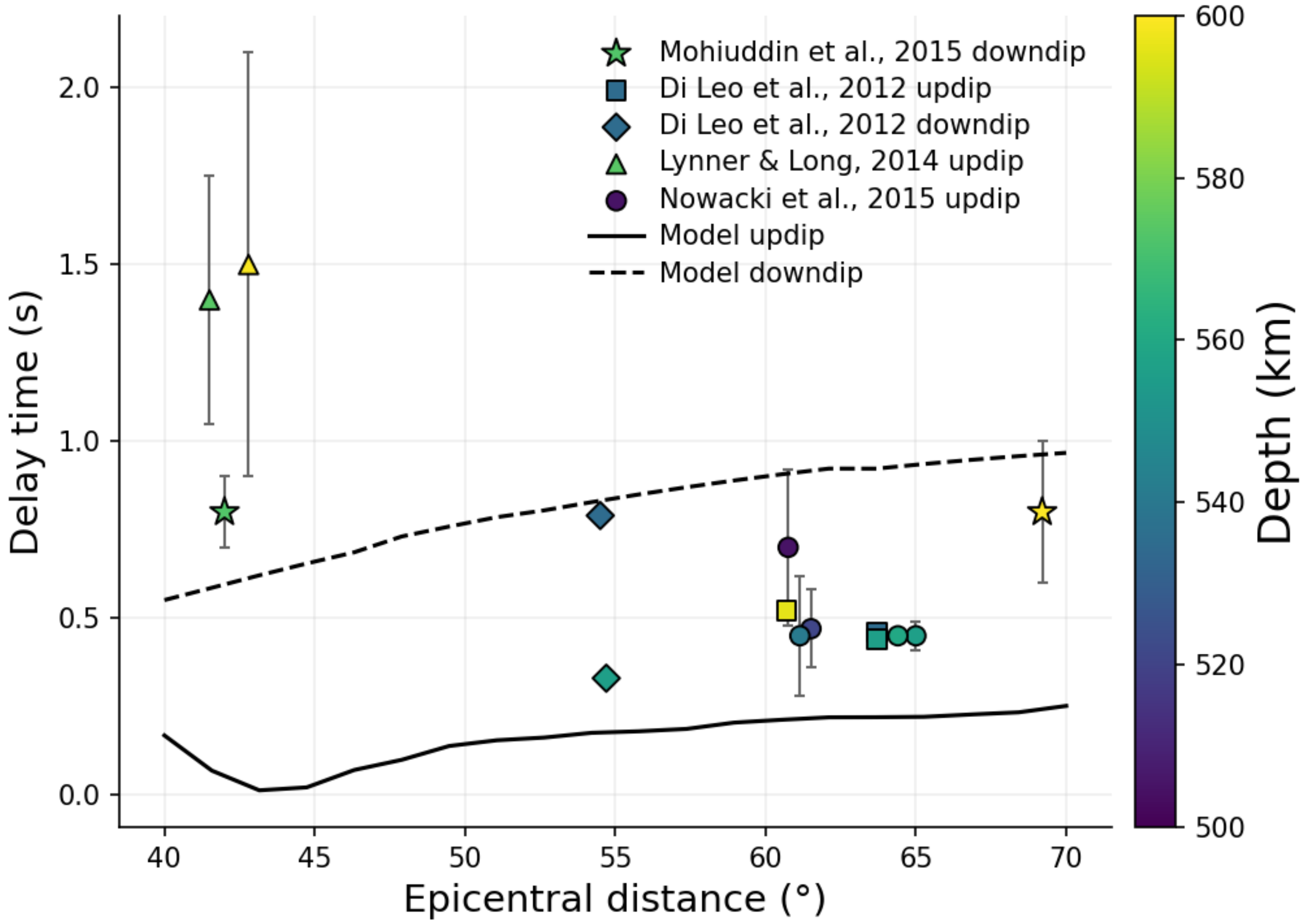

Supplementary Figure 6: **Observed delay times for subslab anisotropy beneath Western Java compiled from different studies.** Comparison of delay times predicted for our deeply flattened slab model (line plots) with observed delay times in the subslab mantle underneath Western Java (scatter plots) as a function of epicentral distance. Observed delay times and their corresponding error bars − representing the 95% confidence limits corresponding to each measurement − in both updip (i.e., ray path opposite to slab dip) and downdip (i.e., ray path in the same direction as slab dip) directions for events deeper than 500 km compiled from different studies are classified into several shapes: ref.[18] (square), ref.[18] (diamond), ref.[19] updip (triangle), ref.[20] downdip (star), ref.[21] updip (circle). Color gradient in each shape describes the event depth. Solid black line refers to our predicted delay times in the updip direction, whereas the dashed black line refers to that for the downdip direction.

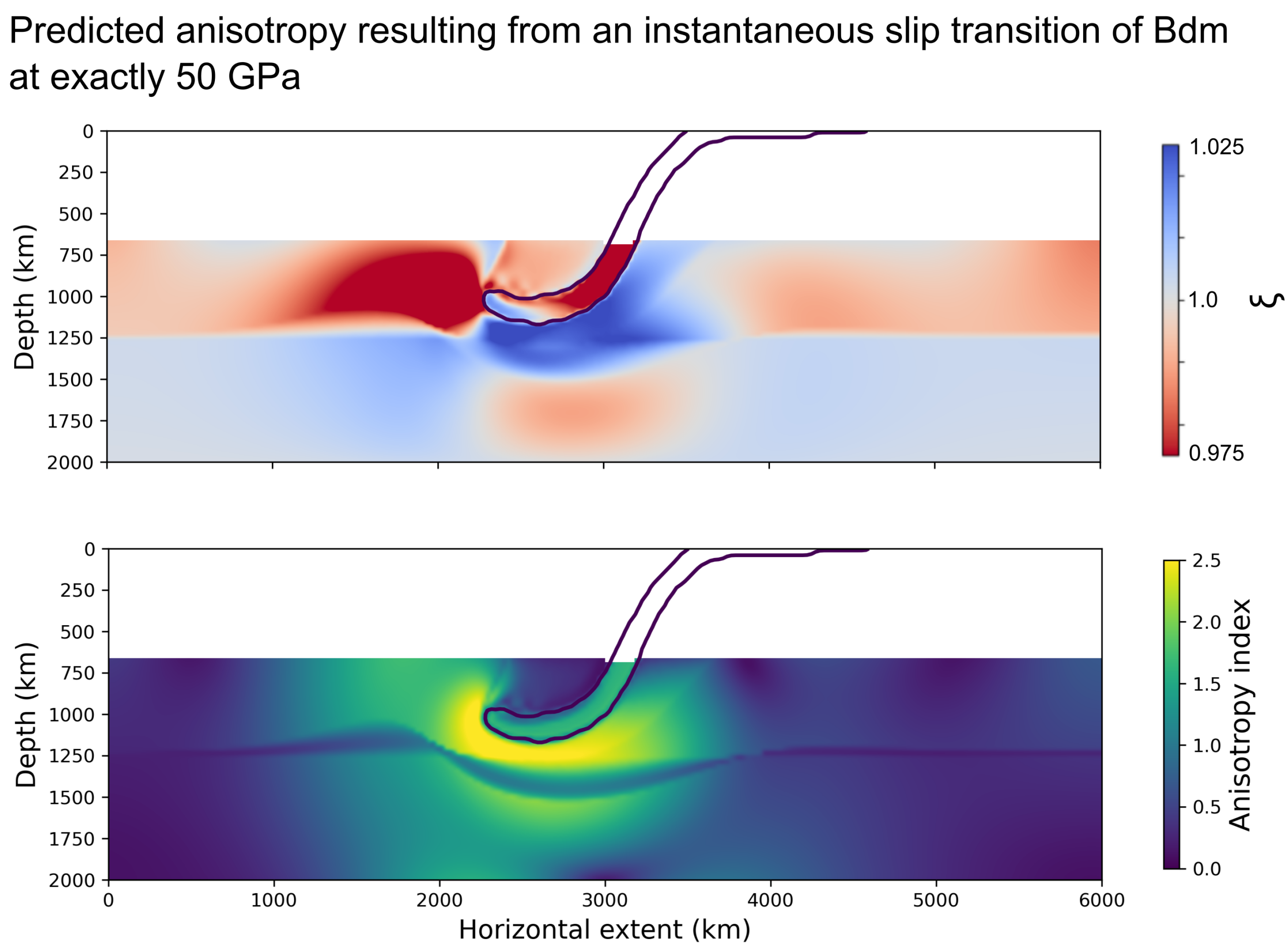

Supplementary Figure 7: **Fast fabric transition model.** Predicted large-scale radial anisotropy ξ (top panel) and anisotropy index (bottom panel) using the set of bridgmanite slip systems whose CRSS abruptly change at 50 GPa. Here, large-scale anisotropy patterns < 50 GPa are generated based on the CPO of a (100)[001] bdm fabric. Once the 50-GPa boundary is crossed, previous textures are immediately overwritten by an (010)[100] bdm fabric leading to a sharper anisotropic discontinuity.

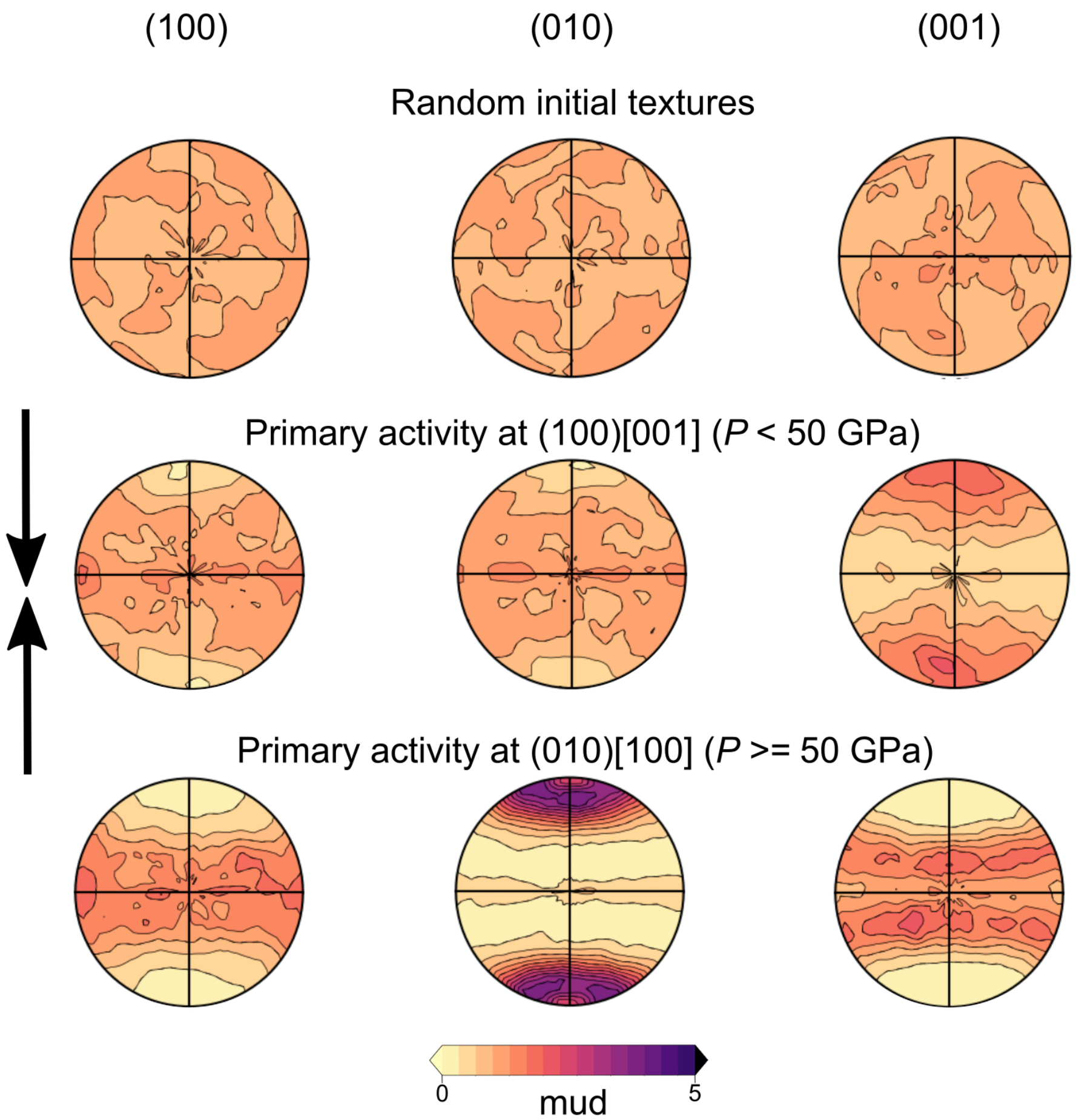

Supplementary Figure 8: **VPSC modeling of uni-axial compression.** VPSC simulation of uni-axial compression deformation on 3000 crystals making up the bridgmanite polycrystal aggregate. Starting with random initial textures (top panels), effective strains up to $\gamma_{\text{eff}} = 1$ produce preferential orientations of (001) axes with the compression direction (Dark arrows). Here, primary activity occurs at (100)[001] (middle panels). Past 50 GPa, primary activity transitions to (010)[100] leading to strong preferential orientations of the (010) axes with the compression direction (bottom panels)

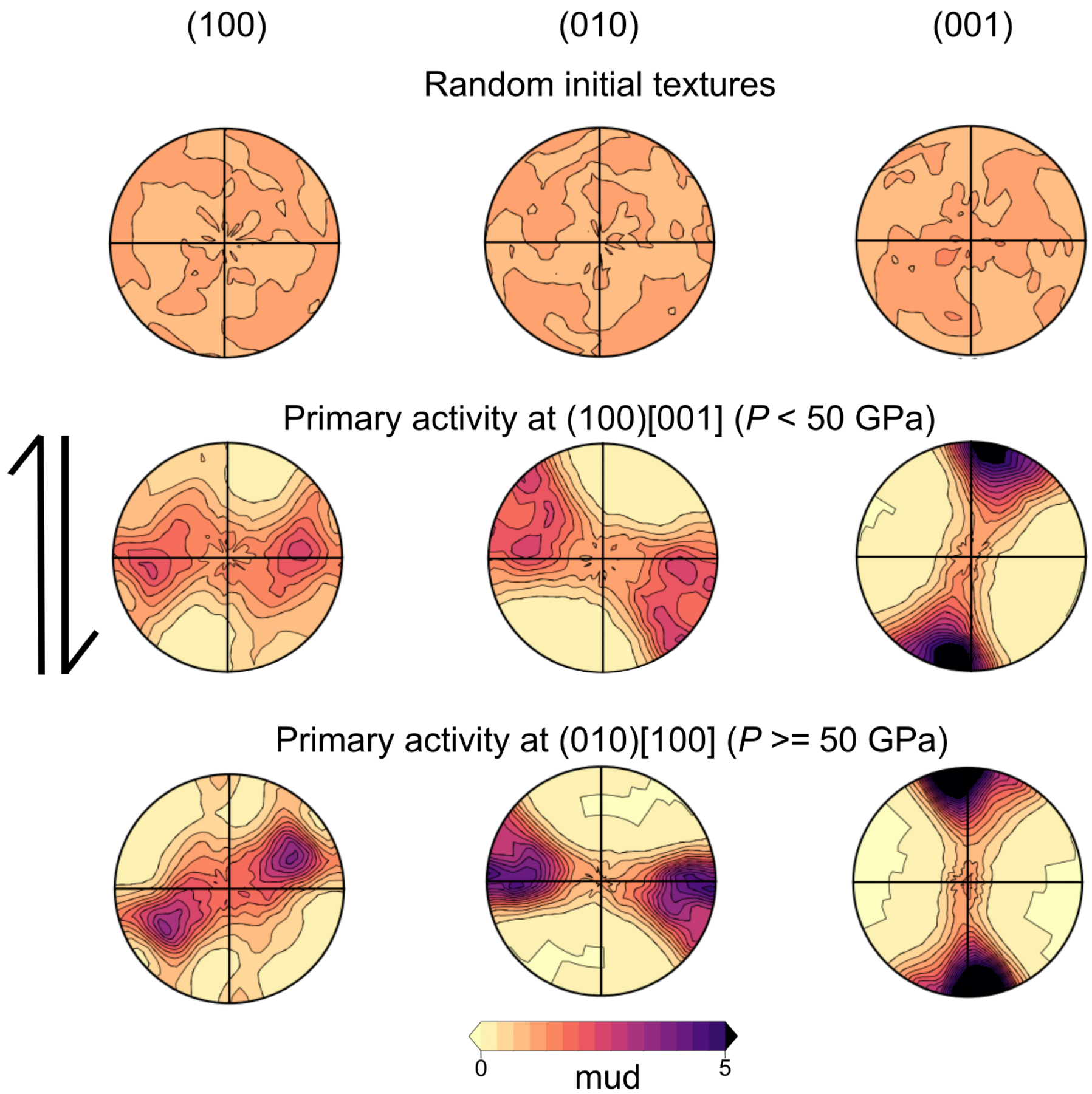

Supplementary Figure 9: **VPSC modeling of simple shear.** VPSC simulation of simple shear deformation on 3000 crystals making up the bridgmanite polycrystal aggregate. Starting with random initial textures (top panels), effective strains up to $\gamma_{\text{eff}} = 1$ produce near vertical (001) and oblique (010) textures. Here, primary activity also occurs at (100)[001] (middle panels). Past 50 GPa, primary activity transitions to (010)[100] leading to sub-horizontal (010) and vertical (001) textures (bottom panels). Dark arrows indicate shear direction.

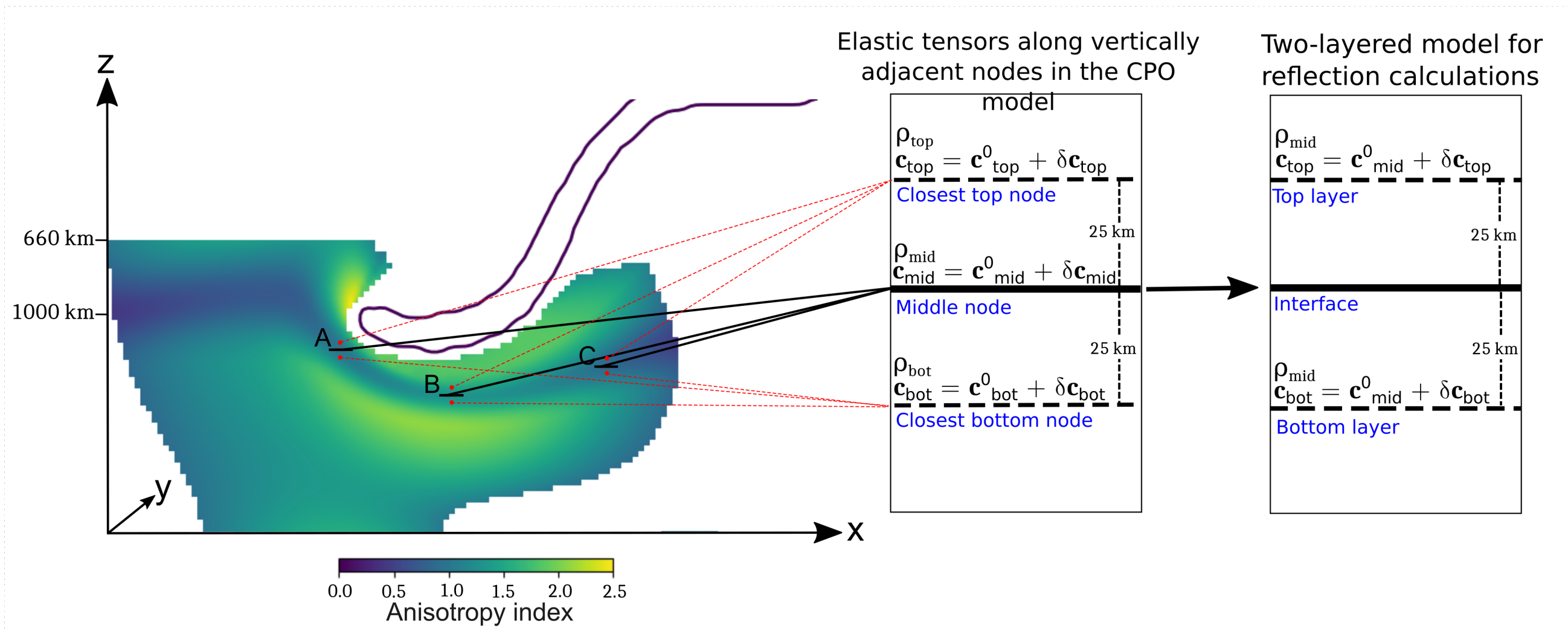

Supplementary Figure 10: **Modeling strategy to compute anisotropic reflection coefficients across the anisotropic discontinuity at Locations A, B, and C.** Here, $\mathbf{c}$ pertains to the full elastic tensor, $\mathbf{c}^0_{\text{top}}$ for its isotropic component, $\delta\mathbf{c}$ for its anisotropic component, and ρ for density at the interface labeled "mid", and top and bottom layers labeled "top" and "bot", respectively.

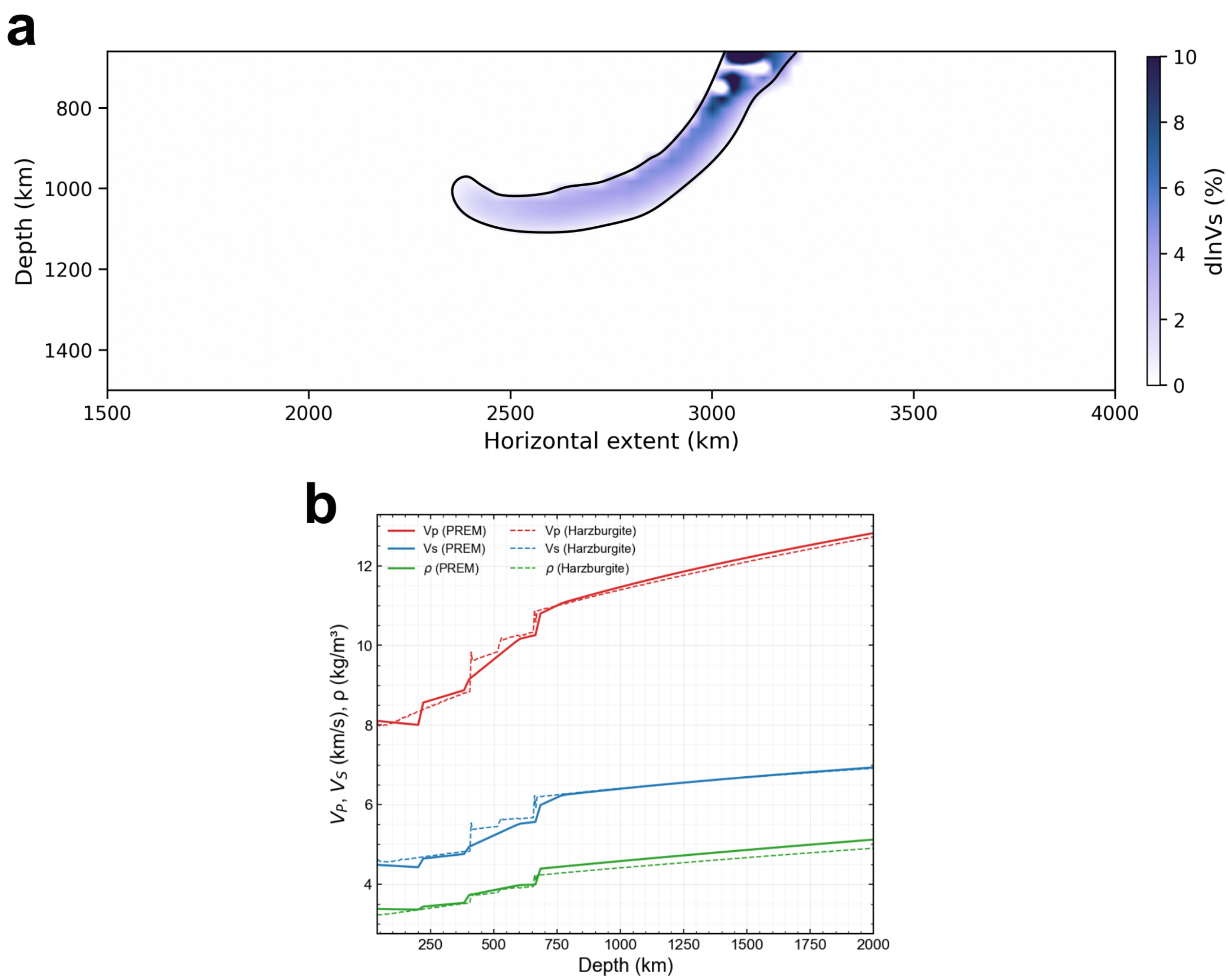

Supplementary Figure 11: **Modeling a harzburgitic slab. a,** V_S perturbation of the subducting slab composed of a harzburgite material relative to PREM. It is computed as follows: $dlnV_S = 2(V_S^{\mathrm{harz}} - V_S^{\mathrm{PREM}})/(V_S^{\mathrm{harz}} + V_S^{\mathrm{PREM}})$. **b,** Seismic velocities (V_P, V_S) and density (ρ) of harzburgite (dashed lines) and PREM (solid lines) as a function of depth. The seismic properties of harzburgite are computed using the revised 1D adiabatic temperature profile of ref.[22].

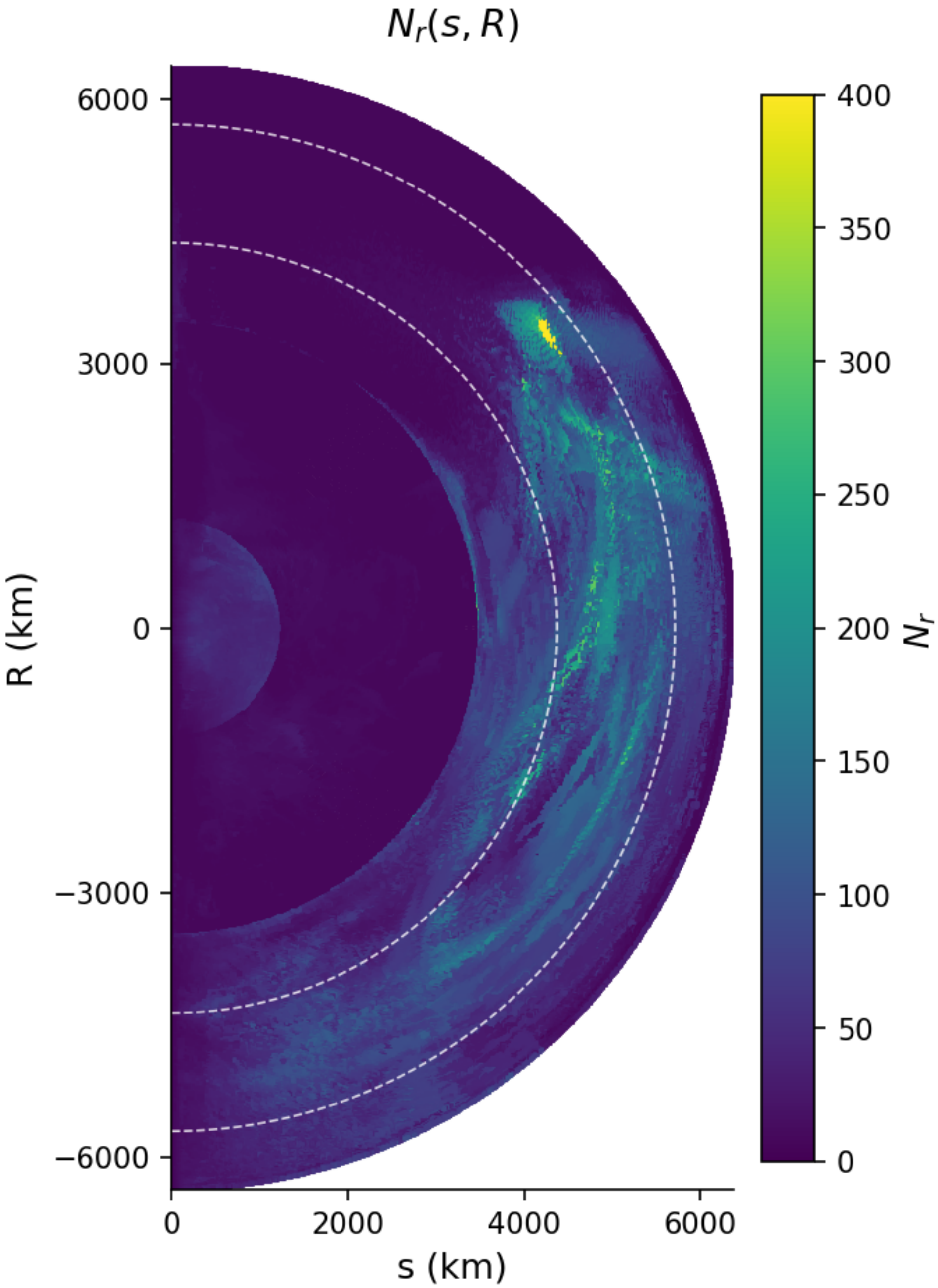

Supplementary Figure 12: **AxiSEM3D wavefield scanning.** AxiSEM3D wavefield scanning result from the test simulation with $Nr = 1000$ for the fully-anisotropic mantle model. Here, N_r is the Fourier coefficient order, $andR$ and s are in-plane coordinates. Color map refers to the minimum Fourier coefficient order required to accurately reconstruct azimuthal terms in both the wavefield and 3D structure. In our wavefield simulations, we thus set a constant N_r value of 400.

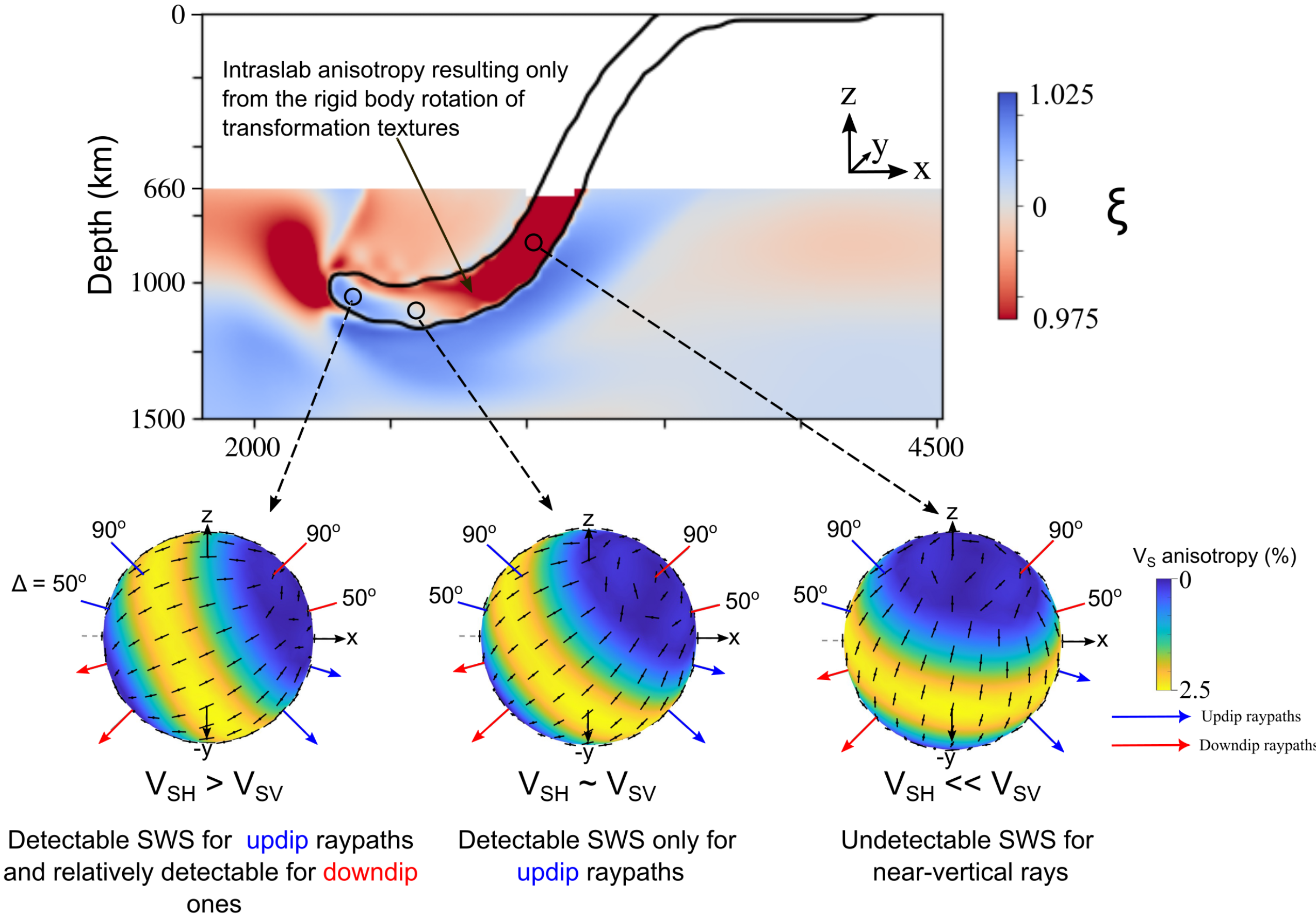

Supplementary Figure 13: **Intraslab anisotropy for the deeply flattened slab model.** S-wave anisotropy pole figures at selected zones inside the slab. Anisotropy amplitudes are expressed in percent; whereas fast directions are represented by the black arrow-less vectors. Arrows piercing through the sphere correspond to updip rays (in Blue) and downdip rays (in Red) incident to the anisotropic region (dark circles). Angles $50° - 90°$ represent the epicentral distance corresponding to direct S phases used in source-side shear wave splitting studies. Intraslab anisotropy is modeled after the ringwoodite→bridgmanite transformation textures constrained from experiments[6].

References

[1] Kronbichler, M., Heister, T. & Bangerth, W. High accuracy mantle convection simulation through modern numerical methods. *Geophysical Journal International* **191**, 12–29 (2012).

[2] Mitrovica, J. X. & Forte, A. M. Radial profile of mantle viscosity: Results from the joint inversion of convection and postglacial rebound observables. *Journal of Geophysical Research: Solid Earth* **102**, 2751–2769 (1997).

[3] Karato, S.-i. & Wu, P. Rheology of the upper mantle: A synthesis. *Science* **260**, 771–778 (1993).

[4] Davis, R. O. & Selvadurai, A. P. *Plasticity and geomechanics* (Cambridge university press, 2005).

[5] Magali, J. K., Thomas, C., Ledoux, E. E., Capdeville, Y. & Merkel, S. On the influence of pressure, phase transitions, and water on large-scale seismic anisotropy underneath a subduction zone. *Geochemistry, Geophysics, Geosystems* **26**, e2024GC011827 (2025).

[6] Gay, J. P. *et al.* Transformation microstructures in pyrolite under stress: Implications for anisotropy in subducting slabs below the 660 km discontinuity. *Earth and Planetary Science Letters* **604**, 118015 (2023).

[7] Eakin, C. M. *et al.* Internal deformation of the subducted nazca slab inferred from seismic anisotropy. *Nature Geoscience* **9**, 56–59 (2016).

[8] Agrawal, S., Eakin, C. M., Portner, D. E., Rodriguez, E. E. & Beck, S. L. The deformational journey of the nazca slab from seismic anisotropy. *Geophysical Research Letters* **47**, e2020GL087398 (2020).

[9] Goes, S., Agrusta, R., van Hunen, J. & Garel, F. Subduction-transition zone interaction: A review. *Geosphere* **13**, 644–664 (2017).

[10] Faccenda, M., Burlini, L., Gerya, T. V. & Mainprice, D. Fault-induced seismic anisotropy by hydration in subducting oceanic plates. *Nature* **455**, 1097–1100 (2008).

[11] Hao, M. *et al.* The seismically fastest chemical heterogeneity in the earth's deep upper mantle—implications from the single-crystal thermoelastic properties of jadeite. *Earth and Planetary Science Letters* **543**, 116345 (2020).

[12] Ferreira, A. M., Faccenda, M., Sturgeon, W., Chang, S.-J. & Schardong, L. Ubiquitous lower-mantle anisotropy beneath subduction zones. *Nature Geoscience* **12**, 301–306 (2019).

[13] Hirth, G. & Kohlstedf, D. Rheology of the upper mantle and the mantle wedge: A view from the experimentalists. In Eiler, J. (ed.) *Inside the Subduction Factory cover imageInside the Subduction Factory*, vol. 138 of *Geophysical Monograph Series*, 83–105 (American Geophysical Union, 2003).

[14] Gay, J. P. *et al.* Depth dependent deformation and anisotropy of pyrolite in the earth's lower mantle. *Geophysical Research Letters* **51**, e2024GL109433 (2024).

[15] Keith, C. M. & Crampin, S. Seismic body waves in anisotropic media: synthetic seismograms. *Geophysical Journal International* **49**, 225–243 (1977).

[16] Thomas, C., Wookey, J., Brodholt, J. & Fieseler, T. Anisotropy as cause for polarity reversals of d" reflections. *Earth and Planetary Science Letters* **307**, 369–376 (2011).

[17] Chang, S.-J., Ferreira, A. M., Ritsema, J., van Heijst, H. J. & Woodhouse, J. H. Joint inversion for global isotropic and radially anisotropic mantle structure including crustal thickness perturbations. *Journal of Geophysical Research: Solid Earth* **120**, 4278–4300 (2015).

[18] Di Leo, J. *et al.* Mantle flow in regions of complex tectonics: Insights from indonesia. *Geochemistry, Geophysics, Geosystems* **13** (2012).

[19] Lynner, C. & Long, M. D. Sub-slab anisotropy beneath the sumatra and circum-pacific subduction zones from source-side shear wave splitting observations. *Geochemistry, Geophysics, Geosystems* **15**, 2262–2281 (2014).

[20] Mohiuddin, A., Long, M. D. & Lynner, C. Mid-mantle seismic anisotropy beneath southwestern pacific subduction systems and implications for mid-mantle deformation. *Physics of the Earth and Planetary Interiors* **245**, 1–14 (2015).

[21] Nowacki, A., Kendall, J.-M., Wookey, J. & Pemberton, A. Mid-mantle anisotropy in subduction zones and deep water transport. *Geochemistry, Geophysics, Geosystems* **16**, 764–784 (2015).

[22] Katsura, T. A revised adiabatic temperature profile for the mantle. *Journal of Geophysical Research: Solid Earth* **127**, e2021JB023562 (2022).
