# Supplementary material for: Mid-mantle discontinuities beneath subduction zones linked to depth-dependent deformation of bridgmanite
Source: Nat Commun. 2026 Oct 5;17:10494. doi: 10.1038/s41467-026-78212-6 (PMC13639073; doi:10.1038/s41467-026-78212-6)
Supplement: Supplementary file 2 [file 41467_2026_78212_MOESM2_ESM.pdf]

nature portfolio

Peer Review File

Mid-mantle discontinuities beneath subduction zones linked to depth-dependent deformation of bridgmanite

Corresponding Author: Dr John Keith Magali

This file contains all reviewer reports in order by version, followed by all author rebuttals in order by version.

Attachments originally included by the reviewers as part of their assessment can be found at the end of this file.

Version 0:

Reviewer comments:

Reviewer #1

(Remarks to the Author)

Reviewer #3

(Remarks to the Author)
Review of "Mid-mantle discontinuities beneath subduction zones linked to depth-dependent deformation of bridgmanite"
Daniel A Frost
This paper introduces a new mechanism of generating seismic anisotropy in the uppermost lower mantle ~1000 km depth by development of texture in bridgmanite. This textural development is facilitated by increased pressure below the slab triggering a change in dominant slip system. The authors suggest that this anisotropy could cause a seismic velocity contrast at ~1000 km depth, which could generate SdS precursors which have been modelling to occur at these depths in subduction zones and in other areas of the mantle.

This paper suggests an interesting new mechanism that is motivated by mineral physics measurements of the CRSS in bridgmanite and brings together mineral physics and geodynamics to interpret seismic observations. This submission of the paper improves over the previous version by more clearly stating the limits of this phenomenon: that it could only explain mid-mantle anomalies around subduction zones, does not explain PdP reflectors, and is not the only possible cause of mid-mantle reflectors. I appreciate the addition of the synthetic seismograms and the vespagrams to confirm the theoretical existence of these phases. My main remaining question is about whether the amplitude of the synthetic SdS wave matches that from data. Also, the sentence structure used can be confusing but could be improved by breaking up the long sentences. I have highlighted some examples in this review.

General comments
1. How strong are the SdS waves generated by the anisotropy contrast? Studies such as Waszek et al., (2016) stack multiple traces and find SdS precursors of amplitudes around half that of transition zone phases S440S and S660S.

2. I think that the authors need to explain their definition of slab stagnation. For instance, in Figure 6, I view both of these scenarios as stagnation. These scenarios differing only in the depth of stagnation, either above 660 km or above 1000 km. This relates to my comment below about line 168.

3. Is anisotropy of olivine above 660 km depth neglected throughout these calculations? How would shallow mantle anisotropy affect the path-integrated shear wave splitting?

Line-by-line comments
Line 41: The following sentence implies that SS reflectors only occur in subduction zones, which is not true. I suggest rewriting this to remove that implication. "In subduction settings, reflectors and scatterers within this depth range are well-documented using SS precursors (i.e., S-wave underside reflections off depth d)4–7"

Lines 75-75: These feel out of place since anisotropy resulting from Bridgmanite was introduced in the prior paragraph.

Line 120: Suggest changing “with distance” to “with distance away from the slab”

Line 168: The authors imply that no stagnating slab could not generate anisotropy by this mechanism. I don’t feel that this is sufficiently tested to draw this conclusion. The authors test a slab that stagnates at 660 km depth, therefore has only just passed into the anisotropy-forming region. However, some slabs, such as Tonga and South America (Fukao and Obayashi, 2013) are observed to stagnate at 1000 km, similar to the depth in their main subduction model. I think instead stating that shallowly stagnating slabs could not generate this anisotropy would be a more reasonable claim.

Limes 174-176: Why are arrays necessary? Is this because the amplitudes of these phases are so low?

Line 232: What “delay times” are being referred to? Supplementary Figure 6 also does not describe what delay times are being used.

Lines 247-250: I can’t fully understand the following sentence “Finally, sourceside SWS assumes similar receiver-side ray paths for S and SK(K)S phases. This assumption may break down at shorter epicentral distances wherein the estimates become potentially vulnerable to contamination from receiver-side anisotropy.”.

1- How would shorter epicentral distances cause more contamination by receiver-side anisotropy than source-side anisotropy? If greater epicentral distance data are more accurate, could the data be filtered by epicentral distance to reduce bias?
2- It’s not clear if this sentence refers to modelling or observations. Is the reason for this sentence to imply that the observations are not accurate representations of the true anisotropy?

Line 282: The implication of the following sentence is unclear. “In the upper MTZ, the olivine–wadsleyite and wadsleyite–ringwoodite transitions act as textural “filters” where coherent textures serve as host sites in the growth of incoherent textures with faster nucleation rates78,79.” What are these nucleation rates faster than? Does it mean that a random texture can grow quickly and overgrow a pre-existing preferred orientation? If so, I would recommend stating that.

Lines 287-289: Repetition from earlier discussion of slab stagnation. This line also relates to my earlier comment about how slab stagnation at depths deeper than 660 km may still cause texturing.

Lines 479-483: Are these coordinates [lon, lat] pairs or [lat, lon] pairs?

Figures and Tables
Figure 3: Recommend highlighting the important phases in the vespagrams, maybe with a box.

Spelling and Grammar
Line 16: Add “The” before the word “bulk”.

Line 234: What is the “its” in the phrase “surrounding its estimates”?

Lines 238-239: The following sentence is very long and confusing. I suggest rewriting this. “Assuming the mantle velocity gradients permit spatially-coherent fast axis orientations from these minerals, their effects could interfere constructively among layers with different fabrics, leading to increased delay times.”

Lines 252-253: I suggest rewriting the following sentence from “Because the frequency band considered in our waveform modeling overlaps with the dominant
253 frequency of SdS, whether the discontinuity persists at higher frequencies is beyond this work.” To “We model frequencies matching the dominant observed frequency of SdS. Whether the discontinuity persists at higher frequencies is beyond this work.”

Lines 267-269: I suggest rewriting the following sentence from “This localizes larger strains beneath it which could help form stronger (100)[00] textures46, but this time, over nearly untextured bridgmanite across the viscosity jump.” To “This localizes larger strains beneath the slab which could help form stronger (100)[00] textures sharply contrasting with nearly untextured bridgmanite below the viscosity jump.”

Line 277: Change “accommodation respond” to “accommodation to respond”

Lines 292-293: The first clause of the following sentence is negated by the later detail “Barring full penetration into the lowermost mantle, subduction behavior is typically classified into three modes: (1) penetration, (2) stagnation near 660 km, and (3) trapping near 1000 km”. I suggest rewriting this as “Subduction behavior is typically classified into three modes: (1) penetration, (2) stagnation near 660 km, and (3) trapping near 1000 km.”

Line 356: Change “harmonic averaged” to “harmonically averaged”

Line 473: Change “wit” to “with”

Reviewer #4

(Remarks to the Author)
The authors provide extensive modeling for the potential generation of anisotropy at lower mantle depths beneath subducting slabs. Their work is useful, and could provide a fruitful avenue for understanding the dynamics of certain regions in the lower mantle. I was not an original reviewer, but I think the authors have done an adequate job of responding to reviewer comments and suggestions. I have only minor comments that I think could improve readability.

Version 1:

Reviewer comments:

Reviewer #1

(Remarks to the Author)

Reviewer #3

(Remarks to the Author)
Review of “Mid-mantle discontinuities beneath subduction zones linked to depth-dependent deformation of bridgmanite”
Daniel A Frost
This paper introduces a new mechanism of generating seismic anisotropy in the uppermost lower mantle ~1000 km depth by development of texture in bridgmanite. This textural development is facilitated by increased pressure below the slab triggering a change in dominant slip system. The authors suggest that this anisotropy could cause a seismic velocity contrast at ~1000 km depth, which could generate SdS precursors which have been modelling to occur at these depths in subduction zones and in other areas of the mantle.

The paper has been further improved by this second revision. I am mostly satisfied with the changes made. I have a few comments and recommended changes.

General comments
1. While the authors have changed their terminology somewhat to describe a “shallowly stagnating slab”, the main model they show in Figure 1 still looks like a stagnating slab, despite it being described as a “penetrating slab”. I feel that readers may be confused by this terminology.

2. The authors responded to my previous comment about olivine anisotropy being ignored here but did not change the text. I feel that this is an important point to make clear in the text, especially since the anisotropy of other minerals is introduced in line 248. Olivine, the main contributor to mantle anisotropy, is conspicuous by its absence. A sentence describing the authors’ reason for ignoring this feels important.

Line-by-line comments
Lines 189-191: I think that the following sentence could be revised to improve clarity, “For a purely anisotropic mantle model without any isotropic perturbations coming from the slab, the anisotropic discontinuity generates a clear SdS arrival at 112∘ (Fig. 3a)”. I suggest something like, “For a model with only anisotropic velocity contrasts from induced texture and no isotropic velocity perturbations resulting from the slab, the anisotropic discontinuity caused by the slab generates a clear SdS arrival at 112∘ (Fig. 3a)”.

Line 194: Suggest altering the following phrase, “but with a reversed SdS polarity relative to SS”.

Line 246: The authors have added a little more detail to describe the delay times, but I recommend describing what the delay times are of. I think it’s the anisotropic delay time between the SV and SH S-wave, but it is not clear.

Figures and Tables
Figure 2. Recommend adding “α” to figure caption and colour scale label in 2a.

Spelling and Grammar
Line 47: “The variability is unclear whether the signals reflect coherent discontinuities or instead originate”. The grammar here appears incorrect, but I don’t know how as I can’t determine what is intended.

Line 51: In “However, this interpretation” suggest changing “this interpretation” to “these interpretations”

Line 220: The following sentence appears incorrect, “Previous studies using (applying?) kinematic approaches to (model?) mantle fabric calculation explored various”.

Open Access This Peer Review File is licensed under a Creative Commons Attribution 4.0 International License, which permits use, sharing, adaptation, distribution and reproduction in any medium or format, as long as you give appropriate credit to the original author(s) and the source, provide a link to the Creative Commons license, and indicate if changes were made.
In cases where reviewers are anonymous, credit should be given to 'Anonymous Referee' and the source.
The images or other third party material in this Peer Review File are included in the article's Creative Commons license, unless indicated otherwise in a credit line to the material. If material is not included in the article's Creative Commons license and your intended use is not permitted by statutory regulation or exceeds the permitted use, you will need to obtain permission directly from the copyright holder.
To view a copy of this license, visit https://creativecommons.org/licenses/by/4.0/

We thank you and the three reviewers for the thorough feedback of our manuscript during this second round of review at *Nature Communications*. We now have carefully addressed the comments and suggestions, and have revised the manuscript accordingly to improve its clarity and readability. This document provides our point-by-point response to the reviewers and indicates which parts of the manuscript have been revised. Here, the reviewers' comments are shown in black, our responses in blue, and some excerpts from the revised manuscript in red. We hope that the revised manuscript is now ready for further consideration at *Nature Communications*.

REVIEWER COMMENTS

Reviewer #1 (Remarks to the Author):

Comments on "Mid-mantle discontinuities beneath subduction zones linked to depth-dependent deformation of bridgemanite" by Magali et al.

This paper reports on the combination modelling of important processes associated with the deformation of the Earth' lower mantle near subduction zones. The main idea proposed by the authors, discontinuous change in anisotropic structure as the cause of seismic anomalies in the mid-lower mantle, is interesting. I think this paper deserves publication in this journal. In general, the manuscript is adequately structured and the descriptions in the main text seem succinct, maybe too succinct forced by the tight length limit of the article of the journal. Here I raise several issues to be clarified further before accepting this paper. Most of my comments seem to be related to the meaning of "sharpness" of the discontinuity structure, which is not necessarily clearly presented throughout the manuscript.
We thank you for your thorough review. We have revised the manuscript to define 'sharpness' more explicitly in the context of our anisotropic discontinuity mechanism. Here, 'sharpness' refers to the vertical width over which the anisotropy index decreases across the modeled transition. We now believe that this removes the ambiguity in its definition, and would help improve the readability of the revised manuscript.

1. Line 124-127 "In the mantle wedge and sub slab region, ... Coincidently, this is also ... "
I was a bit puzzled by reading these sentences. Is the agreement between the discontinuity and the depth where the CRSS values of the (100)[001] and (010)[100] slip systems switch really a coincidence? They are expected to coincide with each other in the framework of the authors' model, aren't they?
Thank you for pointing this out. We agree that the emergence of the anisotropic discontinuity and the intersection of the CRSS values for (100)[001] and (010)[100] slip systems (Fig. 1c) are expected to be linked within the framework of our model. Our intention was to emphasize that the CRSS crossover provides the mineral-physics basis for the onset of the modeled anisotropic discontinuity near ~1000 km depth. We now removed the word 'coincidentally' and revised the text to make this causality explicit:
"In the mantle wedge and subslab regions, the anisotropic discontinuity starts to emerge near ~1000 km. This corresponds to the depth where the critically-resolved shear stress (CRSS) values of the (100)[001] and (010)[100] slip systems intersect (dashed red line at 40 GPa in Fig. 1c), marking the onset of a transition in the dominant slip system that accommodates strain."

Also does the first sentence refer to Figure 2a? If so, the argument of the sentence is not necessarily validated by the figure, I think.

Indeed, the first sentence refers to Figure 2a (which is now cited at the end of the sentence for clarity). We agree that Figure 2a alone mainly shows the anisotropic discontinuity, whereas the depth at which it emerges is physically explained by the depth-dependent CRSS shown in Fig. 1c. Our revision of Lines 124-127 include citations both to Figures 1c and 2a for clarity.

2. Line 129-133 “Its sharpness is well correlated with increasing deformation in the surrounding mantle despite the smooth transition in bridgmanite slip systems with pressure”

The meaning of this sentence is not clear. It is perhaps because the term “sharpness” is not very well defined.

We understand your concern in this matter. We have revised the manuscript to define sharpness across the anisotropic discontinuity as the vertical half-width of the local reduction in anisotropy index \alpha. Since the anisotropic discontinuity appears as a local minimum in \alpha rather than as a step-like or a monotonic gradient, we measure this half-width from the local minimum to the depth where \alpha recovers to a value corresponding to a fixed anisotropy index contrast. Under this definition, a sharper discontinuity corresponds to a narrower zone over which \alpha is reduced.

Under *Methods* we have now included:

“We quantify the sharpness of the anisotropic discontinuity using a fixed threshold, defined as the vertical half-width over which α recovers from its local minimum, $\alpha_{\min}$ to a value corresponding to a 20$\%$ anisotropy index contrast: $2(\alpha-\alpha_{\min})/(\alpha+\alpha_{\min})$.”

Applying this definition to the selected locations gives half-widths of ~54 km, ~40 km, and ~80 km for Locations A, B, and C, respectively, making Location B the sharpest interface among the three locations. In the main text, we have only mentioned the half-width comparisons for brevity and conciseness (please see our response to Point 6).

3. Line 120-123 “... where anisotropy index is drastically reduced.”

This comment is related to the previous one. Figure 2a gives me an impression that reduction of the anisotropy index occurs very gradually. Please discuss the sharpness of the anisotropy discontinuity more quantitatively, if possible.

We thank you for this comment. We have rephrased the sentence to describe the modeled structure more precisely:

“Mapping the anisotropy index α (i.e., a scalar value that quantifies the percentage of total anisotropy in the elastic tensor; see Methods) reveals this feature as zones in the surrounding mid-mantle (>1000 km) where α exhibits a spatially persistent local minimum (Fig. 2a).”

We do not include the quantitative discussion in this sentence because it serves as the initial visual description of the anisotropic discontinuity in Figure 2a. Instead, the quantitative definition and comparison of sharpness in terms of the half-widths are introduced in the following paragraph, where the selected Locations A, B, and C along the discontinuity are discussed.

4. Line 143-144 “We base our computations on SdS, ..., enables sensitivity to even subtle discontinuities, ...”

The meaning of this sentence is not clear, either. Do the authors claim that, although the discontinuity structure of their dynamic model is subtle and smooth, the waveform analyses method they use enables detecting the structure?

Thank you for pointing this out. Our intention was not to claim at this point that our modeled discontinuity is directly detectable by waveform analysis. Rather, we meant that S^dS phases are suitable for investigating this class of structures since they can be sensitive not only to sharp discontinuities but also to gradients in seismic velocities owing to their broad period nature (e.g., Deuss and Woodhouse, 2004; Deuss, 2009). In addition, a previous work has shown that S^dS phases can be sensitive to anisotropic discontinuities, even without an imposed isotropic velocity or density jump (e.g., Rychert et al., 2014). We have therefore revised this sentence to state why S^dS are appropriate for the anisotropic discontinuity predicted by the depth-dependent bridgmanite fabric:
“We base our computations on S^{d}S phases, whose occurrence across multiple period bands\citep{waszek2018global,frazer2023high,yuan2025mantle} enables sensitivity to a broad range of seismic structures, including weak or gradational seismic discontinuities\citep{deuss2004nature,deuss2009global} and anisotropic discontinuities even without an imposed isotropic velocity or density jump\citep{rychert2014synthetic}.”

5. Line 439- Section “Anisotropic reflection calculations “
By reading the Method section of the manuscript I had an impression that in their model each reflector is regarded as a first order discontinuity not as a gradual transition in the anisotropy index. But the descriptions are not very clear-cut about this point, so please clarify this point. Please also clarify whether the dip of the reflector is considered or modelled as a horizontal interface.
We thank you for this comment. We indeed treated the anisotropic interfaces as a first-order discontinuity. Furthermore, we also assume a horizontal interface and did not include the reflector dip in the reflection coefficient calculations. In relation to Reviewer 4’s suggestion, we have merged *Anisotropic reflection calculations* and Supplementary Text 3, where we have now also clearly stated these assumptions:
“The elastic tensor corresponding to this interface is called $\mathbf{c}_\text{mid}$, whereby it is treated as a local first-order discontinuity between the top and bottom layers.”
And:
“Assuming a horizontal interface, we treat the problem as S^{d}S reflections in the underside of the anisotropic discontinuity where d pertains to the depth at which reflection occurs.”
We note this approximation is useful for evaluating the local first-order seismic response at the anisotropic discontinuity. In related work, the same reflection coefficient modeling for the anisotropic discontinuity may explain the deeper (~1000 km) mid-mantle reflectors beneath subduction zones (Yuan, Magali et al., 2026; https://doi.org/10.1016/j.epsl.2026.120074).

6. Line 131 “Locations A and B exhibit a sharper contrast...”
I was a bit confused by the sentence, because it refers to the absolute values of anisotropy indexα, not its contrast. Please clarify this point.
We thank you for this comment. Relative sharpness comparisons at the selected locations A, B, and C are now quantified in terms of the half-widths defined earlier:
“For instance, Locations A and B (purple circles in Fig. 2a) exhibit relatively sharper anisotropic discontinuities (half-widths of $\sim$40 km and $\sim$54 km, respectively) owing to larger natural strains at the interface ($E_0>2$) than that of Location C (half-width of $\sim$80 km), consistent with its lower strain ($E_0 \sim 1.4$).”

7. Line 413-418 “... the predicted anisotropy produced by an instantaneous change...”
The meaning of these sentences was not clear. Do they mean the model predict only a gradual change in the anisotropy structure? What does the term “transition” mean? Referring here to Supplementary Fig.9 is not kind to potential readers, I think. The description including

“...unrealistic anisotropy patterns ...” is not very understandable, either. Somewhat a short and clear-cut explanation is desirable in the main text.

We thank you for pointing this out. We have now made clear in the main text that imposing gradual slip transitions in bridgmanite with depth produces anisotropic discontinuities characterized by gradational changes in seismic anisotropy over a depth interval:

“In the models presented here, the anisotropic discontinuity appears as a gradational change in seismic anisotropy over a depth interval, which mainly results from gradual shifts in slip system activities introduced via a linear dependence of bridgmanite CRSS on pressure.”

We also note, however, that the apparent sharpness of the discontinuity is also limited by the finite number of Lagrangian tracer aggregates used to construct the elastic medium that sample the discontinuity. Following the above sentence, we also added:

“The resolution of the discontinuity, however, is also limited by the number of Lagrangian tracer aggregates sampling the discontinuity.”,

Which is then followed by the following sentences that are already in the first revision:

“In future work, the sharpness of the anisotropic discontinuity… … transitions in bridgmanite.”

In the main text, under *Discussion*, we have now discussed the fast transition model (previously Supplementary Fig. 9, and now Supplementary Fig. 7). We agree that the term ‘unrealistic’ is ambiguous, and as such, we have therefore replaced it with a more fitting discussion by describing the resulting radial anisotropy pattern:

“One end-member scenario that can promote the development of a sharper anisotropic discontinuity is to… …that are not present in the gradual transition model (Fig. 4a)}. In natural subduction systems, several geodynamic scenarios could result in a sharper anisotropic discontinuity…”

Reviewer #3 (Remarks to the Author):

Review of “Mid-mantle discontinuities beneath subduction zones linked to depth-dependent deformation of bridgmanite”
Daniel A Frost

This paper introduces a new mechanism of generating seismic anisotropy in the uppermost lower mantle ~1000 km depth by development of texture in bridgmanite. This textural development is facilitated by increased pressure below the slab triggering a change in dominant slip system. The authors suggest that this anisotropy could cause a seismic velocity contrast at ~1000 km depth, which could generate SdS precursors which have been modelling to occur at these depths in subduction zones and in other areas of the mantle.

This paper suggests an interesting new mechanism that is motivated by mineral physics measurements of the CRSS in bridgmanite and brings together mineral physics and geodynamics to interpret seismic observations. This submission of the paper improves over the previous version by more clearly stating the limits of this phenomenon: that it could only explain mid-mantle anomalies around subduction zones, does not explain PdP reflectors, and is not the only possible cause of mid-mantle reflectors. I appreciate the addition of the synthetic seismograms and the vespagrams to confirm the theoretical existence of these phases. My main remaining question is about whether the amplitude of the synthetic SdS wave matches that from data. Also, the sentence structure used can be confusing but could be improved by breaking up the long sentences. I have highlighted some examples in this review.

We thank you for your thorough comments. We have now revised the *Results* section to clarify the modeled amplitude of the synthetic S^dS phase generated by the anisotropic discontinuity and how it fares relative to mid-mantle S^dS observations. We have also revised the manuscript to improve overall sentence structure and figure presentation for better readability.

General comments

1. How strong are the SdS waves generated by the anisotropy contrast? Studies such as Waszek et al., (2018) stack multiple traces and find SdS precursors of amplitudes around half that of transition zone phases S440S and S660S.

We thank you for raising this point. In our full waveform simulations, the predicted S^1480S/SS amplitude ratio is 0.0084. This exceeds the global detectability threshold of 0.0065 reported by Waszek et al. (2018), although only slightly, indicating that the modeled phase is weak but potentially detectable using array seismological methods. We note, however, that the modeled amplitude represents a conservative estimate for the specific anisotropic structure considered here. As discussed in the manuscript, several geophysical scenarios could enhance the effective anisotropic discontinuity and may therefore increase the reflected amplitude. Nevertheless, we stress again that the S^1480S/SS amplitude ratio modeled here exceeds this threshold and is therefore potentially detectable based on our synthetic waveform modeling results. In the *Results* section under *Detectability using array analysis of synthetic SS precursors* we have now included:

"Fig. 3 shows slowness vespagrams of the processed synthetics. For a purely anisotropic mantle model without any isotropic perturbations coming from the slab, the anisotropic discontinuity generates a clear S^{d}S arrival at 112° (Fig. 3a) where we find the S^{d}S/SS amplitude ratio to be 0.0084. Although this phase is weak, its S^{d}S/SS amplitude ratio exceeds the global detectability threshold of 0.0065 reported by ref.\citep{waszek2018global}."

2. I think that the authors need to explain their definition of slab stagnation. For instance, in Figure 6, I view both of these scenarios as stagnation. These scenarios differing only in the depth of stagnation, either above 660 km or above 1000 km. This relates to my comment below about line 168.

We thank you for this helpful comment. Indeed, we agree in a broad sense that both scenarios in Fig. 6 may be described as slab stagnation that differs only in the depth at which flattening occurs. For a more nuanced comparison, we now refer to the former 'stagnant slab model' as the 'shallowly stagnating slab model'. On the other hand, we retain the term 'penetrating slab model' as it adequately describes slabs that have crossed the 660-km boundary. The changes in terminology have now been reflected throughout the manuscript and in the supplementary material where appropriate.

3. Is anisotropy of olivine above 660 km depth neglected throughout these calculations? How would shallow mantle anisotropy affect the path-integrated shear wave splitting?

We thank you for raising this point. In our shear wave splitting modeling, we do not include anisotropy relevant to the olivine system above 660 km. The modeled path-integrated effective splitting parameters therefore reflect only the strain-induced anisotropy of P-dependent bridgmanite in the lower mantle. We acknowledge that upper-mantle anisotropy in the receiver side can contribute to the path integrated splitting. However, our numerical experiments are designed to be analogous to source-side shear wave splitting methodologies for real data, in which receiver-side upper-mantle anisotropy is either corrected using independently constrained *KS (where * can be P/S) splitting parameters, or no correction is applied where the receiver side is characterized as null (e.g., effectively isotropic). In such practices, the remaining splitting

measurements are interpreted as source-side anisotropy near the slab (e.g., Lynner and Long, 2014; Mohiuddin et al., 2015). In addition, our models are analogous to measurements from deep events (>500 km) for which source-side ray paths sample the lower mantle transition zone (520–660 km), where ringwoodite is expected to be effectively isotropic owing to its cubic structure (e.g., Mainprice et al., 2000; Carrez et al., 2007; Faccenda et al., 2014; Ferreira et al., 2019; Wolf et al., 2024).

Line-by-line comments

Line 41: The following sentence implies that SS reflectors only occur in subduction zones, which is not true. I suggest rewriting this to remove that implication. "In subduction settings, reflectors and scatterers within this depth range are well-documented using SS precursors (i.e., S-wave underside reflections off depth d)4–7"

Thank you for your comment. We have now revised this sentence to remove this implication: "Seismic reflectors within this depth range have been documented globally using SS precursors (i.e., S-wave underside reflections off depth d)\citep{deuss2002systematic,deuss2009global,waszek2018global}, hereafter referred to as S^{d}S, including beneath subduction zones\citep{yuan2025mantle,yuan2026revealing} where scattering of converted waves have also been observed\citep{vinnik2001search,kaneshima2009seismic,niu2003seismic,haugland2017estimate,kaneshima2019seismic,yuan2021southeastward"

Lines 75-75: These feel out of place since anisotropy resulting from Bridgmanite was introduced in the prior paragraph.

We thank you for pointing this out. Lines 75-75 have been retained. On the other hand, the preceding text in Lines 67-70 have been restructured using more general phrasing. In this way, Bridgmanite CPO as cause for lower-mantle anisotropy is introduced more naturally in the succeeding paragraph:

"In the lowermost mantle ($>$2800 km), anisotropic layering has also been proposed as a possible explanation for deep reflections and shear wave splitting adjacent to large-low velocity provinces."

Line 120: Suggest changing "with distance" to "with distance away from the slab"

Corrected. The sentence now reads:

"When the entrained mantle accumulates strain due to slab-driven deformation, the P-induced slip transitions in bridgmanite mainly produce an anisotropic discontinuity around the slab that progressively weakens with distance away from the slab into the surrounding mantle."

Line 168: The authors imply that no stagnating slab could not generate anisotropy by this mechanism. I don't feel that this is sufficiently tested to draw this conclusion. The authors test a slab that stagnates at 660 km depth, therefore has only just passed into the anisotropy-forming region. However, some slabs, such as Tonga and South America (Fukao and Obayashi, 2013) are observed to stagnate at 1000 km, similar to the depth in their main subduction model. I think instead stating that shallowly stagnating slabs could not generate this anisotropy would be a more reasonable claim.

We thank you for this suggestion. In relation to our comment above, we now refer to the previous stagnant slab model as the shallowly stagnant slab model.

Lines 174-176: Why are arrays necessary? Is this because the amplitudes of these phases are so low?

Indeed, S^dS phases are typically weak and difficult to identify, whether they arise from the anisotropic discontinuity considered here or from isotropic interfaces associated with thermo-

chemical boundaries or phase transitions (e.g., Deuss, 2009) for which array methods are useful to enhance such signals especially those originating from the mid-mantle (e.g., Saki et al., 2022; Rochira and Thomas, 2023; Yuan, Magali et al., 2025,2026). Vespagram analysis, for example, can further help distinguish S^dS phases from other arrivals using not only their travel times, but also their slowness characteristics (e.g., Rost and Thomas, 2002). We have now rephrased the sentence to:
“Determining whether such reflection amplitudes are visible in seismic data requires array analysis to help identify the expected weak S^{d}S signal generated by the anisotropic discontinuity.”

Line 232: What “delay times” are being referred to? Supplementary Figure 6 also does not describe what delay times are being used.
Thank you for raising this point. For clarity, Supplementary Fig. 6 figure caption now reads:
“\textbf{Observed delay times for subslab anisotropy beneath Western Java compiled from different studies.} Comparison of delay times predicted for our penetrating slab anisotropy model (line plots) with observed delay times in the subslab mantle underneath Western Java (scatter plots) as a function of epicentral distance.”

Lines 232-235 in the main text have also been revised:
“The delay times computed for our anisotropy model, however, appear underestimated (line plots in Supplementary Fig. 6). This discrepancy likely reflects the inherently large uncertainties surrounding its estimates in our modeling and in the observations:”

Lines 247-250: I can’t fully understand the following sentence “Finally, sourceside SWS assumes similar receiver-side ray paths for S and SK(K)S phases. This assumption may break down at shorter epicentral distances wherein the estimates become potentially vulnerable to contamination from receiver-side anisotropy.”.
Thank you for raising this point. We now address points 1 and 2 below.

1- How would shorter epicentral distances cause more contamination by receiver-side anisotropy than source-side anisotropy? If greater epicentral distance data are more accurate, could the data be filtered by epicentral distance to reduce bias?
Shorter epicentral distances do not necessarily cause greater receiver-side anisotropy than in the source-side. However, shorter distances can increase uncertainty when correcting for receiver-side anisotropy. Source-side SWS relies on using *KS phases to characterize receiver-side anisotropy under the working assumption that the ray paths of the direct S and *KS are similar (i.e., similar incidence angles beneath the receiver). As epicentral distance decreases, the incidence angle of direct S increases, causing its receiver-side paths to deviate further from the near-vertical *KS paths leading to additional uncertainties when correcting for non-null receiver-side splits. Ideally, considering larger distances would indeed reduce this trade-off. This, however, is not always the case because of limited data coverage. As such, wider distance ranges (e.g., 40 – 90º) is favored as it increases the area covered to characterize source-side anisotropy, which is what is being done in current practice (e.g., Lynner and Long, 2014; Mohiuddin et al., 2014; Nowacki et al., 2015).

2- It’s not clear if this sentence refers to modelling or observations. Is the reason for this sentence to imply that the observations are not accurate representations of the true anisotropy?
Lines 247-250 refer to potential uncertainties in delay time observations assuming there is receiver-side anisotropy. We have now revised Lines 247-250 for clarity:

"Finally, source-side SWS \add{observations} assume similar receiver-side ray paths for S and SK(K)S phases \add{to correct for receiver-side anisotropy}. At shorter epicentral distances, the incidence angle of S increases and hence its receiver-side path may deviate further from the SK(K)S path which introduces additional uncertainties when correcting for non-null receiver-side anisotropy}\citep{lynner2014sub}."

Line 282: The implication of the following sentence is unclear. "In the upper MTZ, the olivine–wadsleyite and wadsleyite–ringwoodite transitions act as textural "filters" where coherent textures serve as host sites in the growth of incoherent textures with faster nucleation rates78,79." What are these nucleation rates faster than? Does it mean that a random texture can grow quickly and overgrow a pre-existing preferred orientation? If so, I would recommend stating that.
Thank you for this suggestion. We have now revised the sentence to:
"In the upper MTZ, the olivine–wadsleyite and wadsleyite–ringwoodite transitions act as textural "filters" where randomly oriented grains may overgrow and weaken a pre-existing texture inherited from the parent phase"

Lines 287-289: Repetition from earlier discussion of slab stagnation. This line also relates to my earlier comment about how slab stagnation at depths deeper than 660 km may still cause texturing.
Thank you for pointing this out. We have now completely removed Lines 287-289. A snippet of this paragraph now reads:
"Within the slab, metastable olivine may still modulate anisotropy indirectly by altering slab dynamics. Its persistence enhances down-dip compression and positive buoyancy above the ~ 660 km boundary, promoting shallow slab stagnation\citep{pokorny20232d,schmeling1999influence}. Because metastable olivine is expected mainly in old, thick lithosphere\citep{schmeling1999influence}, such sites may be excluded in the search for mid-mantle reflectors associated with anisotropic discontinuities."

Lines 479-483: Are these coordinates [lon, lat] pairs or [lat, lon] pairs?
These are [lat,lon] pairs. We have now clarified this in the *Methods*.

Figures and Tables
Figure 3: Recommend highlighting the important phases in the vespagrams, maybe with a box.
Thank you for this suggestion. We have now boxed the arrivals for emphasis.

Spelling and Grammar
Line 16: Add "The" before the word "bulk".
Added.

Line 234: What is the "its" in the phrase "surrounding its estimates"?
It refers to the delay times. We have now clarified this.

Lines 238-239: The following sentence is very long and confusing. I suggest rewriting this.
"Assuming the mantle velocity gradients permit spatially-coherent fast axis orientations from these minerals, their effects could interfere constructively among layers with different fabrics, leading to increased delay times."
We have now revised the sentence to:
"Assuming these minerals develop spatially uniform fast-axis orientations, they may also contribute to the path-integrated splitting signal. Constructive interference among anisotropic layers with different fabrics could then increase the observed delay times."

Lines 252-253: I suggest rewriting the following sentence from “Because the frequency band considered in our waveform modeling overlaps with the dominant
253 frequency of SdS, whether the discontinuity persists at higher frequencies is beyond this work.” To “We model frequencies matching the dominant observed frequency of SdS. Whether the discontinuity persists at higher frequencies is beyond this work.”
Thank you for this. We have now revised this sentence following your suggestion.

Lines 267-269: I suggest rewriting the following sentence from “This localizes larger strains beneath it which could help form stronger (100)[00] textures46, but this time, over nearly untextured bridgmanite across the viscosity jump.” To “This localizes larger strains beneath the slab which could help form stronger (100)[00] textures sharply contrasting with nearly untextured bridgmanite below the viscosity jump.”
We again thank you for this suggestion. The sentence has been revised accordingly.

Line 277: Change “accommodation respond” to “accommodation to respond”
Corrected.

Lines 292-293: The first clause of the following sentence is negated by the later detail “Barring full penetration into the lowermost mantle, subduction behavior is typically classified into three modes: (1) penetration, (2) stagnation near 660 km, and (3) trapping near 1000 km”. I suggest rewriting this as “Subduction behavior is typically classified into three modes: (1) penetration, (2) stagnation near 660 km, and (3) trapping near 1000 km.”
Corrected.

Line 356: Change “harmonic averaged” to “harmonically averaged”
Corrected.

Line 473: Change “wit” to “with”
Corrected.

Reviewer #4 (Remarks to the Author):

The authors provide extensive modeling for the potential generation of anisotropy at lower mantle depths beneath subducting slabs. Their work is useful, and could provide a fruitful avenue for understanding the dynamics of certain regions in the lower mantle. I was not an original reviewer, but I think the authors have done an adequate job of responding to reviewer comments and suggestions. I have only minor comments that I think could improve readability.
We thank you for accepting to review our manuscript, and for your positive assessment of our work. We also appreciate the constructive comments aimed at improving the readability of the manuscript. We have now addressed them point-by-point below.

Mid-mantle discontinuities beneath subduction zones linked to depth-dependent deformation of bridgmanite – Review
Abstract
- Line 16 – “The bulk of...” rather than “Bulk of...”

Corrected.

Introduction
- Line 57 – Remove “of” between “thermo-chemical” and “origin”

Corrected.

Shear Wave Splitting Calculations

- Could the authors expound a bit more on their calculation of effective shear wave splitting? I'm somewhat confused as to how the suggested geometry would replicate source-side splitting.

We thank you for raising this point. To mimic the geometry reminiscent of source-side shear wave splitting (SWS), we first consider the epicentral distance range (e.g., 50-90º) over which SWS is typically measured using direct S waves. We then use TauP and PREM to calculate the theoretical slowness of the direct S phases and convert epicentral distances into incidence angles. We then use these incidence angles, together with the corresponding propagation azimuths to compute the effective splitting produced by the representative susblab anisotropy layer using MSAT (Walker and Wookey, 2012). Thus, the calculation is designed to mimic the propagation-direction dependence of source-side SWS, for instance, the downdip and updip direct S ray paths sampling the same modeled subslab anisotropy domain with different incidence angles (i.e., epicentral distances), producing distance-dependent effective splitting parameters in a given azimuth. The simplification is that the anisotropic structure itself is represented by a local layered profile through the subslab mantle. Here, we adapted the approach of Pisconti et al., (2019, 2023), only this time, applied to direct S phases instead of ScS as in their work. We have modified the *Methods* to clarify this procedure.

- Line 428 – I think the figure reference is wrong here. My guess is that it's meant to be "Fig. 5a: Left panel"

Corrected.

- Line 431 – The authors might consider clarifying that there is no interference from slab anisotropy because the slab is above 660 km at this point

We thank you for this suggestion. We have now clarified this sentence.

Anisotropic Reflection Calculations

- I would recommend that the authors merge this section with the section on the same topic in the Supplementary Material. I don't think it makes sense to have it in both places, especially as there is information in the Supplemental Material that would help readers to better understand what is being done.

We thank you for this suggestion. We have now merged both sections under *Methods.*

- Supplementary Figure 12 is cited in neither location, but I think flagging it for readers would help them to understand your process.

The previous Supplementary Fig. 12 (now Supplementary Fig. 10) has now been properly cited in the *Methods* section.

Figures

- Figure 2a – What are the white contours showing? Based on a comment in the paper, I think it's the natural strain. But it's not indicated in the caption.

Thank you for pointing this out. It is indeed the natural strain. We have now added the following in the Figure caption:

"Solid white lines mark isocontours of the natural strain E_0"

- Figure 2b – I think it will help readers if you also label the 0° azimuth on these and other figures with the same layout. Most readers will assume that 0° is at the top of the plot, but from your caption it seems like it's at the right

In the reflection coefficient pole figures, the x axis points upward (please see coordinate axis inset beside the color bar). For all plots that show pole figures, we have now modified the coordinate axes insets to include the azimuthal direction.

- Figure 5c – The caption for this figure says, “Spherical plot of S-wave anisotropy at a chosen location in the subslab mantle.” Which location? I think that should be indicated somewhere.

We thank you for pointing this out. The location shown in Fig. 5c corresponds to an elastic tensor extracted from the representative subslab anisotropy profile in Fig. 5a at a depth of ~900 km. To make this clear, we have now marked this point with a hollow square along the profile in Fig. 5a and updated the Fig. 5c caption to refer explicitly to this marked location: “Spherical plot of S-wave anisotropy from an elastic tensor extracted from a point along the subslab depth profile (dark hollow square in \textbf{a}).”

We thank you and the two referees for the positive assessment of our manuscript for publication in *Nature Communications*. We have now addressed the remaining comments and incorporated the final suggestions for improving the manuscript. This document contains our point-by-point response to the referee comments. Here, the referee comments are shown in black, our response in blue, and excerpts from the revised manuscript in red.

Review of "Mid-mantle discontinuities beneath subduction zones linked to depth-dependent deformation of bridgmanite"
Daniel A Frost

This paper introduces a new mechanism of generating seismic anisotropy in the uppermost lower mantle ~1000 km depth by development of texture in bridgmanite. This textural development is facilitated by increased pressure below the slab triggering a change in dominant slip system. The authors suggest that this anisotropy could cause a seismic velocity contrast at ~1000 km depth, which could generate SdS precursors which have been modelling to occur at these depths in subduction zones and in other areas of the mantle.
The paper has been further improved by this second revision. I am mostly satisfied with the changes made. I have a few comments and recommended changes.
We appreciate the positive assessment of our revised manuscript. We also thank you for your final comments and suggestions for further improving the manuscript.

General comments
1. While the authors have changed their terminology somewhat to describe a "shallowly stagnating slab", the main model they show in Figure 1 still looks like a stagnating slab, despite it being described as a "penetrating slab". I feel that readers may be confused by this terminology.
We thank you for your comment. We have now changed the terminology to "deeply flattened slab".

2. The authors responded to my previous comment about olivine anisotropy being ignored here but did not change the text. I feel that this is an important point to make clear in the text, especially since the anisotropy of other minerals is introduced in line 248. Olivine, the main contributor to mantle anisotropy, is conspicuous by its absence. A sentence describing the authors' reason for ignoring this feels important.
We thank you for pointing this out. Following our response from the previous round, we have now added two sentences clarifying our modeling choices under *Shear wave splitting calculations* in the *Methods* section:
"We do not include olivine anisotropy above 660 km depth in these calculations. Our modeling is thus analogous to source-side SWS analyses where receiver-side upper-mantle anisotropy is corrected or characterized to be effectively isotropic using *KS (where * can be P or S waves) splitting \citep{lynner2014sub,mohiuddin2015mid}"

In conjunction, we have also slightly reworded the lines 247-248 to:
"In the subslab mantle, other minerals may coexist \add{within the modeled depth range (see Methods)} such as akimotoite\citep{akaogi2002garnet} and phase D\citep{ohtani2005water}"

Line-by-line comments
Lines 189-191: I think that the following sentence could be revised to improve clarity, "For a purely anisotropic mantle model without any isotropic perturbations coming from the slab, the anisotropic discontinuity generates a clear SdS arrival at 112◦ (Fig. 3a)". I suggest something like, "For a model with only anisotropic velocity contrasts from induced texture and no isotropic velocity

perturbations resulting from the slab, the anisotropic discontinuity caused by the slab generates a clear SdS arrival at 112◦ (Fig. 3a)".
We thank you for your suggestion. Lines 186-189 have now been revised to:
"For a model that includes only anisotropic velocity contrasts from the induced texture and no isotropic velocity perturbations associated with the slab, the resulting anisotropic discontinuity generates a clear S^{d}S arrival at 112$\circ$ (Fig. 3a)"

Line 194: Suggest altering the following phrase, "but with a reversed SdS polarity relative to SS".
Corrected.

Line 246: The authors have added a little more detail to describe the delay times, but I recommend describing what the delay times are of. I think it's the anisotropic delay time between the SV and SH S-wave, but it is not clear.
Thank you for pointing this out. We have now clarified this in line 244:
"The delay times between the fast and slow quasi-S waves computed for our anisotropy model, however, appear underestimated (line plots in Supplementary Fig. 6)."

Figures and Tables
Figure 2. Recommend adding "α" to figure caption and colour scale label in 2a.
Corrected.

Spelling and Grammar
Line 47: "The variability is unclear whether the signals reflect coherent discontinuities or instead originate". The grammar here appears incorrect, but I don't know how as I can't determine what is intended.
Corrected. The sentence now reads:
"It is unclear whether the signals originate from coherent discontinuities or instead produced by small-scale heterogeneities that scatter seismic energy."

Line 51: In "However, this interpretation" suggest changing "this interpretation" to "these interpretations"
Corrected.

Line 220: The following sentence appears incorrect, "Previous studies using (applying?) kinematic approaches to (model?) mantle fabric calculation explored various".
Corrected.

Comments on “Mid-mantle discontinuities beneath subduction zones linked to depth-dependent deformation of bridgemanite” by Magali et al.

This paper reports on the combination modelling of important processes associated with the deformation of the Earth’ lower mantle near subduction zones. The main idea proposed by the authors, discontinuous change in anisotropic structure as the cause of seismic anomalies in the mid-lower mantle, is interesting. I think this paper deserves publication in this journal. In general, the manuscript is adequately structured and the descriptions in the main text seem succinct, maybe too succinct forced by the tight length limit of the article of the journal. Here I raise several issues to be clarified further before accepting this paper. Most of my comments seem to be related to the meaning of “sharpness” of the discontinuity structure, which is not necessarily clearly presented throughout the manuscript.

1. Line 124-127 “In the mantle wedge and sub slab region, …. Coincidentally, this is also … ”

I was a bit puzzled by reading these sentences. Is the agreement between the discontinuity and the depth where the CRSS values of the (100)[001] and (010)[100] slip systems switch really a coincidence? They are expected to coincide with each other in the framework of the authors’ model, aren’t they?

Also does the first sentence refer to Figure 2a? If so, the argument of the sentence is not necessarily validated by the figure, I think.

2. Line 129-133 “Its sharpness is well correlated with increasing deformation in the surrounding mantle despite the smooth transition in bridgmanite slip systems with pressure”

The meaning of this sentence is not clear. It is perhaps because the term “sharpness” is not very well defined.

3. Line 120-123 “… where anisotropy index is drastically reduced.”

This comment is related to the previous one. Figure 2a gives me an impression that reduction of the anisotropy index occurs very gradually. Please discuss the sharpness of the anisotropy discontinuity more quantitatively, if possible.

4. Line 143-144 “We base our computations on SdS, …, enables sensitivity to even subtle discontinuities, …”

The meaning of this sentence is not clear, either. Do the authors claim that, although the discontinuity structure of their dynamic model is subtle and smooth, the waveform analyses

method they use enables detecting the structure?

4. Line 439- Section “Anisotropic reflection calculations “

By reading the Method section of the manuscript I had an impression that in their model each reflector is regarded as a first order discontinuity not as a gradual transition in the anisotropy index. But the descriptions are not very clear-cut about this point, so please clarify this point. Please also clarify whether the dip of the reflector is considered or modelled as a horizontal interface.

5. Line 131 “Locations A and B exhibit a sharper contrast…”

I was a bit confused by the sentence, because it refers to the absolute values of anisotropy index α , not its contrast. Please clarify this point.

6. Line 413-418 “… the predicted anisotropy produced by an instantaneous change…”

The meaning of these sentences was not clear. Do they mean the model predict only a gradual change in the anisotropy structure? What does the term “transition” mean? Referring here to Supplementary Fig.9 is not kind to potential readers, I think. The description including “…unrealistic anisotropy patterns …” is not very understandable, either. Somewhat a short and clear-cut explanation is desirable in the main text.

Mid-mantle discontinuities beneath subduction zones linked to depth-dependent deformation of bridgmanite – Review

Abstract

- Line 16 – "The bulk of…" rather than "Bulk of…"

Introduction

- Line 57 – Remove "of" between "thermo-chemical" and "origin"

Shear Wave Splitting Calculations

- Could the authors expound a bit more on their calculation of effective shear wave splitting? I'm somewhat confused as to how the suggested geometry would replicate source-side splitting.
- Line 428 – I think the figure reference is wrong here. My guess is that it's meant to be "Fig. 5a: Left panel"
- Line 431 – The authors might consider clarifying that there is no interference from slab anisotropy because the slab is above 660 km at this point

Anisotropic Reflection Calculations

- I would recommend that the authors merge this section with the section on the same topic in the Supplementary Material. I don't think it makes sense to have it in both places, especially as there is information in the Supplemental Material that would help readers to better understand what is being done.
- Supplementary Figure 12 is cited in neither location, but I think flagging it for readers would help them to understand your process.

Figures

- Figure 2a – What are the white contours showing? Based on a comment in the paper, I think it's the natural strain. But it's not indicated in the caption.
- Figure 2b – I think it will help readers if you also label the 0° azimuth on these and other figures with the same layout. Most readers will assume that 0° is at the top of the plot, but from your caption it seems like it's at the right
- Figure 5c – The caption for this figure says, "Spherical plot of S-wave anisotropy at a chosen location in the subslab mantle." Which location? I think that should be indicated somewhere.

Review of "Mid-mantle discontinuities beneath subduction zones linked to depth-dependent deformation of bridgmanite"

Daniel A Frost

This paper introduces a new mechanism of generating seismic anisotropy in the uppermost lower mantle ~1000 km depth by development of texture in bridgmanite. This textural development is facilitated by increased pressure below the slab triggering a change in dominant slip system. The authors suggest that this anisotropy could cause a seismic velocity contrast at ~1000 km depth, which could generate SdS precursors which have been modelling to occur at these depths in subduction zones and in other areas of the mantle.

The paper has been further improved by this second revision. I am mostly satisfied with the changes made. I have a few comments and recommended changes.

General comments

1. While the authors have changed their terminology somewhat to describe a "shallowly stagnating slab", the main model they show in Figure 1 still looks like a stagnating slab, despite it being described as a "penetrating slab". I feel that readers may be confused by this terminology.

2. The authors responded to my previous comment about olivine anisotropy being ignored here but did not change the text. I feel that this is an important point to make clear in the text, especially since the anisotropy of other minerals is introduced in line 248. Olivine, the main contributor to mantle anisotropy, is conspicuous by its absence. A sentence describing the authors' reason for ignoring this feels important.

Line-by-line comments

Lines 189-191: I think that the following sentence could be revised to improve clarity, "*For a purely anisotropic mantle model without any isotropic perturbations coming from the slab, the anisotropic discontinuity generates a clear S_dS arrival at 112°(Fig. 3a)*". I suggest something like, "*For a model with only anisotropic velocity contrasts from induced texture and no isotropic velocity perturbations resulting from the slab, the anisotropic discontinuity caused by the slab generates a clear S_dS arrival at 112° (Fig. 3a)*".

Line 194: Suggest altering the following phrase, "*but with a reversed S_dS polarity relative to SS*".

Line 246: The authors have added a little more detail to describe the delay times, but I recommend describing what the delay times are of. I think it's the anisotropic delay time between the SV and SH S-wave, but it is not clear.

Figures and Tables

Figure 2. Recommend adding "α" to figure caption and colour scale label in 2a.

Spelling and Grammar

Line 47: "*The variability is unclear whether the signals reflect coherent discontinuities or instead originate*". The grammar here appears incorrect, but I don't know how as I can't determine what is intended.

Line 51: In "*However, this interpretation*" suggest changing "this interpretation" to "these interpretations"

Line 220: The following sentence appears incorrect, "*Previous studies using (applying?) kinematic approaches to (model?) mantle fabric calculation explored various*".
